# Supplementary material for: Interventions to address empathy-based stress in mental health workers: A scoping review and research agenda
Source: PLoS One. 2024 Dec 5;19(12):e0306757. doi: 10.1371/journal.pone.0306757 (PMC11620613; doi:10.1371/journal.pone.0306757)
Supplement: S1 File — (DOCX) [file pone.0306757.s001.docx]

**Supporting Information**

**S1: Preferred Reporting Items for Systematic reviews and Meta-Analyses extension for Scoping Reviews (PRISMA-ScR) Checklist**

| **SECTION** | **ITEM** | **PRISMA-ScR CHECKLIST ITEM** | **REPORTED ON PAGE #** |
| --- | --- | --- | --- |
| **TITLE** | | | |
| Title | 1 | Identify the report as a scoping review. | ​​1​ |
| **ABSTRACT** | | | |
| Structured summary | 2 | Provide a structured summary that includes (as applicable): background, objectives, eligibility criteria, sources of evidence, charting methods, results, and conclusions that relate to the review questions and objectives. | ​​2​ |
| **INTRODUCTION** | | | |
| Rationale | 3 | Describe the rationale for the review in the context of what is already known. Explain why the review questions/objectives lend themselves to a scoping review approach. | ​​2-7 |
| Objectives | 4 | Provide an explicit statement of the questions and objectives being addressed with reference to their key elements (e.g., population or participants, concepts, and context) or other relevant key elements used to conceptualize the review questions and/or objectives. | ​​7-8 |
| **METHODS** | | | |
| Protocol and registration | 5 | Indicate whether a review protocol exists; state if and where it can be accessed (e.g., a Web address); and if available, provide registration information, including the registration number. | ​​8​ |
| Eligibility criteria | 6 | Specify characteristics of the sources of evidence used as eligibility criteria (e.g., years considered, language, and publication status), and provide a rationale. | ​​9-10​ |
| Information sources* | 7 | Describe all information sources in the search (e.g., databases with dates of coverage and contact with authors to identify additional sources), as well as the date the most recent search was executed. | ​​8, supplementary information |
| Search | 8 | Present the full electronic search strategy for at least 1 database, including any limits used, such that it could be repeated. | ​​ supplementary information |
| Selection of sources of evidence† | 9 | State the process for selecting sources of evidence (i.e., screening and eligibility) included in the scoping review. | ​​10-12 |
| Data charting process‡ | 10 | Describe the methods of charting data from the included sources of evidence (e.g., calibrated forms or forms that have been tested by the team before their use, and whether data charting was done independently or in duplicate) and any processes for obtaining and confirming data from investigators. | ​​12-13 |
| Data items | 11 | List and define all variables for which data were sought and any assumptions and simplifications made. | ​​10-12 |
| Critical appraisal of individual sources of evidence§ | 12 | If done, provide a rationale for conducting a critical appraisal of included sources of evidence; describe the methods used and how this information was used in any data synthesis (if appropriate). | ​​N/A​ |
| Synthesis of results | 13 | Describe the methods of handling and summarizing the data that were charted. | ​​13​ |
| **RESULTS** | | | |
| Selection of sources of evidence | 14 | Give numbers of sources of evidence screened, assessed for eligibility, and included in the review, with reasons for exclusions at each stage, ideally using a flow diagram. | ​Figure 2, pg 14 |
| Characteristics of sources of evidence | 15 | For each source of evidence, present characteristics for which data were charted and provide the citations. | ​​Table 4, pg 16-26 |

**S2:** **Full Search Strategies.** Initial searches run on 15.01.2021, 1970-2021 date filters used. Updated searches run on 12.09.2022, 2021-2022 date filters used.

***PsychINFO (all databases):***

  (((**Any Field**: ("compassion fatigue")) *OR* (**Any Field**: ("secondary traumatic stress")) *OR* (**Any Field**: (burnout)) *OR* (**Any Field**: (burn-out)) *OR* (**Any Field**: ("vicarious trauma")) *OR* (**Any Field**: ("occupational stress")) *OR* (**IndexTermsFilt**: ("Occupational Stress") *OR* **IndexTermsFilt**: ("Compassion Fatigue")) *OR* (**Any Field**: ("compassion stress")) *OR* **Any Field**: "secondary trauma" *OR* **Any Field**: "secondary traumatisation" *OR* **Any Field**: "secondary traumatization") *AND* ((**Year**: [1970 TO 2021]))) *AND* (((AnyField:(psychotherapist*)) OR (AnyField:("forensic nurse")) OR (AnyField:("forensic psychologist")) OR (AnyField:("forensic psychiatrist")) OR (AnyField:(therapist*)) OR (AnyField:("support time and recovery worker")) OR (AnyField:("mental health employee")) OR (AnyField:(counsellor*)) OR (AnyField:("counselling psychologist")) OR (AnyField:("family therapist")) OR (AnyField:(counselor)) OR (AnyField:("mental health support worker")) OR (AnyField:("mental health social worker")) OR (IndexTermsFilt:("Mental Health Personnel")) OR (IndexTermsFilt:("Counselors") OR OR (AnyField:("mental health care provider")) OR (AnyField:("mental health personnel")) OR (AnyField:("mental health nurse")) OR (AnyField:("mental health service")) OR (AnyField:("mental health service provider")) OR (AnyField:("mental health professional")) OR (AnyField:(psychologist*)) OR (AnyField:("mental health worker"))) AND ((PublicationYear:[1970 TO 2021] OR TestYear:[1970 TO 2021])))

***Pubmed***

Mesh search:

("social work, psychiatric"[MeSH Major Topic] OR "social work, psychiatric"[MeSH Major Topic] OR "emergency services, psychiatric"[MeSH Major Topic] OR "psychiatric nursing"[MeSH Major Topic] OR "health personnel/psychology"[MeSH Major Topic]) AND "burnout, professional/prevention and control"[MeSH Major Topic] OR "compassion fatigue/prevention and control"[MeSH Major Topic])

Keyword search:

("psychotherapist*"[Title/Abstract] OR "forensic nurs*"[Title/Abstract] OR "forensic psychologist*"[Title/Abstract] OR "forensic psychiatrist*"[Title/Abstract] OR "therapist*"[Title/Abstract] OR "mental health employee*"[Title/Abstract] OR "counsellor*"[Title/Abstract] OR "counselling psychologist*"[Title/Abstract] OR "family therapist*"[Title/Abstract] OR "counselor*"[Title/Abstract] OR "mental health support worker*"[Title/Abstract] OR "mental health social worker*"[Title/Abstract] OR "mental health care provider*"[Title/Abstract] OR "mental health personnel"[Title/Abstract] OR "mental health nurs*"[Title/Abstract] OR "mental health service*"[Title/Abstract] OR "mental health service provider*"[Title/Abstract] OR "mental health professional*"[Title/Abstract] OR "psychologist*"[Title/Abstract] OR "mental health worker"[Title/Abstract])) AND ("compassion fatigue"[Title/Abstract] OR "vicarious trauma"[Title/Abstract] OR "secondary traumatic stress"[Title/Abstract] OR "burnout"[Title/Abstract] OR "burn out"[Title/Abstract] OR "compassion stress"[Title/Abstract] OR "secondary trauma"[Title/Abstract] OR "secondary traumatisation"[Title/Abstract] OR "secondary traumatization"[Title/Abstract])

***Embase***

- #1 ('compassion fatigue'/exp OR 'compassion fatigue' OR 'compassion stress' OR 'vicarious trauma'/exp OR 'vicarious trauma' OR 'burnout'/exp OR burnout OR 'burn out'/exp OR 'burn out' OR 'secondary traumatic stress'/exp OR 'secondary traumatic stress' OR 'secondary trauma' OR 'secondary traumatisation' OR 'secondary traumatization') AND ([embase]/lim OR [embase classic]/lim) AND [1970-2021]/pyEBS &amp; MHWs 08.01.212021-01-082021-01-0814187

- #2 ('vicarious trauma'/exp OR 'burnout'/exp OR 'professional burnout'/exp OR 'compassion fatigue'/exp OR 'secondary traumatic stress'/exp) AND ([embase]/lim OR [embase classic]/lim) AND [1970-2021]/pyEBS &amp; MHWs 08.01.212021-01-082021-01-0811764
- #3 (psychotherapist* OR 'forensic nurse' OR 'forensic psychologist' OR 'forensic psychiatrist'/exp OR 'forensic psychiatrist' OR therapist* OR 'support time and recovery worker' OR 'mental health employee' OR counsellor* OR 'counselling psychologist' OR 'family therapist' OR 'counselor'/exp OR counselor OR 'mental health support worker' OR 'mental health social worker' OR 'mental health care provider' OR 'mental health personnel'/exp OR 'mental health personnel' OR 'mental health nurse' OR 'mental health service'/exp OR 'mental health service' OR 'mental health service provider' OR 'mental health professional'/exp OR 'mental health professional' OR psychologist* OR 'mental health worker'/exp OR 'mental health worker') AND ([embase]/lim OR [embase classic]/lim) AND [1970-2021]/pyEBS &amp; MHWs 08.01.212021-01-082021-01-08121632

- #4 ('mental health care personnel'/exp OR 'psychotherapist'/exp OR 'counselor'/exp OR 'mental health service'/exp OR 'psychologist'/exp OR 'psychiatric nurse'/exp OR 'forensic psychology'/exp) AND ([embase]/lim OR [embase classic]/lim) AND [1970-2021]/pyEBS &amp; MHWs 08.01.212021-01-082021-01-0856216
- #5 #1 OR #2
- #6 #3 OR #4
- #7 #5 AND #6

***PTSDPubs:***

MAINSUBJECT.EXACT("Vicarious Traumatization") OR MAINSUBJECT.EXACT("Burnout") OR MAINSUBJECT.EXACT("Trauma Contagion") OR "compassion fatigue" OR "compassion stress" OR "secondary traumatic stress" OR "vicarious trauma" OR burnout OR burn-out

AND

MAINSUBJECT.EXACT("Mental Health Personnel") OR (psychotherapist* OR "forensic nurse" OR  "forensic psychologist" OR  "forensic psychiatrist" OR therapist* OR "support time and recovery worker" OR "mental health employee" OR counsellor* OR "counselling psychologist" OR "family therapist" OR counselor OR "mental health support worker" OR "mental health social worker" OR "mental health care provider" OR  "mental health personnel" OR "mental health nurse" OR "mental health service" OR  "mental health service provider" OR  "mental health professional" OR psychologist* )

***Central:***

"compassion fatigue" OR "compassion stress" OR "secondary traumatic stress" OR "vicarious trauma" OR burnout OR burn-out OR "Compassion Fatigue" OR "burnout, professional" OR "burnout, psychological" OR "burnout, professional/prevention and control" OR "compassion fatigue/prevention and control" in Title Abstract Keyword AND 'social work, psychiatric' OR 'emergency services, psychiatric' OR 'psychiatric nursing' OR 'health personnel, psychology' OR "psychotherapist*" OR "forensic nurs*" OR "forensic psychologist*" OR "forensic psychiatrist*" OR "therapist*" OR "mental health employee*" OR "counsellor*" OR "counselling psychologist*" OR "family therapist*" OR "counselor*" OR "mental health support worker*" OR "mental health social worker*" OR "mental health care provider*" OR "mental health personnel" OR "mental health nurs*" OR "mental health service*" OR "mental health service provider*" OR "mental health professional*" OR "psychologist*" OR "mental health worker" in Title Abstract Keyword - with Cochrane Library publication date Between Jan 1970 and Jan 2021

**S3: Table showing studies reporting participant ethnicity**

| **Study** | **Ethnicity of Participants** |
| --- | --- |
| (55) | White: 18 (69%)  Black: 2 (8%)  Asian: 3 (12%)  ‘Other’ ethnic backgrounds: 2 (8%). |
| (56) | White: 111 (77%) |
| (58) | African American: 8  Caucasian: 7  ‘Other’ ethnic backgrounds: 1 |
| Boone [Unpublished] | White:44 (83%)  Black: 4 (7.5%)  Hispanic: 5 (9.4%) |
| (60) | Chinese: 29 (78.4%) Malay: 2 (5.4%) Indian: 4 (10.8%) |
| Wymer, [Unpublished] | African American/Black: 1 (33.3%)  Hispanic: 1(33.3%)  White: 1 (33.3%) |
| (65) | White: 100% |
| (66) | White British: 95% (no information on other 5%) |
| (71) | African American: 18 (26.1%) White: 43 (62.3%) Multiracial: 3 (4.4%) ‘Other’ ethnic backgrounds: 1(1.4%) Missing data: 4 (5.8%) |
| (72) | White: 94.7% (no information on other 5.3%) |
| (73) | White: 30 (66.7%)  'Non-white': 15 (33.3%) |
| (74) | Caucasian/White: 21 (70%)  Hispanic/Latina: 3 (10%)  African American/Black: 1 (3.3%)  Mixed race: 1 (3.3%)  ‘Other’ ethnic backgrounds: 4 (13.3%) |
| (78) | White: 19 (95%)  Hispanic/Latino: 1 (5%) |
| (81) | Black African: 55 % White British: 16 % African Caribbean: 9% Black British: 8% Mauritian: 7% Indian: 3% White African: 2% |
| (82) | White: 62 (79%)  Black:15 (19%)  ‘Other’ ethnic backgrounds: 2 (3%).  Hispanic: 3 (4%) |
| (83) | African American: 29 (19.7%)  Asian/Pacific Islander: 1 (0.7%)  White: 98 (65.3%)  Hispanic/Latino: 12 (8.2%)  ‘Other’ ethnic backgrounds: 9 (6.1%) |
| (84) | Not given; however, sample reportedly the same as Weingardt et al. (2009), plus two further participants |
| (86) | White: 84% |
| (88) | Asian American: 14.7%  Latina: 18.5%  White: 66.7% |
| Ballew [Unpublised] | Caucasian: 35 (87.5%)  Multiracial: 3 (7.5%)  American Indian: 1 (2.5%)  Other: 1 (2.5%) |

**S4: Table showing quantitative measures of EBS used**

| **Measure** | **Description** | **No. of Studies Used In** |
| --- | --- | --- |
| The Maslach Burnout Inventory (MBI) (Maslach & Jackson, 1981). | The MBI is a 22-item self-report measure of burnout in human services professionals. It produces 3 dimensions of burnout: emotional exhaustion, depersonalization, and personal accomplishment. It is the most commonly used measure of burnout with high reliability (α = .71 or higher) across 7 countries (Poghosyan et al., 2009). | 24 |
| The Dutch version of the MBI (DMBI) (Schaufeli, 1990). | The questionnaire consists of 22 items on a 7-point Likert scale. The Dutch MBI has been shown to be valid and reliable in the assessment of burnout in nurses (Schaufeli & van Dierendonck, 1993). | 1 |
| The Arabic version of the MBI (Al-Turki  et al., 2010; Hamaideh,  2011). | The Arabic MBI has been used in a number of Arabic-speaking countries, in  studies assessing burnout levels among nurses (Al-Turki et al., 2010; Hamaideh, 2011). It has been shown to have good internal consistency across subscales: α = .80 for emotional exhaustion; α =.73 for depersonalisation; α =.77 for personal accomplishment (Sabbah et al., 2012). | 1 |
| The Italian version of the MBI (Sirigatti & Stefanile, 1993). | This Italian version of the MBI has been shown to have good internal consistency across subscales: α = .88 for emotional exhaustion; α =.70 for depersonalisation; α =.83 for personal realisation (Maslach & Jackson, 1981; Sirigatti & Stefanile, 1993). | 1 |
| MBI-General Survey (MBI-GS) (Schaufeli et al., 1996). | The MBI-GS consists of generic  items which measure burnout dimensions and can be used in a variety of occupations  The MBI-GS is a 16-item self-report measure of burnout in any occupation (not just health/care). The generic items measure burnout on 3 subscales that parallel the MBI: exhaustion, cynicism, and  professional efficacy. The MBI-GS has been shown to have satisfactory internal consistency (α = .84 to .90 for exhaustion, .74 to .84 for cynicism, and from .70 to .78 for professional efficacy) (Leiter & Schaufeli, 1996). | 1 |
| The Copenhagen Burnout Inventory (CBI) (Kristensen et al., 2005). | The CBI is a 19-item self-report tool that produces 3 scales measuring personal burnout, work-related burnout, and client-related burnout. It is designed for use with employees in any sector. A prospective study of burnout in human service workers sector found that all 3 scales had high internal reliability α =.87 for both personal and work-related burnout; α = 0.85 for client-related burnout) (Borritz et al., 2006). | 3 |
| The Oldenburg Burnout Inventory (OLBI) (Demerouti & Nachreiner, 1998). | The OLBI is a self-report measure consisting of 16 positively and negatively formulated items that are used to evaluate 2 dimensions of burnout: the identification continuum (ranging from disengagement to dedication) and the energy continuum (ranging from exhaustion to vigour). Studies have demonstrated the convergent validity of the OLBI and the MBI-General Survey (Demerouti et al., 2003; Halbesleben & Demerouti, 2005). The reliability of the exhaustion subscale ranges from α = .74 to α = .85, and the reliability of the disengagement subscale from .73 to .85 (Demerouti & Bakker, 2008; Demerouti et al., 2003; Halbesleben & Demerouti, 2005, Halbesleben, 2010; Sonnentag, Binnewies & Mojza, 2010; Timms et al., 2012). | 2 |
| Silencing Response Scale (SRS) (Baranowsky, 2002). | The SRS is a 15-item scale that helps care-givers identify specific communication difficulties in their trauma work with clients i.e. selective listening and active avoidance. It has been shown to have good reliability, α = .85 (Ortlepp & Friedman, 2001). | 1 |
| The Trauma Recovery Scale [Unpublished] | The TRS is a 10-item self-report scale. Parts I & II provide a traumatic experiences inventory. Part III measures relative recovery from traumatic experiences. It is found to have good internal reliability (*a* = .86) and good convergent validity with the impact of events scale (.71). (Gentry, [Unpublished]) | 1 |
| Secondary Traumatic Stress Scale (STSS) Bride et al., 2004). | The STSS is a 17-item self-report measure of secondary trauma in practitioners who have experienced traumatic stress through their work with clients. It comprises 3 subscales; Intrusion, Avoidance, and Arousal. It was found to have good internal consistency, ranging from α = .93- 94 for the total STSS scale; α = .79-80 for the Intrusion subscale, α = .85- 87 for the Avoidance subscale, and α = .83-87 for the Arousal subscale (Bride et al., 2004; Ting et al., 2005). | 1 |
| The Impact of Events Scale (IES) (Horowitz et al., 1979). | The IES is a 15-item self-report measure of current subjective distress for any specific life event. It measures distress along two subscales: Intrusive and Avoidance symptoms. The IES has shown very good internal consistency (*r _=_* .79 to .92, with an average of .86 for the intrusive subscale and .90 for the avoidance subscale) (Corcoran and Fischer, 1994). The split-half reliability of the IES scale was high (*r* =.86) and internal consistency of the subscales was also high: α =.78 for intrusion and α =.82 for avoidance. Test-retest reliability was high: *r=* .87 for the total stress scores; .89 for the intrusion subscale; and .79 for the avoidance subscale (Horowitz et al., 1979). | 1 |
| The questionnaire on the perception of occupational burnout (Penko, 1994). | According to the information in Kovač et al (2016), this questionnaire consists of 3 dimensions of occupational burnout: less fulfilment, emotional exhaustion and depersonalization. No further information was accessible in English regarding this scale. |  |
| Professional Quality of Life Scale version 5 (ProQOL-5) (Stamm, 2009). | The ProQOL-5 is a 30 item self-report measure of the positive and negative effects of working with people who have experienced extremely stressful events. It comprises of 3 subscales measuring Compassion Fatigue, Burnout and Compassion Satisfaction. CS alpha reliabilities = .87 BO alpha reliabilities = .72 CF alpha reliabilities = .80. Early information on test-retest data show good reliability. | 6 |
| Swedish version of the Shirom Melamed Burnout Questionnaire (SMBQ) (Grossi et al., 2003) | The SMBQ (originally by Melamed et al., 1999) is a 22-item self-report burnout measure consisting of 4 subscales: Physical Fatigue; Cognitive weariness; Tension and Listlessness. The Swedish version was shown to have consistencies (α>0.70) across the subscales, but a Rasch-analysis suggested omission of the Tension factor (Lundgren-Nilsson et al., 2012). | 1 |
| Compassion  Satisfaction/Fatigue Self-Test (CSFST) (Figley & Stamm, 1996). | The CSFT is a 66-item self-report measure comprised of three subscales: compassion fatigue, compassion satisfaction, and burnout. According to Stamm (2002), all 3 subscales have demonstrated good alpha reliability: α =.87 on the compassion fatigue subscale; α =.90 on the burnout subscale; and α =.87 for the compassion satisfaction subscale. | 3 |
| Well-being Index (WBI) (Dyrbye et al., 2014) | The WBI is a seven item self-report index, measuring domains of burnout, depression, stress, fatigue, and mental and physical quality of life. It consists of yes/no items and respondents receive a score from 0 to 7 based on responses. At a threshold score of ≥ 4, the PWBI’s sensitivity for identifying physicians with low mental quality of life is 73.3 % with a specificity of 81.0 % (Dyrbye et al. 2013). | 1 |
| Stanford Professional Fulfilment Index (PFI) (Trockel et al., 2018) | The PFI is a 16 item self-report measure assessing the degree of intrinsic positive reward the individual derives from his or her work, including happiness, meaningfulness, contribution, self-worth, satisfaction, and feeling in control when dealing with difficult problems at work. It consists of 3 scales- professional fulfillment; work exhaustion; and interpersonal disengagement and an overall burnout scale. Response options are on a five-point Likert scale (“not at all true” to “completely true” for professional fulfillment items and “not at all” to “extremely” for work exhaustion and interpersonal disengagement items). Each PFI item is scored from 0 to 4, using the associated five-point Likert scale. All scales have demonstrated good alpha reliability: α =.86 on the work exhaustion scale; α =.92 on the interpersonal disengagement scale; α =.91 on the professional fulfillment scale; α =.92 on the overall burnout scale (Trockel et al., 2018). | 1 |
| Stress Rating Form & Burnout Adjectives (described in Ray, 1981). | The stress rating form was devised for the study to collect pre- and post-ratings on participants by one close friend/relative and co-workers or supervisor. The observer was asked to rate the amount of stress that he/she believed that the subject was currently experiencing in five areas (home, job interpersonal relationships, health, and school), and to rate the amount of burnout the subject was feeling, on a 1 to 10 scale, with 1 being little stress and 10 being highly stressed. Additionally, the observer rated the subject on 16 adjectives on a 1 to 10-point scale, in order to compute an overall burnout score (the method for this is reportedly described in Thompson (1980) but this could not be accessed by reviewers). | 1 |

**S5:** **Table showing recommendations for future research**

| **Intervention** | **Recommendations/ Questions for future research** |  |
| --- | --- | --- |
| DBT skills training | - Larger and more diverse samples; control groups - Direct patient-related outcomes | |
|  | - Compare effectiveness of person-directed interventions, versus organizational interventions only, versus a combination. - Would longer training and supervision groups increase effectiveness? - Include clinician burnout as a variable within DBT effectiveness and efficacy studies - Account/control for different settings differing initial levels of clinician burnout - Account for variations in learning and utilization of training - Administer questionnaires to assess subjects' knowledge of and utilization of DBT. | |
| PSI training | - Focus on PSI competencies - Longer-term evaluation of implementation in practice - Outcome-based intervention trials - Larger samples, different settings, more rigorous methodologies | |
| ACT training | - Compare the impact of brief interventions based on ACT, psychoeducation, or an amalgam of both. - Improved rigour | |
| Supervision | - Larger samples, inclusion of different settings/specific roles of participants. - What structure of systematic clinical supervision is most effective? - Longer duration of study/supervision may be required to see changes in burnout - Incorporate qualitative and quantitative data to measure STS responses. - Can supervision serve as a preventative strategy for MHWs who do not yet have STS responses? | |
| EBS training/ prevention | - Larger samples, collect demographic data - Separate the various components of the intervention for comparison - Longer and more intensive STS education program - Follow up 2-3 months later, giving MHWs time to implement/ incorporate STS prevention plans - Exploring the effects of intermittent booster sessions of the intervention - More rigorous designs e.g. multiple baseline/ wait-list control groups, randomization - Measure primary traumatic stress and burnout - Participant interviews to distill the active elements of interventions | |
| Resiliency training | - More CF exploratory longitudinal studies with treatment and control groups - Additional CF research exploring CF over the course of clinicians’ careers - Comparing CF symptoms between CITs and practicing mental health professionals - Qualitative approaches exploring lived experiences with CF symptoms as well as during and after CF treatments to determine active ingredients for meaningful CF symptom reduction. | |
| Mindfulness/ compassion | - Are MBIs alone sufficient to generate self-compassion? - Increased rigour: large, controlled studies; random sampling across settings; long term follow up - Explore possible applications to other disciplines/ health workers - Explore mechanisms through which the Interpersonal Mindfulness Program works - Explore the adverse reactions reported in one study (Bartels-Velthuis et al., 2020). | |
| ‘BREATHE’ burnout reduction workshop | - Separate BREATHE content into smaller modules delivered over time - Trial additional electronic resources and reminders outside the workshop setting. - Revise BREATHE materials to specifically address how to cope with job demands that promote burnout. - Target interventions to MHWs experiencing higher levels of burnout. - Randomised control groups and longer follow-up periods - Ensure acceptability and feasibility with racial and ethnic minority groups | |
| Expressive arts | - Recruit participants from a variety of settings to examine individual differences | |
|  | - Use a wider variety of research methods to explore impact - Investigate the precise effects of group drumming on factors associated with burnout | |
| Peer support/ relationships | - Longitudinal designs with follow-up to explore longevity of effects. - Compare: online and face-to-face peer groups; hybrid models that include both online and face-to-face components. - Focus on both process and outcome measures - Control groups and larger samples. - Recruit participants with higher levels of emotional exhaustion to explore impact on this aspect of burnout more specifically | |
| Balint-like group | - Larger sample sizes with more statistical power | |
| Training in other therapeutic interventions (CBT;  psychosis/BPD interventions). | - More rigorous experimental designs | |
|  | - Does training result in improved patient outcomes? | |
|  | - Will results generalise to other mental health providers? - Do reductions in burnout for MHWs attending training sustain over time? | |
| Wellness/stress reduction | - Recruit homogeneous samples or else stratify more varied samples by job classification | |
|  | - Larger samples, randomization to groups | |
|  | - Explore differences between single and partnered MHWs, including experience and/or frequency of touch in their lives. | |
|  |  | |

*Note.* ACT = acceptance and commitment therapy; BREATHE = Burnout Reduction: Enhanced Awareness, Tools, Hand-outs, and Education; CF = compassion fatigue; CITs = counsellors in training; DBT= dialectical behaviour therapy; MBIs= mindfulness-based interventions; MHWs= mental health workers; PSI = psychosocial interventions; STS = secondary traumatic stress
