# Supplementary material for: Interventions to address empathy-based stress in mental health workers: A scoping review and research agenda
Source: PLoS One. 2024 Dec 5;19(12):e0306757. doi: 10.1371/journal.pone.0306757 (PMC11620613; doi:10.1371/journal.pone.0306757)
Supplement: Attachment — Submitted filename: PONE-D-23-33814-1 revised.pdf [file pone.0306757.s002.pdf]

PLOS ONE

Interventions to address empathy-based stress in mental health workers: A scoping review and research agenda

--Manuscript Draft--

**Manuscript Number:**	PONE-D-23-33814
**Article Type:**	Research Article
**Full Title:**	Interventions to address empathy-based stress in mental health workers: A scoping review and research agenda
**Short Title:**	Interventions for empathy based stress in mental health workers
**Corresponding Author:**	Hannah May<br>Avon and Wiltshire Mental Health Partnership NHS Trust<br>Bristol, UNITED KINGDOM
**Keywords:**	empathy based stress; compassion fatigue; Burnout; secondary traumatic stress; mental health workers; Scoping review; research agenda
**Abstract:**	Consistently engaging with client distress can negatively impact mental health workers (MHWs). Various terms are used to describe this phenomenon, which can all be captured by the concept of empathy-based stress (EBS). Previous reviews have not addressed MHWs as a distinct group, despite evidence suggesting they are particularly vulnerable to EBS. In the context of rising mental health needs triggered by the COVID-19 pandemic, it is especially important to understand how to mitigate the impact of EBS on MHWS. This review therefore aimed to identify and describe available interventions. A systematic scoping review of the literature concerning interventions for EBS in MHWs between 1970 and 2022 was undertaken using five electronic databases. 51 studies were identified, which varied significantly with regards to: interventions used; study methodology and theoretical underpinnings. Studies were grouped according to the level at which they aimed to intervene, namely: individual; team or organisational. Most studies intervened at the level of the individual, despite the proposed causes of EBS being predominantly organisational. Furthermore, theoretical links to the origins of EBS were largely unclear. A dedicated research agenda is set out to address these and other issues identified in the field and to guide future research.
**Order of Authors:**	Hannah May
	Josie Millar
	Emma Griffiths
	Chris Gillmore
	Michael West
	Mhairi Kristoffersen
	Ross Robinson
**Additional Information:**	
**Question**	**Response**
**Financial Disclosure**<br><br>Enter a financial disclosure statement that describes the sources of funding for the work included in this submission. Review the submission guidelines for detailed requirements. View published research articles from *PLOS ONE* for specific examples.	The author(s) received no specific funding for this work.

Powered by Editorial Manager® and ProduXion Manager® from Aries Systems Corporation

This statement is required for submission and **will appear in the published article** if the submission is accepted. Please make sure it is accurate.<br><br>**Funded studies**<br>Enter a statement with the following details:<br>• Initials of the authors who received each award<br>• Grant numbers awarded to each author<br>• The full name of each funder<br>• URL of each funder website<br>• Did the sponsors or funders play any role in the study design, data collection and analysis, decision to publish, or preparation of the manuscript?<br><br>Did you receive funding for this work?	
**Competing Interests**<br><br>Use the instructions below to enter a competing interest statement for this submission. On behalf of all authors, disclose any competing interests that could be perceived to bias this work—acknowledging all financial support and any other relevant financial or non-financial competing interests.<br><br>This statement is required for submission and **will appear in the published article** if the submission is accepted. Please make sure it is accurate and that any funding sources listed in your Funding Information later in the submission form are also declared in your Financial Disclosure statement.<br><br>View published research articles from *PLOS ONE* for specific examples.	The authors have declared that no competing interests exist.

**NO authors have competing interests**<br><br>Enter: *The authors have declared that no competing interests exist.*<br><br>**Authors with competing interests**<br><br>Enter competing interest details beginning with this statement:<br><br>*I have read the journal's policy and the authors of this manuscript have the following competing interests: [insert competing interests here]*<br><br>* typeset	
**Ethics Statement**<br><br>Enter an ethics statement for this submission. This statement is required if the study involved:<br><br>• Human participants<br>• Human specimens or tissue<br>• Vertebrate animals or cephalopods<br>• Vertebrate embryos or tissues<br>• Field research<br><br>Write "N/A" if the submission does not require an ethics statement.<br><br>General guidance is provided below. Consult the submission guidelines for detailed instructions. **Make sure that all information entered here is included in the Methods section of the manuscript.**	N/A

Powered by Editorial Manager® and ProduXion Manager® from Aries Systems Corporation

**Format for specific study types**<br><br>**Human Subject Research (involving human participants and/or tissue)**<br>• Give the name of the institutional review board or ethics committee that approved the study<br>• Include the approval number and/or a statement indicating approval of this research<br>• Indicate the form of consent obtained (written/oral) or the reason that consent was not obtained (e.g. the data were analyzed anonymously)<br><br>**Animal Research (involving vertebrate animals, embryos or tissues)**<br>• Provide the name of the Institutional Animal Care and Use Committee (IACUC) or other relevant ethics board that reviewed the study protocol, and indicate whether they approved this research or granted a formal waiver of ethical approval<br>• Include an approval number if one was obtained<br>• If the study involved *non-human primates*, add *additional details* about animal welfare and steps taken to ameliorate suffering<br>• If anesthesia, euthanasia, or any kind of animal sacrifice is part of the study, include briefly which substances and/or methods were applied<br><br>**Field Research**<br><br>Include the following details if this study involves the collection of plant, animal, or other materials from a natural setting:<br>• Field permit number<br>• Name of the institution or relevant body that granted permission	
**Data Availability**<br><br>Authors are required to make all data underlying the findings described fully available, without restriction, and from the time of publication. PLOS allows rare exceptions to address legal and ethical concerns. See the PLOS Data Policy and FAQ for detailed information.	Yes - all data are fully available without restriction

Powered by Editorial Manager® and ProduXion Manager® from Aries Systems Corporation

A Data Availability Statement describing where the data can be found is required at submission. Your answers to this question constitute the Data Availability Statement and **will be published in the article**, if accepted.<br><br>**Important:** Stating 'data available on request from the author' is not sufficient. If your data are only available upon request, select 'No' for the first question and explain your exceptional situation in the text box.<br><br>Do the authors confirm that all data underlying the findings described in their manuscript are fully available without restriction?	
**Describe where the data may be found in full sentences. If you are copying our sample text, replace any instances of XXX with the appropriate details.**<br><br>• If the data are **held or will be held in a public repository**, include URLs, accession numbers or DOIs. If this information will only be available after acceptance, indicate this by ticking the box below. For example: *All XXX files are available from the XXX database (accession number(s) XXX, XXX.).*<br>• If the data are all contained **within the manuscript and/or Supporting Information files**, enter the following: *All relevant data are within the manuscript and its Supporting Information files.*<br>• If neither of these applies but you are able to provide **details of access elsewhere**, with or without limitations, please do so. For example:<br><br>*Data cannot be shared publicly because of [XXX]. Data are available from the XXX Institutional Data Access / Ethics Committee (contact via XXX) for researchers who meet the criteria for access to confidential data.*<br><br>*The data underlying the results presented in the study are available from (include the name of the third party*	All relevant data are within the manuscript and its Supporting Information files.

and contact information or URL).<br>• This text is appropriate if the data are owned by a third party and authors do not have permission to share the data.<br><br>* typeset	
Additional data availability information:	

Powered by Editorial Manager® and ProduXion Manager® from Aries Systems Corporation

Manuscript

Click here to access/download;Manuscript;Manuscript.docx

Interventions to address empathy-based stress in mental health workers: A scoping review and research agenda

Hannah May[1,2*], Emma Griffith[1,2], Josie Millar[1], Chris Gillmore[2,] Mhairi Kristoffersen[1], Ross Robinson[2], Michael West[3]

[1] University of Bath, Department of Psychology, Bath, UK

[2]Avon and Wiltshire Mental Health Partnership NHS Trust, Bath, UK

[3] University of Lancaster, Management School, Lancaster, UK

*** Correspondence:**
Hannah May
hannahrebeccamay@hotmail.co.uk

Word count (excluding abstract, tables, references and appendices): 9698

Tables: 7 in body of text; 3 additional in appendices (all tables are found at the end of this draft)

Figures: 3

Keywords: empathy based stress 1, compassion fatigue 2, burnout3, secondary traumatic stress4, mental health workers5, scoping review6, research agenda7

Abstract

Consistently engaging with client distress can negatively impact mental health workers (MHWs). Various terms are used to describe this phenomenon, which can all be captured by the concept of empathy-based stress (EBS). Previous reviews have not addressed MHWs as a distinct group, despite evidence suggesting they are particularly vulnerable to EBS. In the context of rising mental health needs triggered by the COVID-19 pandemic, it is especially important to understand how to mitigate the impact of EBS on MHWS. This review therefore aimed to identify and describe available interventions. A systematic scoping review of the literature concerning interventions for EBS in MHWs between 1970 and 2022 was undertaken using five electronic databases. 51 studies were identified, which varied significantly with regards to: interventions used; study methodology and theoretical underpinnings. Studies were grouped according to the level at which they aimed to intervene, namely: individual; team or organisational. Most studies intervened at the level of the individual, despite the proposed causes of EBS being predominantly organisational. Furthermore, theoretical links to the origins of EBS were largely unclear. A dedicated research agenda is set out to address these and other issues identified in the field and to guide future research

Introduction

What is Empathy-based Stress?

Empathy is the interpersonal capacity to be sensitive to others and understand their feelings by actively entering into their experiences (Elliot et al., 2018). It is thought to be a key component of compassion (Atkins & Parker, 2012; Strauss et al., 2016), wherein engagement with the suffering of another facilitates actions to prevent and alleviate that suffering (Gilbert, 2014). People accessing mental health services are found to have better outcomes in terms of mental health recovery if they

This is a provisional file, not the final typeset article

experience compassionate (Spandler & Stickley, 2011) and empathic (Elliot et al., 2018) relationships with staff caring for them. This may be especially important in mental health services given the stigma that persists in both society and health services regarding mental distress (Ahmedani, 2011; Henderson et al., 2014). Clinicians also stand to benefit from such practice, with evidence highlighting that compassionate practice can protect against the harmful effects of empathic distress (Klimecki et al., 2014); lead to positive emotions (Klimecki et al., 2013); and reduce psychological distress (Kirby et al., 2017).

Despite the benefits associated with compassionate and empathic care, research has also identified potential costs to clinicians working with distressed clients. These include: burnout (Maslach, 1993); secondary traumatic stress (STS; Figley, 1995; Newell & MacNeil, 2010); compassion fatigue (CF; Figley, 1995); and vicarious trauma (VT; Pearlman & Saakvitne, 1995). Whilst distinct definitions exist for these terms (see Table 1), the extent to which they differ from one another is unclear and they are often used interchangeably in the literature (Turgoose & Maddox, 2017; Rauvola et al., 2019).

Table 1

Definitions of terms included in the Empathy-based stress construct

Term	Definition
**Burnout**<br>(Maslach, 1993).	A psychological response to chronic interpersonal stressors at work, resulting in exhaustion, detachment and feelings of incompetence. First identified in caregiving professions where services are provided via a relationship with people in need (Maslach 1982; 2006). These relationships (whilst rewarding) necessitate prolonged and intense emotional contact with clients which over time can produce burnout (Maslach, 2006).
**Secondary Traumatic Stress (STS)**	A form of second-hand post-traumatic stress disorder (PTSD). In STS, symptoms (e.g. hypervigilance, avoidance, intrusive

Term	Definition
(Figley, 1995; Newell & MacNeil, 2010).	thoughts/memories) arise out of exposure to the trauma of others, rather than through direct personal experience.
**Compassion Fatigue (CF)**<br><br>Figley (1995).	Figley (1995) substituted STS with the term CF as a less stigmatising way of referring to the impact of these experiences on healthcare professionals. CF is “a state of exhaustion and dysfunction biologically, psychologically, and socially as a result of prolonged exposure to compassion stress” (p. 253). CF has an inverse relationship with CS; leading to the suggestion that overwhelming levels of CF may interfere with the capacity to experience CS (Rossi et al., 2013; Stamm, 2002).
**Vicarious Trauma (VT)**<br><br>(Pearlman & Saakvitne, 1995).	VT is defined as “the transformation that occurs in the inner experience of the therapist [or worker] […] as a result of empathic engagement with clients’ trauma material” (Pearlman & Saakvitne, 1995, p. 31). Whilst VT may result in PTSD-type responses (flashbacks, intrusions, dissociation) (Beaton & Murphy, 1995), definitions emphasise the negative impact on the clinician’s cognitive schemas (Pearlman & Saakvitne, 1995).

Note. STS = secondary traumatic stress; CF= compassion fatigue; VT= vicarious trauma; PTSD= post-traumatic stress disorder

These inconsistencies led Rauvola et al. (2019) to propose an alternative conceptualisation in which VT, burnout, CF and STS are all highly related aspects of ‘empathy-based stress’ (EBS; Figure 1). EBS is described as applicable to anyone whose occupation exposes them to the distress of others and requires that they respond with engagement and empathy. It is defined as “a stressor–strain-based process of trauma at work, wherein exposure to secondary or indirect trauma, combined with empathic experience, results in empathy-based strain and additional outcomes (i.e., other occupational health/strain outcomes; work affect, behaviours, and cognitions)” (Rauvola et al., 2019, p299). In the EBS model; VT, STS and CF are the three varying forms of empathy-based strain that result from empathic engagement with the traumatic experiences of others. The state of burnout is

This is a provisional file, not the final typeset article

conceptualised as a reaction to this strain, though the authors acknowledge there are common features between all four constructs.

EBS is a recently conceptualised model which has not yet been applied within the wider literature; therefore, its feasibility and usefulness as a framework remain untested. However, EBS brings together established pre-existing concepts in a way that allows for distinct but inter-related terms to co-exist, thereby offering a potential solution to the conceptual ambiguities outlined above. Furthermore, it allows that anyone who empathically engages with second-hand distress in a work setting can consequently experience undesirable outcomes, which can interact with pre-existing individual factors to make EBS more or less likely/ severe. It is therefore dynamic, process-driven and applicable across professional fields. In this way it is akin to established models in the occupational health literature, including the Job Demands- Resources model which posits that all jobs comprise interacting sets of psychologically taxing demands and rewarding job resources (Bakker & Demerouti, 2007) . This review will henceforth use the term EBS, in reference to the dynamic model of "traumatic stressor exposure, empathic experience, and adverse reactions" (p. 297) described by Rauvola et al.

Why Prevent/Treat Empathy Based Stress in Mental Health Workers?

There is evidence to suggest that EBS impacts care providers from diverse professional backgrounds including nurses, physicians, social workers and mental health workers (Cavanagh et al., 2020; Rossi et al., 2013). Mental health workers (MHWs) however, are often required to engage explicitly with their clients' distressing experiences during care provision (Canfield, 2005). For example, successful psychotherapy is thought to depend in part on the clinician's ability to empathise with and to some degree absorb the pain that clients are experiencing (Rogers, 1967; Rothschild & Rand, 2006). Compared to GPs and non-medical personnel, MHWs have been found to show greater empathic

concern for others and higher discomfort at witnessing suffering (Santamaría-García et al., 2017). This empathic engagement predicts better client outcomes (Elliot et al., 2018); however, leaves MHWs at increased risk of EBS (Canfield, 2005; Jachens et al., 2018).

The rates of trauma experiences are found to be higher amongst people accessing mental health services, with estimates suggesting that roughly half have experienced physical abuse, whilst over a third have experienced sexual abuse (Mauritz et al., 2013). As services become increasingly aware of the importance of trauma-informed approaches to health and social care (Sweeney et al., 2016) MHWs are being encouraged to explicitly ask clients about potential traumatic experiences (Read et al., 2007). While undoubtedly a positive step, this seems likely to increase the quantity of traumatic material MHWs are exposed to. In their review, Turgoose and Maddox (2017) found that greater exposure to client trauma/distress was associated with higher CF in MHWs. EBS is associated with a range of adverse outcomes including poor job satisfaction, absenteeism and poor patient care (Figley 2002; Mathieu, 2007). It can lead to what Baranowsky (2002) termed ‘the silencing response’, whereby health workers begin to minimise, shutdown or divert away from traumatic material brought to them by the client.

Current Review and Aims

Previous reviews targeting healthcare workers suggest that intervention/training may be effective in reducing EBS (e.g. Cocker & Joss, 2016), however, existing reviews have either amalgamated MHWs with other healthcare workers as one group (e.g. Conversano et al., 2020) or excluded them (e.g. Cocker & Joss, 2016). Bercier and Maynard (2014) attempted to review EBS interventions for MHWs, however were unable to identify relevant studies and called for more research. Sutton et al (2022) examined the impact of organisational factors on some specific forms of EBS, however did

This is a provisional file, not the final typeset article

not consider other forms of intervention and looked only at particular professional groups working in mental health.

Prompted by these challenges, this paper aimed to review available interventions for preventing or treating EBS in MHWs specifically. Given the urgency indicated by staff stress levels (NHS, 2023) and workforce shortages it seems timely to ask; "What interventions exist to prevent or treat EBS in MHWs?". Using systematic scoping methodology, we aimed to answer this question via the following objectives:

1) To assess and summarise the available interventions for preventing or treating EBS in MHWs
2) To ascertain the theoretical underpinnings and assumptions of these interventions
3) To assess how EBS has been measured in these studies
4) To make recommendations for future research into the treatment and prevention of EBS in MHWs

Once the authors had conducted the initial review and assessed the nature of the available literature, a second aim was set: to provide a dedicated research agenda that summarises the existing state of the literature on interventions for EBS in MHWs and sets out key issues and directions for future research.

NHS England (2018) warned that the ever-growing need for healthcare services leaves NHS staff vulnerable to CF, which has been linked to depression, stress and chronic illness (Barr, 2017; Canfield, 2005; Demerouti, 2000; Mathieu, 2007). These are serious concerns for the workforce, as demonstrated in a recent NHS staff survey which found that 44.8% of workers felt unwell due to work-related stress; a percentage that has been climbing steadily since 2016 (NHS, 2023). This has led West and Chowla (2017) to call for a culture change in the NHS to enable senior staff to lead compassionately and sensitively rather than being solely driven by achieving targets and reducing

costs. However, Dean et al. (2019) highlight that such shifts require changes in the framework of our healthcare systems that will not be simple or quick to achieve.

Method

Scoping Review

This review employed a systematic scoping review method. Scoping reviews offer a means to map out the scope of the literature in a given area and to provide an overview of the type of evidence available (Mays et al., 2001). Whilst they may be a precursor to systematic reviews, they can also be conducted as self-contained reviews when there is insufficient knowledge about a subject to generate sufficiently specific questions for a systematic review(Armstrong et al., 2011).

To this end, scoping reviews: have broader inclusion criteria compared to systematic reviews; do not assess bias in included studies; do not attempt to synthesise evidence or arrive at precise recommendations. Instead, they aim to outline the research that is available, how it has been conducted and what theory/evidence has informed it. Despite these differences, scoping reviews are still systematic in nature and involve an a priori protocol and a methodical, exhaustive, and replicable search strategy (Arksey & O'Malley, 2005; Munn et al., 2018).

Munn et al (2018) provide guidance for authors choosing whether to conduct a systematic or scoping review. They give six indications for conducting scoping reviews, four of which are relevant to the current review: "To identify the types of available evidence in a given field; To clarify key concepts/ definitions in the literature; To examine how research is conducted on a certain topic or field…To identify and analyse knowledge gaps" (pp.2). The review was conducted in accordance with the Joanna Briggs Scoping Review Framework (Peters et al., 2017) and written up in line with the Prisma Extension for Scoping Reviews (Tricco et al., 2018). See Appendix A for a completed

This is a provisional file, not the final typeset article

Preferred Reporting Items for Systematic reviews and Meta-Analyses extension for Scoping Reviews (PRISMA-ScR) Checklist.

An a priori protocol was developed and registered on Open Science Framework: https://osf.io/b7kcr/

Electronic searches and Search Terms

Searches were conducted in the following databases: PubMed, PsychINFO (including APA PsycInfo, APA PsycArticles, APA PsycExtra and APA PsycTests), Embase, PTSDPubs. Grey literature was accessed via PsycExtra (theses) and the Cochrane Central Register of Controlled Trials (CENTRAL; unpublished trials). Studies available in English and conducted between 1970 (when the term burnout was introduced; Schaufeli et al., 2009) and 2021 were included in the initial search; in the updated search the included date range was 2021 to 2022.

The search terms were determined by considering the core elements of the research question, in consultation with a research librarian (see Table 2). Given the inconsistent/overlapping definitions of terminology noted in the literature (Turgoose & Maddox, 2017; Rauvola et al., 2019), EBS search terms incorporated the empathy-based strain constructs identified by Rauvola et al. (VT, STS, CF) as well as burnout (proposed to be an adverse reaction to empathy-based strain). Precise terminology, boolean operators and truncation were adapted/applied in accordance with each database (see Appendix B for full search strategy). When full texts could not be retrieved via inter-library loan, effort was made to locate/contact authors. Initial searches began in December 2020 and were completed in January 2021. An updated search was subsequently carried out in September 2022. All identified papers were uploaded to Covidence: https://app.covidence.org/reviews/130454

Table 2

Example of Search Terms

Search item of interest	Provisional search terms
Empathy based stress	Burnout OR Compassion fatigue OR secondary traumatic stress OR STS OR vicarious trauma OR VT OR compassion stress
Mental health workers	mental health nurs* OR forensic nurs* OR counsellor* OR psychologist* OR psychiatrist* OR psychotherapist* OR therapist* OR mental health support worker* OR mental health social worker* OR support time and recovery worker* OR family therapist* OR therapist* OR CBT therapist* OR mental health occupational therapist*
Intervention	Treat* OR prevent* OR train* OR program* OR interven*

Study selection

Titles and abstract screening as well as full-text review was conducted by two independent reviewers (HM and MK/RR) according to the inclusion/exclusion criteria shown in Table 3. Whilst the mental health worker search terms in Table 2 were used to guide the searches, some identified studies used different terms to describe participant job roles, or else more general terms e.g. doctor, nurse. In these cases the studies were carefully read in order to identify the nature of the client group participants were working with, and/or the study setting. Such studies were included only if they clearly referenced client groups with mental health needs e.g. clients with psychosis, or healthcare settings that clearly had a mental health focus e.g. psychiatric wards.

This is a provisional file, not the final typeset article

Table 3

Criteria for study inclusion/exclusion

	Inclusion Criteria	Exclusion Criteria
Population	At least 50% of participants are mental health workers (see Table 2 for included professions/synonyms).	Studies including other healthcare staff whose role does not have a mental health focus/the general public will be excluded.
		Children, adolescents
Concept	Studies involving any intervention (including organisational strategies e.g. increased supervision) or training program that aims to prevent or reduce EBS (including STS, CF, VT & burnout).	Studies that only assess MHW's individual differences and/or self-care strategies in relation to EBS.
	Studies that in some way evaluate the impact of the intervention/training on EBS.	
Context	Study settings may include any setting where mental health workers are employed.	
Source	Primary research studies, systematic reviews and meta-analyses of any publication status.	Theoretical papers, opinion pieces or letters to the editor
	Qualitative or quantitative studies.	Books, book chapters

Note. EBS = empathy-based stress; MHWs = mental health workers; STS = secondary traumatic stress; CF = compassion fatigure; VT = vicarious trauma.

Charting the Data

Information from included studies was charted by reviewers HM and MK, using the following headings:

Characteristics of Included Studies

Study characteristics. Authors, year of publication, study location, methodology, study design, study aims, EBS construct targeted, type of intervention/training, method of evaluating EBS.

Sample characteristics. Sample size, age, gender, setting, % of MHWs in sample, specific profession/role of MHWs.

Characteristics of Intervention/Training

This included: intervention/training type; the aims and rationales for the interventions/training programs administered; duration of intervention; and method of delivery. Information about the theoretical underpinnings of the intervention/training provided were recorded.

Outcomes

Measures used to assess EBS and, where possible, outcomes on these measures were recorded. Any recommendations for practice or further research on training/interventions of this nature were documented.

Results

Searches of all sources retrieved 20,552 records. After duplicates (*n* = 10964) and other ineligible sources (see Figure 2) were removed, 9,590 titles and abstracts were screened of which 9,436 were excluded. Of the 228 full text records screened, 177 were excluded. Therefore, 51 studies that met eligibility criteria (Figure 2) were included in the review. In this section, the characteristics of the included studies and their participants are first described. Thereafter, the EBS interventions used in the

This is a provisional file, not the final typeset article

studies are summarised with reference to their: nature; length; theoretical underpinnings and assumptions and; means of measurement. Though limited by considerable heterogeneity in the identified studies, the authors also attempt to place the findings in the context of relevant occupational health research by considering how they map on to the three types of organisational intervention for improving staff wellbeing and mental health described by Tetrick and Quick (2011).

Figure 2

PRISMA Flow Chart of the Screening Process

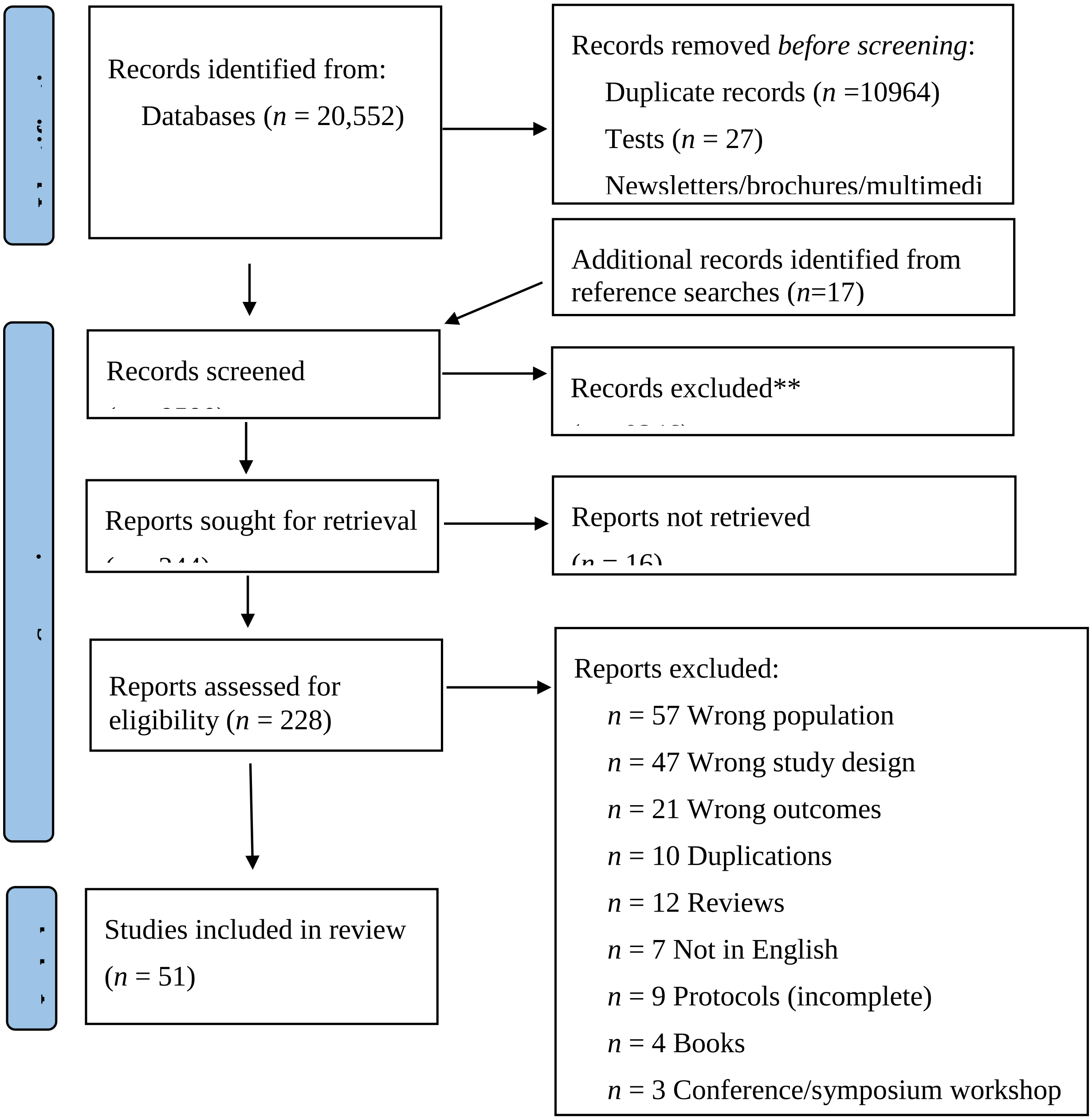

Characteristics of Included Studies

Table 4 provides an overview of the key features of the 51 included studies. The year of publication ranged from 1981 to 2021. The majority of studies (*n* = 28) were conducted in the United States of America (USA). Most studies (*n* = 43) used a quantitative methodology, but qualitative (*n* = 2) and mixed methods (*n* = 6) were also used. Study designs varied, ranging from case studies (*n* = 1) to randomised controlled trials (RCTs) (*n* = 5) with repeated measures designs (*n* = 25) being the most common. Samples varied in size from *N* = 2 to *N* = 296 and consisted of a diverse range of MHWs, most commonly nurses who comprised 100% of the sample in nine studies, and part of the sample in eight. Fourteen studies had sample populations consisting of multiple types of MHWs. Of the 40 studies that provided gender breakdowns: 27 comprised over 50% women; and seven were 100% women. Only 19 studies provided information on participant ethnicity (see Table 5), most of which had majority (*n* = 14) or entirely (*n* = 1) White samples. Other ethnicities represented included Black, Hispanic, Latino/Latina, Asian, Indian, Chinese, Malay, Mauritian, multi-racial, American Indian and Pacific Islander. Eight of the 20 studies reporting ethnicity described a proportion of participants as having ‘other’ or ‘non-White’ ethnicity, with no further information provided. A further four studies reported a percentage of White participants but provided no indication of the ethnic backgrounds of the rest of the sample. Settings included: community mental health (*n* = 14); inpatient (*n* = 7); outpatient/clinic (*n* = 4); multiple settings (*n* = 3); forensic (*n* = 3); mental health centres (*n* = 2); substance abuse services (*n* = 2); veteran’s services (*n* = 1); primary school (*n* = 1); domestic abuse shelter (*n* = 1); child advocacy centre (*n* = 1). Most studies targeted a single EBS aspect of EBS (*n* = 47), whilst some targeted multiple (*n* = 4). Burnout was by far the most common outcome targeted targeted (*n* = 41) although STS (*n* = 7) and CF (*n* = 8) were also addressed. According to the EBS model (Rauvola et al., 2019), this means that over 80% of identified studies were targeting one of the adverse outcomes of empathy-based strain, rather than addressing empathy-based strain itself. Most

This is a provisional file, not the final typeset article

studies (n = 46) were classified as treatment studies, in that, as far as could be determined, they aimed to examine the ability of an intervention to treat/impact upon EBS, with the assumption apparently being that EBS was already present. Five studies aimed to both treat and prevent EBS. No studies targeted prevention alone.

Table 4

Characteristics of Included Studies

ID	Study	Country	Publication Status	Study Design	EBS Construct Targeted	Setting	N	% of MHWs (specific role)	Gender	Intervention
**01**	Haynos et al., 2016	USA	PRJ	Quantitative<br>Repeated measures	Burnout	CYP inpatient	22	100% (MH nurses)	Not Given	DBT skills coaching training
**02**	Roberts, 2018	UK	UT/D	Quantitative<br>Repeated measures	Burnout	Not given	141<br>(39 at FU)	Not given	79.4% women	DBT skills training
**03**	Clarke et al., 2015	UK	PRJ	Quantitative<br>RCT	Burnout	Inpatient, outpatient	140 (57 at FU 1; 36 FU 2).	100%<br>(Staff working with clients with BPD)	Not given	ACT Training vs psycho-ed. BPD training[1]
**04**	Doyle et al., 2007	UK	PRJ	Quantitative<br>Experimental design	Burnout	Forensic	26	61.50%<br>(Forensic nurses)	73.1% women	Psychosocial intervention training
**05**	Hallberg, 1994	Sweden	PRJ	Mixed methods<br>Repeated measures	Burnout	CYP inpatient	11	100%<br>(Child MH nurses)	63.3% women	Systematic clinical supervision

[1] The primary aim of this RCT was to compare the effects of these two interventions on stigma towards clients with BPD; however clinician burnout was also measured

ID	Study	Country	Publication Status	Study Design	EBS Construct Targeted	Setting	N	% of MHWs<br><br>(specific role)	Gender	Intervention
**06**	Hunnicutt & MacMillan,<br><br>1983	USA	PRJ	Quantitative<br><br>Experimental design (2 intervention groups; 1 control)	Burnout	Community	251	Not given	Not given	Burnout workshop standalone vs with staff consultation.
**07**	Landis, 2010	USA	UT/D	Mixed methods<br><br>Repeated measures	ST	Community	5	100%<br><br>(SWs)	80% women	CF/ST workshop; 'Sharevision' meetings
**08**	Perseius et al., 2007	Sweden	PRJ	Mixed methods<br><br>Repeated measures	Burnout	Adult & CYP psychiatry clinics	22	100%<br><br>(Drs; psychol; MH nurses/care assistants; therapist, OT)	86.4% women	DBT training
**09**	Raab et al., 2015	Canada	PRJ	Quantitative<br><br>Repeated measures	Burnout	Community	22	100 %<br><br>(MH care workers)	100% women	MBSR
**10**	Razzaque & Wood, 2015	UK	PRJ	Mixed methods<br><br>Repeated measures	Burnout	Community	26	100%<br><br>(Psychr)	65.4% women	Mindfulness Retreat

ID	Study	Country	Publication Status	Study Design	EBS Construct Targeted	Setting	N	% of MHWs (specific role)	Gender	Intervention
**11**	Rollins et al., 2016	USA	PRJ	Quantitative<br>Experimental design	Burnout	Inpatient & outpatient, (mostly veterans services)	145	100%<br>(Behavioural health providers)	71% women	'BREATHE' burnout reduction workshop
**12**	Melchior et al., 1996	The Netherlands	PRJ	Quantitative<br>Experimental design	Burnout	Inpatient	161	100%<br>(MH nurses)	72% women	Primary nursing
**13**	Duckworth, 2012	USA	UT/D	Quantitative<br>Repeated measures	STS	CYP community	16	100%<br>(psychol; nurses; SWs, therapists; admin)	93.8% women	STS education program and prevention plan
**14**	Boone, 2006	USA	UT/D	Quantitative<br>Experimental design	STS	Not given	97 (53 at FU)	100%<br>(Domestic violence counsellors)	88.7% women	Poetry writing therapy
**15**	Flarity et al., 2016	USA	PRJ	Quantitative<br>Repeated measures	CF	Inpatient (emergency department)	7	100 %<br>(Forensic nurses)	100% women	CF prevention and resilience training
**16**	Suyi et al., 2017	Singapore	PRJ	Quantitative	Burnout	Not given	37	100%	81.1% women	MBSR

ID	Study	Country	Publication Status	Study Design	EBS Construct Targeted	Setting	N	% of MHWs (specific role)	Gender	Intervention
				Repeated measures				(Psychol; Psychr; SWs)		
**17**	Kovač et al., 2016	Slovenia	PRJ	Quantitative<br>Experimental design	Burnout	School	30	100%<br>(School counsellors)	100% women	Supervision (relational family model)
**18**	Van Kirk,<br>2020	USA	PRJ	Quantitative<br>Repeated measures	Burnout, STS & CS	Veteran MH services	57	100%<br>(nurses, SWs, psychol, psychr, counsellors, SA therapists; OTs; assistants)	64% women	Employee wellness program
**19**	Alenezi et al., 2019	Saudi Arabia	PRJ	Quantitative<br>Experimental design	Burnout	Inpatient	296	100%<br>(MH nurses)	49.3% women	Burnout prevention programme
**20**	Eriksson et al., 2018	Sweden	PRJ	Quantitative<br>RCT	Burnout	Not given	101 (81 at FU)	100%<br>(Psychol)	96% women (plus one NB person)	Web-based compassion program
**21**	Wymer, 2009	USA	UT/D	Quantitative	STS	Child advocacy centres	3	100%	100% women	Affective check-in supervision

ID	Study	Country	Publication Status	Study Design	EBS Construct Targeted	Setting	N	% of MHWs<br><br>(specific role)	Gender	Intervention
				Single case research design				(CSA counsellors)		
**22**	Chilton et al., 2020	USA	PRJ	Qualitative<br><br>Case study	CF & STS	Addiction treatment centre.	2	100%<br><br>(art/expressive arts therapists)	100% women	El Duende 'Process Painting'
**23**	Askey-Jones, 2018	UK	PRJ	Quantitative<br><br>Repeated measures	Burnout	Primary & secondary	69 (43 at FU)	52%<br><br>(MH Nurses)	76% women	MBCT group therapy
**24**	Ewers et al., 2001	UK	PRJ	Quantitative<br><br>Experimental design	Burnout	secure forensic settings	33 (20 at FU)	100%<br><br>(MH nurses)	51.5 % women	Psychosocial Intervention Training
**25**	Finamore et al., 2020	UK	PRJ	Quantitative<br><br>Repeated measures	Burnout	Not given	253 (201 at FU)	100%<br><br>Mixed	Not given	Knowledge & Understanding Framework PD Training
**26**	Reyes Ortega et al., 2019	Mexico	PRJ	Quantitative<br><br>Experimental design	Burnout	BPD Clinic	6	100%<br><br>(Psychol; psychr)	50% women	'Helping the Helper' social connectedness intervention
**27**	Brady et al., 2011	USA	PRJ	Quantitative	Burnout	Inpatient	16	100% (Psychr; Psychol; MH technicians	Not given	MBSR

This is a provisional file, not the final typeset article

ID	Study	Country	Publication Status	Study Design	EBS Construct Targeted	Setting	N	% of MHWs (specific role)	Gender	Intervention
				Repeated measures				Activity Therapists)		
**28**	Walker, 2018	USA	UT/D	Quantitative<br><br>Repeated measures	Burnout	Can't access-preview	34	100%<br><br>(Can't access-preview)	Can't access-preview	MBSR
**29**	Kiley et al., 2018	USA	PRJ	Quantitative<br><br>RCT	CF	Community	69 (56 at FU)	100<br><br>(SWs; counsellors; psychr; CMs, support staff and management)	Not given	Guided Imagery
**30**	Riley et al., 2017	USA	PRJ	Quantitative<br><br>2 experimental conditions, no control	STS, CF & Burnout	Multisite MH centre	38 (28 at FU 1; 25 at FU 2; 19 at 6 month FU)	100%<br><br>(Not given)	84.2% women	Yoga-based stress management vs cognitive-behavioural stress management
**31**	Rosada et al., 2015	USA	PRJ	Quantitative<br><br>Repeated measures cross-over design	Burnout	Community	45	100 %<br><br>(MH clinicians)	73.3% women	Reiki (reiki vs ‘sham’)

ID	Study	Country	Publication Status	Study Design	EBS Construct Targeted	Setting	N	% of MHWs (specific role)	Gender	Intervention
**32**	Ifrach & Miller, 2016	USA	PRJ	Quantitative<br><br>Repeated measures	CF	Domestic abuse shelters	30	100%<br><br>(DV counsellors)	100% women	Social action art therapy
**33**	Carmel et al.,<br><br>2014	USA	PRJ	Quantitative<br><br>Repeated measures	Burnout	Community	9	100% (MH<br><br>practitioners; SA counsellors)	88% women	DBT training
**34**	Newman et al.,<br><br>2015	South Africa	PRJ	Qualitative<br><br>Retrospective	Burnout	CYP Community	30	100% (Child Psychr, psychr, Drs, nurses, clinical psychol, OTS, admins, interpreter)	70.6% women	Drumming
**35**	Paulson et al.,<br><br>2015	USA	PRJ	Mixed methods<br><br>Repeated measures	Burnout	Community (rural)	6	100%<br><br>(psychol; counsellors)	Not given	Online peer consultation network (support group)
**36**	Luoma & Vilardaga,<br><br>2013	USA	PRJ	Quantitative<br><br>Experimental design	Burnout	Community	20	80<br><br>(Psychol; Counsellors)	40% women	ACT Training
**37**	Little,<br><br>2000	USA	UT/D	Quantitative	Burnout	Not given	37	100%	75.7% women	DBT training

This is a provisional file, not the final typeset article

ID	Study	Country	Publication Status	Study Design	EBS Construct Targeted	Setting	N	% of MHWs<br><br>(specific role)	Gender	Intervention
				Repeated measures				(Therapists; CMs; admins)		
**38**	Scarnera et al., 2006	Italy	PRJ	Quantitative<br><br>Repeated measures pilot study	Burnout	Not given	25	100%<br><br>(Nurses, educators; psychol; psychr; managers)	44% women	Interpersonal relationship management training
**39**	Ray,<br><br>1981	USA	UT/D	Quantitative<br><br>Experimental design	Burnout	Community	36	100%<br><br>(SWs; psychol; therapists; attendant; nurses; admin)	75% women	Stress management training program
**40**	Jones,<br><br>2009	UK	PRJ	Quantitative<br><br>Repeated measures	Burnout	Inpatient	72 (62 at 3rd FU, 49 at 4th FU)	100%<br><br>(MH nurses; HCAs; OTs<br><br>ward managers; CNSs)	57% women	Interventions for psychosis training + clinical practice development
**41**	Redhead et al.,<br><br>2011	UK	PRJ	Quantitative<br><br>RCT	Burnout	Forensic inpatient	42	100<br><br>(Forensic nurses)	Not specified	Psychosocial intervention training
**42**	Salyers et al.,	USA	PRJ	Quantitative	Burnout	Substance abuse	84 (74 at FU)	Not given	87% women	BREATHE Burnout

ID	Study	Country	Publication Status	Study Design	EBS Construct Targeted	Setting	N	% of MHWs (specific role)	Gender	Intervention
	2011			Experimental design						reduction retreat
**43**	Weingardt et al., 2009	USA	PRJ	Quantitative<br>RCT (2 experimental conditions, no control)	Burnout	Outpatient & controlled settings	147	100%<br>(SA counsellors)	62.1% women	Online CBT training + supervision meetings (high vs low fidelity)
**44**	Leykin et al., 2011	USA	PRJ	Quantitative<br>2 experimental conditions, no control	Burnout	Community	149 (112 at 1st FU 81 at 2nd FU)	100%<br>(SA counsellors)	62.6% women	Online training in CBT for substance abuse + supervision meetings (high vs low fidelity)
**45**	Gentry et al., 2004	USA	PRJ	Quantitative<br>Repeated measures	Burnout & CF	Not given	83	100%<br>(SWs; counsellors; psychols)	Not given	Certified CF Specialist Training
**46**	Hayes et al., 2004	USA	PRJ	Quantitative<br>Experimental design (2 experimental conditions; 1 control)	Burnout	Not given	93	100<br>(SA counsellors)	63% women	ACT training vs Multicultural training

ID	Study	Country	Publication Status	Study Design	EBS Construct Targeted	Setting	N	% of MHWs<br>(specific role)	Gender	Intervention
**47**	Anderson,<br>1982	USA	UT/D	Quantitative<br>Experimental design	Burnout	MH centre	40	100%<br>(counsellor-therapists)	60% women	Facilitator-led peer groups
**48**	Mehr et al.,<br>1994	USA	PRJ	Quantitative<br>Repeated measures	Burnout	Community	27	100%<br>('MH workers')	100% women	Stress-reduction/positive imagery conference + follow up meetings
**49**	Ballew 2020	USA	UT/D	Quantitative<br>Quasi-experimental control time series design	CF	Not specified	43 (19 at FU)	68% (‘MH professionals’)	76.9% women (2 people identified with multiple genders)	Professional Resilience and Optimization workshop
**50**	Chochol et al., 2021	USA	PRJ	Mixed methods<br>Repeated measures	Burnout	Child and adolescent psychiatry	6 (pilot study)	100% (Child and adolescent psychiatry fellows)	Not specified	Balint-like group incorporating brief emotional awareness modules

ID	Study	Country	Publication Status	Study Design	EBS Construct Targeted	Setting	N	% of MHWs (specific role)	Gender	Intervention
**51**	Bartels-Velthuis et al., 2020	Netherlands	PRJ	Quantitative Experimental design	CF	Outpatient MH clinic	47	100% (doctors; psychols; nurses; mindfulness teacher; SW; MH counsellor; physiotherapist; drama therapist)	91.5% women	Interpersonal Mindfulness Program + 45–60 min daily home practice

Note: ACT = acceptance and commitment therapy; BPD = borderline personality disorder; CF = compassion fatigue; CM = case managers; CNS = clinical nurse specialist; CS = compassion satisfaction; CSA = child sexual abuse; CYP= children and young people; DBT = dialectical behaviour therapy; FU = follow up; HCA= health care assistants; PD= personality disorder; PRJ= peer reviewed journal; MBCT = mindfulness-based cognitive therapy; MBSR = mindfulness-based stress reduction; MH = mental health; MSc= masters NB= non-binary; OT= occupational therapist; PD = personality disorder; psychr = psychiatrist; psychol = psychologist; RCT = randomised controlled trial; SA = substance abuse; ST= secondary trauma; STS = secondary traumatic stress; SW = social worker; UM/T= unpublished masters/thesis

Table 5

Studies reporting participant ethnicity

Study	Ethnicity of Participants
Razzaque & Wood, 2015	White: 18(69 %)<br>Black: 2(8 %)<br>Asian: 3(12 %)<br>‘Other’ ethnic backgrounds: 2(8 %).
Rollins et al., 2016	White: 111(77%)
Duckworth McNeil, 2012	African American: 8<br>Caucasian: 7<br>‘Other’ ethnic backgrounds: 1
Boone, 2006	White; 44 (83%)<br>Black: 4 (7.5%)<br>Hispanic: 5(9.4%)
Suyi, Meredith & Khan, 2017	Chinese: 29 (78.4%)<br>Malay: 2 (5.4%)<br>Indian: 4 (10.8%)
Wymer 2009	African American/Black: 1(33.3%)<br>Hispanic: 1(33.3%)<br>White: 1(33.3%)
Chilton et al., 2020	100% white
Askey-Jones, 2018	95% White British (no information on other 5%)
Kiley et al., 2018	African American: 18(26.1%)<br>White: 43 (62.3%)<br>Multiracial: 3 (4.4%)<br>‘Other’ ethnic backgrounds: 1(1.4%)<br>Missing data: 4 (5.8%)
Riley et al., 2017	94.7% White (no information on other 5.3%)
Rosada et al., 2015	White: 30 (66.7%)<br>'Non-white': 15 (33.3%)
Ifrach & Miller, 2016	Caucasian/White: 21(70%)<br>Hispanic/Latina: 3(10%)<br>African American/Black: 1(3.3%)<br>Mixed race: 1(3.3%)<br>‘Other’ ethnic backgrounds: 4(13.3%)

Study	Ethnicity of Participants
Luoma & Vilardaga, 2013	White: 19(95%)<br>Hispanic/Latino: 1(5%)
Jones, 2009	Black African: 55 %<br>White British: 16 %<br>African Caribbean: 9%<br>Black British: 8%<br>Mauritian: 7%<br>Indian: 3%<br>White African: 2%
Salyers et al., 2011	White: 62(79%)<br>Black:15(19%)<br>‘Other’ ethnic backgrounds: 2(3%).<br>Hispanic: 3(4%)
Weingardt et al., 2009	African American: 29 (19.7%)<br>Asian/Pacific Islander: 1 (0.7%)<br>White: 98 (65.3%)<br>Hispanic/Latino: 12 (8.2%)<br>‘Other’ ethnic backgrounds: 9 (6.1%)
Leykin et al., 2011	Not given; however, sample reportedly the same as Weingardt et al. (2009), plus two further participants
Hayes et al., 2004	White: 84%
Mehr et al., 1994	Asian American: 14.7%<br>Latina: 18.5%<br>White: 66.7%
Ballew, 2020	Caucasian: 35 (87.5%)<br>Multiracial: 3 (7.5%)<br>American Indian: 1 (2.5%)<br>Other: 1 (2.5%)

Interventions

Studies were included if they evaluated an intervention for reducing/preventing EBS that was externally facilitated (i.e. the organisation/researchers provide and arranged it). Studies measuring internally facilitated or ‘self-care’ behaviours (or other individual attributes) were excluded on the basis that it is primarily an

This is a provisional file, not the final typeset article

organisational responsibility, not an individual one, to tackle EBS. Two included studies challenged these criteria. Van Kirk's (2020) employee wellness program was organised and coordinated by the researchers, however relied on employees to deliver the wellness interventions (a different one for every week of the program) and took place during employee's lunch hour. Kiley et al.'s (2018) guided imagery study provided participants with guided imagery tracks on MP3 players and instructed them to listen to them three times a week over the intervention period, during work breaks. These studies were included because of the external facilitation by researchers in guiding participants on what, when and how to perform the interventions. Similar studies that provided a potential moderator of EBS without this structure were excluded, e.g., case study by Callander et al. (2019) who gave the participant a mindfulness app but no guidance on when and how to use this. However, it is important to draw a distinction between interventions that are externally facilitated (but still focus on change at the individual level) and those that are truly organisational (i.e., involving true system change).

Given the diverse range of interventions described, for clarity, they have been grouped according to the main intervention components as described in the studies themselves (Table 6). It is acknowledged some of the more integrative interventions contained various elements and could potentially be categorised multiple ways.

The length of the interventions varied considerably. The majority (n = 36) were delivered over weeks/months, with timeframes ranging from two weeks to 12 months. Some studies were delivered in workshops/retreats of one (n = 3), two (n = 6) and three (n = 1) days in duration, with one described as two to three days long. Three interventions spanned less than a day. For studies where the total hours of intervention received could be calculated, this ranged from two to 104 hours. Six studies required 'homework' activities in-between/in addition to attending the interventions, including meditation practice and unfacilitated peer groups.

Table 6

Descriptions of the broad categories of interventions used in identified studies

Intervention	Description
**Training in a therapeutic modality (*N* = 11)**	The most common training was Dialectical Behaviour Therapy (DBT; *n* = 5), followed by Psychosocial Intervention Training (PIT; *n* = 3); Cognitive Behavioural Therapy (CBT; *n* = 2) and specific training on understanding and working with psychosis (*n* = 1) and the knowledge and understanding framework (KUF) for working with borderline personality disorder (BPD; *n* = 1; this was compared with an ACT intervention- see below).
**EBS training and prevention/ Resiliency training (*N* = 7)**	Participants were educated on causes, signs and impacts of EBS and encouraged to develop coping/prevention strategies. Three studies included opportunities for participants to practice some of these strategies e.g. breathing exercises; progressive muscle relaxation; developing secondary trauma narratives. The study delivering resiliency training incorporated EBS education and development of coping strategies, as well as teaching resiliency skills (e.g. self-regulation, self-care) described as 'antibodies' that could bolster participants' ability to fight off compassion fatigue (Ballew, 2020). One study involved training MHWs to deliver the certified compassion fatigue specialist training (CCFST; Gentry & Baranowsky, 2004) to other MHWs experiencing CF (given the experiential elements of the training, it was hypothesised there would be a 'training-as-treatment' impact on participants' own CF).
**Mindfulness (*N* = 7)**	Studies using mindfulness-based stress reduction (MBSR; *n* = 4); group mindfulness-based cognitive therapy (MBCT; *n* = 1) and a mindfulness-based professional development (MBPD; *n* = 1) retreat taught participants mindfulness theory and practice and encouraged workers to develop their own practices.
**Wellness/stress reduction (*N* = 6)**	Two studies involved training in stress management techniques, including identifying stressors and practicing stress-reduction skills, including guided imagery and progressive muscle relaxation. Additional wellness interventions included guided imagery (*n* = 1); reiki (*n* = 1) and a wellness program (*n* = 1) incorporating multiple facets of wellness (e.g. mindfulness, nutrition and movement). One study compared yoga-based stress management (YBSM; combining yoga and aspects of cognitive stress management) with cognitive-behavioural stress management (CBSM; Antoni et al., 2006).

This is a provisional file, not the final typeset article

Intervention	Description
**Peer support/ relationships (*N* = 4)**	Two studies involved the facilitation of regular peer groups: one focused on sharing thoughts and emotions regarding burnout issues; the other primarily on case consultation. One study used a functional psychotherapeutic approach to foster emotional closeness between MHWs and one taught participants techniques for handling professional interpersonal relationships.
**Expressive arts (*N* = 4)**	Interventions included art therapy (*n* = 1); art therapy supervision (*n*= 1); poetry therapy (*n* = 1) and drumming (*n* = 1). Art therapy supervision was included here (rather than in supervision, below) because creating art was the central component of the intervention.
**Supervision** **(*N* = 3)**	Studies included psychodynamic group supervision (*n* = 1); relational family supervision (*n* = 1) and affective check in supervision (*n* = 1). It should be noted that whilst two studies introduced supervision to MHWs who had not previously been receiving it, the affective check in study recruited participants already receiving regular supervision and trained their supervisors to use the affective check in technique.
**Acceptance and Commitment Therapy** **(ACT; *N* = 3)**	These studies taught MHWs ACT skills for personal use. Two studies compared ACT with other interventions including BPD training (see above) and multicultural training.
**BREATHE (*N* = 2)**	The BREATHE (Burnout Reduction: Enhanced Awareness, Tools, Hand-outs, and Education) program (Salyers et al., 2011) incorporates burnout prevention information with practices including mindfulness, social (e.g. support structures and boundaries), physical (e.g. body scan), cognitive (e.g. thought challenging; identifying values), imagery, and other self-care activities.
**Primary nursing** **(*N* = 1)**	A nursing intervention whereby both psychiatric and practical nurses were assigned to patients as primary nurse caregivers.
**Balint-like group (*N*=1)**	One group member volunteered to share a challenging case, after which group attendees asked clarifying questions. For approximately 10 min, the other group members and the group facilitators processed the case and their emotional responses. Afterwards, the presenter returned to the group and had the opportunity to respond to the group's commentary, stimulating further discussion until the end of the hour.

Intervention	Description
**Compassion enhancement program** **(*N* = 1)**	The program involves standard mindfulness exercises such as breathing anchor and body scans, and compassion-focused exercises such as loving-kindness, and exercises of compassion with self and others.

Note: ACT = acceptance and commitment therapy; BPD = borderline personality disorder; MHWs = mental health workers.

Theoretical Underpinnings and Assumptions

There are at least two levels on which to consider the theoretical rationale for the interventions used by included studies to address EBS in MHWs. Firstly, there is the route via which studies have attempted to intervene and what this reveals about their underlying assumptions as to the cause and nature of EBS. Secondly, there are the theoretical frameworks/rationales underpinning some of the specific interventions used.

Route of Intervention

We have described the routes of intervention taken by the included studies as as: 1. individual (personal practices; awareness-raising and role training); 2. team and 3. organisational.

Individual. Individual interventions intervened at the level of the individual to help them manage the impact of EBS and/or other demands associated with work. There were three subtypes of individual intervention: Personal resilience; awareness-raising and role-training.

Personal resilience. These interventions sought to provide MHWs with treatment and/or train them in using personal practices to manage their own negative experiences. These included: Wellness/stress reduction; mindfulness; ACT; compassion enhancement; expressive arts and aspects of the BREATHE intervention and resiliency workshops.

This is a provisional file, not the final typeset article

Awareness-raising. Awareness-raising interventions were those that attempted to alleviate EBS by making MHWs aware of and prepared to tackle the negative effects of EBS. These included: EBS prevention and aspects of the BREATHE intervention and resiliency workshops.

Role-training. Role-training interventions were those that provided MHWs with training in therapeutic modalities or approaches to use with their clients. These included: training in a therapeutic modality.

Team. Team interventions were those focussed on team relationships and supportiveness. These included the interventions: supervision; peer groups/relationships and Balint-like groups.

Organisational. Organisational interventions are those in which changes to the structure/running of the organisations employing MHWs were made. These included: primary nursing.

Figure 3 displays how these levels of intervention intersect with the three types of organisational intervention for improving staff wellbeing and mental health described by Tetrick and Quick (2011): i) Primary interventions aim to decrease or remove stressors at the organisational level i.e. at the source. They require changes to workplace practice and usually involve employees in the processing of developing interventions ; ii) secondary interventions attempt to alter the individual's perception of, or responses to a stressor. They enable work-related stressors to be swiftly identified and attempt to mitigate these by increasing employees' coping skills, awareness and knowledge e.g. through additional training; iii) tertiary interventions are reactive and focus on rehabilitation of those already experiencing significant strain resulting from stressors e.g. psychological therapy or occupational health services (Tetrick and Quick, 2011). This demonstrates that all but one of the interventions were of the secondary type although they were operating at various levels of individual and team intervention.

Specific Theories and Rationale

Reference to theory was inconsistent, with some studies referring to an evidence base rather than theoretical concepts. Where theoretical underpinnings for interventions were made explicit, these are highlighted below (grouped by type of intervention). Evidence base/rational given for interventions not explicitly linked to theory are summarised in Table 7.

Psychosocial Intervention Training (PIT). Clients with serious mental health difficulties may be perceived as troublesome, hard to understand and difficult to help and these perceptions may make MHWs feel demotivated (Jones & Lowe, 2001) and burnt out (Savicki & Cooley,1987; Moore et al., 1992). PIT gives MHWs skills to empathically understand and intervene more effectively with these clients, which may increase their sense of reward and efficacy in their role and decrease burnout.

EBS education & prevention/resiliency training. One study (Duckworth McNeil, 2012) highlighted that secondary traumatic stress disorder is thought to occur when second-hand trauma is not integrated (Harris, 1995). Thus, teaching MHWs about this may enable them to better respond to their experiences. Salyers et al. (2011) combined burnout-reduction principles such as boundary setting with mindfulness techniques.For the study teaching MHWs to deliver Certified Compassion Fatigue Specialist Training (CCFST); it was hypothesised that that this may reduce their own CF due to exposure to theories of EBS they learnt in the process (Gentry et al., 2004). The study utilising resiliency training delivered a workshop- the Professional Resilience and Optimization workshop (Gentry & Monson, 2017)- which is based in the same recovery program as the CCFST- the Accelerated Recovery Program (Gentry & Baranowsky, 1998). The Accelerated Recovery Program is a manualised treatment for people experiencing compassion fatigue. The proposed effectiveness of the Professional Resilience and Optimization workshop was linked to various separate mechanisms that did not constitute a unified theory and are summarised in Table 7. However the study utilising this program made reference to emotional contagion theory (Figley, 1995; Rothschild & Rand, 2006) and proposed that the resiliency skills being taught would act as ‘antibodies’ of CF that would allow participants to resist its effects (Ballew, 2020).

Mindfulness/compassion. Mindfulness and self-compassion encourage non-judgmental attitudes towards experiences and may therefore decrease unhelpful coping strategies and increase willingness to accept and experience negative emotions that might arise (Raab, 2014).

This is a provisional file, not the final typeset article

ACT. Studies referenced how key components of ACT e.g. psychological flexibility, cognitive de-fusion and acceptance may reduce impact and believability of negative thoughts and feelings that arise from difficulties/stress in working with clients (Hayes, 2004; Hayes et al., 2006).

Supervision. Studies theorised that supervision may decrease burnout as support systems positively influence people's ability to cope with work stress (Pines & Aronsson, 1988; Wessells et al., 1989). It has also been found to help counsellors manage stress and prevent burnout (e.g. Moyer, 2011; Thompson et al., 2011). The study specifically using affective check-in supervision drew on literature suggesting that secondary trauma responses in trauma counsellors could be mitigated by supervision practices that validate their experiences of being personally impacted both cognitively and emotionally by their work as well as giving them skills to manage this (e.g. Canfield, 2005; Knight, 2013; Whitfield & Kanter, 2014).

Arts therapy. Expressive arts therapies can alleviate compassion fatigue (Pearlman & Saakvitne, 1995) and their inclusion in MHWs' supervision can increase self-awareness and reduce stress (e.g. Deaver & Shiflett, 2011; Fish, 2017). The study using drumming highlighted that this has been used in cultural healing practices since the beginning of ancient history (Bittman et al., 2001) and found to have positive physiological, psychological, and social effects and has been trialled on patients with mental health difficulties (e.g. Bensimon et al., 2008). The study using poetry referenced how writing may help in the integration of traumatic memories (Metcalf & Jacobs, 1996).

Wellness/stress reduction. This group of studies provided a diverse range of rationales for their interventions which largely were not linked to explicit theories and are captured in Table 7. However, one stress management study highlighted work by Holroyd (1979) suggesting that people can be trained to observe how environmental stressors impact their behaviour and cognition and thus choose more adaptive responses to stress.

Table 7

Evidence base provided by studies without an explicit theoretical basis for intervention

**Intervention Type**	**Evidence Base**
CBT (plus supervision)	The two studies training MHWs in CBT skills emphasised the format of training delivery and supervision above the content, i.e. highly structured, inflexible methods vs flexible and responsive training and supervision. They drew on research findings that indicate that supportive organizations that promote flexibility and autonomy may reduce job burnout (Garner et al., 2007; Knudsen et al., 2003); whereas highly centralised management practices are associated with higher levels of emotional exhaustion among counselling staff (Knudsen et al., 2006).
Primary nursing	No research explicitly investigated the impact of primary nursing (PN) on burnout. However, it was hypothesised that it might alleviate it given that low autonomy is associated with higher burnout in nurses (Reed, 1988), and PN increases autonomy (MacGuire & Botting, 1990).
Peer/relationships	Studies noted that social support is negatively correlated with burnout (Pines & Kafry, 1978) and that sharing one's emotions with others who are also experiencing difficulties can be therapeutic (Festinger, 1954; Schachter, 1968; Shubin, 1987). In addition, lack of reciprocity amongst colleagues and unhelpful comparisons with peers seem to have a part in the development of burnout (Schaufeli, 2003; Buunk et al., 2001; Bakker et al., 2000). The study utilising a social connectedness intervention based this on the Interpersonal Process Model (IPM; Reis & Shaver,1988), wherein a turn-by-turn relational process is thought to establish psychological intimacy. They highlight evidence suggesting that when members of a dyad engage in reciprocal self-disclosure and respond to one another with care and validation, this creates feelings of closeness and intimacy between them (e.g., Laurenceau et al., 1998; Laurenceau et al., 2005).
DBT	Within the DBT model, the behaviours of clients with BPD that increase risk of burnout in MHWs (e.g. excessive contact with MHWs, demands for more regular therapy or threats towards MHWs) are considered therapy-interfering behaviours (Linehan, 1993). The model therefore incorporates a consultation group which aims to support and encourage MHWs, whilst maintaining their adherence to the model and identifying when clients are pushing boundaries so that this can be swiftly addressed. This is thought to reduce risk of burnout. In addition, emphasis on DBT clinicians practicing the core DBT skills of distress tolerance, mindfulness, emotion regulation, and interpersonal effectiveness is thought to be protective against burnout (Dimeff & Linehan, 2001; Linehan, 1993).

Intervention Type	Evidence Base	
Balint-like groups	The study utilising Balint-like groups cited research demonstrating that these groups target various outcomes including burnout and wellbeing (Van Roy et al. 2015). In addition it was noted that physicians have reported the groups decrease feelings of isolation and support their processing of emotional interactions (Schwartz et al., 2020) as well as increasing perceptions of social support for physicians working in palliative care (Popa-Velea et al., 2019).	
Wellness/stress reduction	Stress Reduction workshop + follow up meetings	The study employing stress-reduction/positive imagery workshops highlighted that stress-reduction workshops can alter the course of occupational burnout via integrative methods including practical guidance and emotional support (Pines & Aronson, 1988). However, they point out that workshops alone are insufficient and frequent follow up is needed to maintain the changes in coping styles that can be introduced (Edelwich & Brodsky, 1980).
	Wellness programme	This study suggested that the energy resulting from compassion fatigue can positively impact compassion satisfaction if handled differently Radley and Figley (2007). They cite findings that reduction in CF and gains in compassion satisfaction have been demonstrated for: Expressive writing (Henry, 2014), guided imagery (Kiley et al., 2018), yoga and mindfulness (Gregory, 2015), and music therapy (Hilliard, 2006).
	Yoga-Based Stress Management	The study authors had devised this novel method through combining yoga with aspects of cognitive stress management. They cite research that yoga and cognitive stress management programs are associated with improved in wellbeing and stress reduction (Granath et al., 2006). The authors also suggested the intervention was similar to MBSR and created opportunities to practice mindfulness (unreferenced).
	Guided Imagery	The study using guided imagery cited findings that imagining an activity produces similar physiological reactions to actually carrying it out (Morewedge et al., 2010; Pascual-Leone et al., 1995), and therefore suggested that calming and peaceful imagery may produce similar responses to real situations of tranquility.
	Reiki	The study using Reiki described how this energy-based treatment promotes energetic balance in recipients that holistically addresses emotional, physical and spiritual aspects of their self to balance and heal their energy (Mitchell, 1994).

Note. CBT= cognitive behavioural therapy; CF= compassion fatigue BPD = borderline personality disorder; DBT = dialectical behaviour therapy; MBSR = mindfulness based stress reduction; MHWs – mental health workers; PN = primary nursing

Measures

An overview of the quantitative measures of EBS used in included studies is provided in Appendix C. Variations of the Maslach Burnout Inventory were most commonly used (n = 28) consistent with the fact that burnout was the most common outcome targeted in the included studies. Of the studies utilising qualitative/ mixed methods (n = 8), qualitative data collection was done via interviews (n = 4); free text/semi-structured questionnaires (n = 3); art created by participants (n =1); focus groups (n =1) and narrative feedback (n =1).

The majority of studies (n = 49) collected EBS measures both pre and post intervention. Most of these (n = 30) only collected post data at one time point. Of these 30 studies, 25collected post measures within two weeks of the intervention end. However, some (n = 6) collected post measures between five weeks and 12 months post intervention. Four studies comprising long-term interventions (supervision; DBT training) took multiple measures throughout intervention phases. Twelve studies collected additional follow up data after the initial post data, either once (n = 11) twice (n = 2) or three times (n = 1). Timeframes for additional post data ranged from two to 18 months. Finally, one study (Rollins et al., 2016) collected their first post-intervention data at six weeks following intervention, and then a further follow up at six months.

Discussion

This scoping review is, to our knowledge, the first to describe the available evidence on all available interventions for EBS in MHWs as a distinct professional group. In addition, it aimed to consider the theoretical underpinnings for available interventions; determine how EBS has been measured within included studies; and to make recommendations for future research. The findings relating to each of these aims will be discussed below, with reference to the wider literature. Overall, the group of studies identified in this review were heterogeneous and differed substantially with regards to: the interventions used; the length

This is a provisional file, not the final typeset article

and delivery of interventions and the rationale for applying them. We discuss how the lack of consistency across these areas, coupled with the conceptual difficulties in the field, create issues for EBS intervention literature. In response, we put forth a research agenda with recommendations for how future researchers might navigate and address these difficulties.

Available interventions for EBS

A total of 51 studies were identified, most of which (n=41) targeted burnout. Studies also targeted STS and CF, though none targeted VT. The overwhelming focus on burnout is notable;, according to the EBS model proposed by Rauvola et al (2019), STS, CF and VT are all forms of empathy-based strain that drive the process of EBS, whilst burnout is one of the resulting adverse outcomes. Through the EBS lens, the majority of interventions described in this review are therefore targeting one of the end results of EBS, rather than addressing the three active constructs. Similarly, all studies aimed to address EBS already present in MHWs (rather than solely aiming to prevent it). This suggests a recognition of the scale of the problem in the mental health work force (NHS England, 2018; NHS, 2023), however is further suggestive of an emphasis on cure over prevention

Participants

There was considerable variation in the specific roles/professions of MHWs included studies, with nurses working in a mental health setting/capacity being the profession most represented. Other professions included psychiatrists, psychologists, social workers, expressive arts therapists, occupational therapists and health care assistants; thus the collective sample of the review differed considerably with regards to the nature and amount of training they would have had to complete their role.

Of further importance was participant ethnicity, which was reported by only 20 studies. Of these, eight did not provide any information on participants who were not White, or referred

to them as ‘other’/ ‘non-white’. The omission and/or inadequate description of participant ethnicity is significant in light of research demonstrating that NHS staff belonging to ethnic minorities (in the context of the population of the UK) may face additional stress in the workplace. The NHS staff survey (NHS, 2023a) showed that one in five staff from minority ethnic groups (other than white minority groups) experienced discrimination in the 12 months preceding the survey. The latest version of the Workforce Race Equality Standard (WRES) report showed that only 44.4% of Black and minority ethnic (BME[2]) NHS staff felt that their organisation offered equal opportunities for career progression; compared with 58.7% of White staff (NHS England, 2023b). Ethnic minority NHS staff have reported in interview data the racism and racial microaggressions they face at work and the lack of equal opportunities in their professional roles (Ross et al., 2020). Thus, there is substantial evidence that MHWs, along with other NHS staff, may face additional workplace stress and potentially even racial trauma as a result of belonging to an ethnic minority. If organisations and researchers are not monitoring ethnic diversity when designing and implementing EBS interventions, then these are unlikely to be sufficiently inclusive or sensitive to the additional stressors faced by MHWs in minority ethnic groups.

Theoretical underpinnings and assumptions

There was significant variation in methodology, suggesting little consensus about how research exploring EBS should be approached. To better summarise the available elements, studies were grouped into three categories based on the level at which they sought to intervene, namely individual (including personal resilience; awareness-raising and role training); team and organisational approaches to addressing EBS. To contextualise these

[2] It is acknowledged that there is a helpful ongoing debate regarding the use of the term BME (see Aspinall, 2020 for a critical review), which is not widely accepted by the groups it represents yet continues to be used. This review uses the above term only in order to refer to data from existing reports which have used it.

This is a provisional file, not the final typeset article

findings, these levels were considered alongside the three types of organisational interventions identified by Tetrick and Quick (2011).

This comparison demonstrates that all but one of the interventions were of the secondary type (Tetrick & Quick, 2011) although they were operating at various levels of individual and team intervention. The only intervention possibly acting at the organisational level and of primary type (Tetrick & Quick, 2011) was the primary nursing study by Melchior et al. (1996). Primary nursing is a model of nursing care delivery where each patient's care is the responsibility of one nurse, with care focusing on the needs of the patient rather than the ward (Gardner, 1991). Therefore, whilst this may be applicable to inpatient psychiatric settings, it is not widely applicable to MHWs more generally.

In terms of the rationale for specific interventions, several studies provide an evidence base rather than an explicit theoretical rationale. These studies generally noted EBS is negatively correlated with: flexibility and autonomy (flexible CBT format; primary nursing); social support and emotional reciprocity with colleagues (peer support/relationships) and therapeutic boundaries (DBT training). Aside from Van Kirk (2020), studies utilising wellness/stress reduction interventions did not cite research specifically on EBS; however, they highlighted evidence of the positive effects of these interventions on stress and wellbeing in general.

Where theoretical rationales were provided, a common thread appeared to be teaching MHWs to respond differently to the EBS inducing aspects of their jobs. For ACT, mindfulness and compassion-based interventions, this took the form of taking accepting and non-judgemental attitudes towards negative thoughts and feelings that might arise from work. For EBS prevention and education, the emphasis was on teaching MHWs the causes and signs of EBS so they could better respond to negative experiences. For art therapy, the emphasis was on resolving mental health issues and integrating traumatic experiences

through various therapeutic art forms. PIT, supervision and some aspects of EBS-awareness (e.g. boundary setting) were the only interventions that suggest a preventative element. However, the onus was still on the MHW to change an aspect of their practice or complete an additional task. This seems consistent with the perspective of Montgomery (2014), who suggested healthcare systems which are still dominated by the medical model take a pathogenic perspective on burnout as a problem to be 'treated' at the individual level, rather than questioning why the organisations consistently produce this issue.

Such a focus on the individual echoes findings from Cocker and Joss' (2016) review of interventions to reduce CF in healthcare, emergency and community services workers which found all 13 included studies had an individual focus, and the majority employed stress reduction and/or holistic interventions (e.g. yoga, meditation). Several reviews of EBS interventions for healthcare staff have focused only on mindfulness-based interventions (e.g. Conversano et al., 2020; Gilmartin et al., 2017; Klein et al., 2020; Mensah & Anderson, 2015). Thus, the wider literature seems to share the emphasis on individual interventions to EBS that were found in this review.

However, studies of factors influencing EBS suggest organisational elements are key. For example, Singh et al. (2020) demonstrated job-related factors including work environment, workload and workplace trauma were associated with CF in MHWs. One of the most consistently highlighted predictors of EBS across healthcare providers is high caseload/client contact (Lasalvia et al., 2009; Maslach et al., 2001; Turgoose and Maddox, 2017; Singh et al., 2020). In a systematic review of burnout determinants, O'Connor et al. (2018) concluded that reasonable caseloads, clinician autonomy, good team functioning, and proper supervision should be the focus of organisational attempts to prevent and reduce burnout in MHWs. Sutton et al (2022) meanwhile, found in their narrative synthesis of organisational factors that ameliorate EBS in MHWs that regular supervision, balanced and diverse caseloads, strong

peer support networks and an organisational culture that acknowledges secondary trauma were key.

Furthermore, authors in this field have emphasised that organisations employing MHWs have a responsibility to prevent and reduce EBS (Abendroth & Figley, 2013) and warn that failure to address the systemic nature of this problem is resulting in significant clinician distress (Dean et al., 2019). As Killian (2008) noted upon finding no significant relationship between individual self-care strategies and reported levels of EBS, perhaps we should "stop expecting helping professionals to "pull themselves up by their bootstraps" by reducing their stress with standard individual coping strategies of leisure and continuing education" (pp. 42). They instead called on organisations to protect the wellbeing of MHWs by altering their workloads and giving them greater autonomy.

Also of note is the theory-practice gap between the theorised causes of EBS and the majority of interventions used to address them. STS and CF are thought to arise through traumatic exposure to the distress of others (Figley, 1995) and burnout is said to develop due to repeated interpersonal stress (Maslach, 1993). However, very few of the studies reviewed here addressed these issues directly. Of the seven studies intending to target STS, only three (Boone, 2006; Landis, 2010; Wymer, 2009) mentioned working with trauma responses. Despite the interpersonal mechanisms indicated in all descriptions of EBS; most studies utilised individual interventions, and the interventions that had relational factors centred on relationships with colleagues and supervisors, not clients (the supposed source of the EBS). It is therefore unclear how these interventions are proposing to address the root of the problem.

As described above, it is challenging to cohesively summarise the varied interventions outlined in this review. One lens through which to view these is the Job Demands- Resources (JD- R; Bakker & Demerouti, 2007) model. The JD-R assumes that all work inherently consists of job demands (JDs) and job resources (JRs). JDs are effortful, psychologically or

physically costly aspects of a job that are negatively valued; whilst JRs are rewarding, positively valued aspects of a job that offer personal development or mitigate the impact of JDs (Schaufeli & Taris, 2014). High JDs and insufficient JRs predict burnout; however, JDs may not increase burnout if workers also have access to resources such as autonomy and high- quality relationships with supervisors (Bakker et al., 2005). Using this framework, most of the included studies seem to be aiming to increase JRs, either through increasing the EBS-awareness, therapeutic skill or personal resources of individual MHWs, or through team-based efforts i.e. supervision. However a recent study found that both lowering JDs and increasing JRs is necessary to enable MHWs to provide high quality, person-centred care and recommends mental health organisations address both (Fukui et al., 2021).

Measurement of EBS

Whilst repeated measures designs were the most common, there was notable inconsistency in methodologies, as well as in the duration of interventions and the timing/frequency of follow ups. The Maslach Burnout Inventory was the most common measure used which is consistent with the majority of studies targeting burnout as a construct. Qualitative/mixed methods were rare, and there appears to have been minimal attempts to discover what factors were associated with EBS for the study populations before intervening. This may be significant given the diverse roles of MHWs samples and multiple settings featured in included studies. Bakker and Demerouti (2007) describe using a two-stage process in their research into the unique burnout risks in different jobs/settings. This begins with qualitative interviews with workers within the organisation, exploring the unique demands and resources inherent in their role. The second phase involves operationalising the specific jobs demands and resources into items in a custom-made questionnaire which is then completed by all workers. This allows for organisation-specific interventions to be implemented when seeking to address burnout. Employing similar methods with MHWs may be useful , and indeed there are examples to be found in the literature e.g. Wilkie et al (2022) report on a two year process

This is a provisional file, not the final typeset article

of developing and implementing wellness initiatives in a Canadian hospital wherein survey and consultation processes with staff led to individual, team and organisational changes (this paper is not reviewed here as the initiatives have yet to be evaluated).

It is beyond the remit of a scoping review to assess and compare effectiveness of interventions. However, it is worth noting that even superficial observations about the relative outcomes of different approaches were difficult to make due to: the amount of different interventions used; the multi-component nature of many of the interventions and the tendency for studies to report changes across multiple subscales of EBS measures.

Limitations

The literature regarding how CF, burnout, VT and STS are conceptualised is inconsistent, which may hamper the usefulness of the findings to some extent. Ravoula et al.'s (2019) concept of EBS was used by this review in an attempt to overcome this conceptual inconsistency. Whilst this enabled an inclusive search strategy; it is possible that amalgamating concepts that have related however not identical mechanisms constituted an over-simplification. Nonetheless, over-specificity may also be unhelpful. In a review that solely targeted CF, Cocker and Joss (2016) found their earliest study published in 2011 and concluded that the evidence base for these interventions is relatively recent. By including all EBS concepts, the current review spans 40 years of research and arguably captures a more representative view of attempts to help MHWs manage the impact of empathically demanding work, independent of changing conventions in terminology.

A further limitation was excluding studies in languages other than English, thus this review has an inherent bias towards evidence produced in Western, English-speaking countries and cultures. However, the search strategy took a broad approach to publication status, methodology, and type of intervention whilst retaining a specific focus on MHWs.

Research Agenda

Drawing together the findings of this review with the wider literature, we have produced a research agenda to address the current issues in this field of study and drive it forward in a manner that focuses predominantly on the urgent need for effective EBS interventions in mental health services.

Current Issues

We highlight several key issues with existing research, including:

1) Inconsistent terminology/ lack of conceptual clarity, which creates unhelpful partitions between similar areas of research
2) A disconnect between the proposed causal mechanisms of EBS and the interventions being chosen to address it
3) An emphasis on individually focused interventions, which is at odds with literature showing organisational factors are the leading cause of EBS
4) An emphasis on treating the outcomes of EBS rather than addressing or preventing the causes
5) A failure to identify from MHWs themselves what the sources of EBS are in their specific organisations before implementing preventatives or treatments
6) Potentially relevant clinical audits and initiatives being carried out in services and not widely disseminated beyond the organisation, resulting in a disconnect between knowledge held in clinical and academic arenas
7) Little regard given to diversity factors which may influence or interact with EBS, and poor reporting on diversity data such as ethnicity
8) Methodological issues including inconsistency in study design/rigor, inconsistent measurement of EBS and lack of control for profession of MHW

This is a provisional file, not the final typeset article

Suggested Solutions

1) Inconsistent terminology/ lack of conceptual clarity

Adopting and Updating the EBS Model. Greater conceptual agreement and consistency in the literature regarding EBS would be helpful to avoid duplication of effort and bring together inter-related fields of knowledge and research. We suggest that adopting Rauvola et al.'s (2019) EBS model offers a practical solution to the problem of inconsistent nomenclature in the field by distilling the core elements of the various concepts available in a dynamic process model.

We note that the EBS model was born out of a conceptual review and hope that by considering it in an applied context (i.e. how to helpfully intervene in EBS) we can support its translation into the clinical research environment. Based on the current review we therefore suggest one minor amendment. The model depicted in Rauvola et al. (2019) shows that both individual and contextual factors influence the onset of EBS. Examples of what constitutes contextual factors are given as 'emotional display norms & expectations, form/frequency of trauma exposure, support' (pp. 298). Whilst the authors' intended meaning may have been to clearly separate the two, we suggest the wording is potentially ambiguous and may not be clear enough in emphasising organisational contextual factors related to the workplace, rather than personal contextual factors. For example, 'support' and 'trauma exposure' could refer to these elements both in and outside of the workplace. We therefore suggest a clearer distinction between organisational/job role context (e.g. workload, role autonomy, degree of exposure to EBS via work role) and individual factors (e.g. trauma history, coping style, personality, support network outside work).

Testing the EBS Model. As Rauvola et al. (2019) note, empirical testing of this model is warranted e.g. identifying underlying mechanisms, determining what factors may trigger EBS, mapping how it arises over time. Additionally, determining the relative contribution of

contextual and individual factors is pertinent in light of the split described in this review between organisation-related causes of EBS and individually-focused interventions. E.g. how does the use of self-rostering systems (organisational context) to address EBS interact with caring status (individual context)?

Differentiating concepts and updating terminology. Following the testing processes described above, further clarification and differentiation of terms should be considered. It may be helpful to further disentangle the three empathy-based strain constructs (STS, VT, CF) from the concept of burnout (a proposed outcome of empathy-based strain- however acknowledged by Rauvola et al to share common features). Other longstanding terminology incorporated in the EBS model would benefit from review considering more recent empirical work. For example, a neuroscientific study by Hofmeyer et al (2020) has contested the term compassion fatigue after finding that compassion does not cause fatigue- the authors suggest that 'empathic distress fatigue' should be used instead.

The Need for Practicality and Action. Whilst consistent conceptualisations and labels are undeniably important when designing research and testing hypotheses, we note that the field has been plagued by conceptual disagreements for decades and acknowledge these are unlikely to be solved to the satisfaction of all. We therefore advocate a practical approach. What seems evident from this and other reviews is that there can be negative consequences arising from repeated empathic engagement with others' distress and that this, whatever name is given to it, translates into harmful outcomes for MHWs and clients alike. We encourage applied, organisational and structured attempts to arrive at solutions to this chronic problem, which has been made ever more pressing by the COVID-19 pandemic.

2) **Disconnect between the causes of EBS and the interventions used to address it**

Clearly Linking Interventions to Causes. We call for interventions that are theoretically and conceptually linked to the theoretical understanding of EBS. If studies are using

This is a provisional file, not the final typeset article

individual concepts such as burnout, compassion fatigure etc then there should be clear links between the assumed mechanisms of these conditions (see Table 1) and the steps being taken to reduce or prevent them., and which take account of the dynamic interplay of the various components of the model.

3) **Organisational Causes vs Individual Solutions**

More Organisational Interventions. Existing literature suggests organisational factors are the key drivers of EBS, therefore there is a need for studies which employ organisational/primary interventions for EBS in MHWs e.g. reduced/altered caseloads, greater autonomy for staff. In calling for research of this nature, we are following the example of West, Bailey and Willias (2020) who gave primary interventions the main focus in their independent review of how to support nursing and midwives in delivering high quality care. They describe several examples of primary interventions that have been used in NHS mental health trusts. For example, Langley Green acute mental health hospital (Sussex, UK) implemented a distributed leadership model under which staff could raise issues and implement changes to the service. In addition, supervision commitments were reliably increased to over 90% fulfilment per week and monthly ‘transparency boards’ monitored levels of staff training and supervision. This model led to a decrease in staff sickness over the course of implementation and medication omissions fell from 36% to 0%, illustrating the dual benefits to staff and patients alike. East London NHS Foundation Trust (mental health and community services) reviewed all their clinical audit processes with the involvement of service users and staff and ultimately ceased 85% of all audit activity. This triggered two broader initiatives to: i) identify any activities that did not add value to services (e.g. duplication of clinical recording) which the trust then acted to address; ii) commence a ‘break the rules’ campaign, encouraging staff to highlight pointless procedural rules that could be eradicated. Whilst these were not research studies, they illustrate that organisational interventions do exist and would benefit from robust evaluation.

A further reason for increasing organisational interventions is suggested by the Job Demands-Resources model (JDR; Bakker & Demerouti, 2007), which tells us that job stress and burnout arise out of an imbalance between both job demands and job resources. As noted above, the majority of existing studies are addressing EBS by providing resources rather than focusing on addressing the demands faced by MHWs. Therefore, research that addresses these inherently organisational demands is warranted.

4) **An emphasis on treating the outcomes of EBS rather than addressing or preventing the causes**

Focus On Prevention. It is easier to prevent EBS than to cure it once it has been established. As noted by NHS Employers (2022), organisations employing MHWs should aim to reduce the drivers of EBS and equip themselves to recognise and intervene early when signs of EBS emerge. This focus on prevention should be reflected in the research being carried out in this field.

5) **Failure to Ask MHWs About Sources of EBS**

Ask First, Intervene Second. Existing research suggests there are key recurring organisational factors that can lead to EBS, with high caseloads being the most consistent (e.g. Singh et al., 2020). Whilst this suggests there may be reliable trends across services, it is expected that the relevant EBS-causing factors will vary between different organisations which differ in terms of e.g. structure, remit and funding. When developing interventions to address EBS in MHWs, a suggested preliminary step is for researchers to consult with MHWs to gain an understanding of e.g. the sources/causes of EBS in their particular organisation/setting (as did the East London trust leaders in the example given in West et al., 2020), and design interventions accordingly. The two-step process described by Bakker and Demerouti (2007; see discussion section) is a further example of using specific staff concerns to generate outcome measures for establishing an interventions success.

This is a provisional file, not the final typeset article

6) **Disconnect between Clinical and Academic Domains**

Practice-informed Research. Whilst the in-house clinical initiatives and audits described in West et al. (2020) may lack specific outcome measures relevant to EBS, they highlight that organisational and primary interventions are happening. However, such data is not typically widely disseminated beyond the trust or organisation in such a way that it could be identified by this and other similar reviews or stimulate further research. We therefore recommend greater connection and collaboration between clinical and academic organisations seeking to tackle the issue of EBS. It is suggested that such collaboration could provide greater benefits for the clinical staff and services participating in studies (e.g. greater chance of having context specific and relevant issues addressed); the research teams themselves (e.g. help to identify meaningful targets for outcome monitoring ; ideas for intervention) and the quality and usefulness of the resultant findings (e.g. greater validity and relevance of findings). At the least, we advocate that further research in this area be informed by e.g., outstanding case examples, to promote the development of robust real-world research.

7) **Insufficient Attention Given to Diversity Factors**

Record and Control for Diversity. Researchers and services must attend to and address the additional stressors that may be faced by MHWs who belong to ethnic minorities (e.g. NHS, 2023a) when designing interventions for EBS e.g. by using additional measures that capture experiences of discrimination. Furthermore, they must record and control for a wide range of diversity factors when implementing and evaluating EBS interventions, to ensure these are applicable across staff who represent a diverse workforce in terms of factors including ethnicity, race, gender, gender identity, sexuality, ability.

8) **Methodological issues**

Higher Quality, Consistent Methodology. Most of the studies included in this review themselves made recommendations for future research (see Appendix D for a summary). The most consistent of these was for greater methodological rigor i.e., controlled, experimental designs and larger samples. We also note that in order for future reviews to draw conclusions about the relative effectiveness of different EBS interventions, there is a need for greater methodological consistency across studies.

Improving Outcome Measurement. The second most commonly suggested research target from included papers was for studies to have a longer duration of intervention and/or follow up prior to measurement. Several studies in the review also questioned whether changes in EBS for MHWs would lead to improved outcomes for clients and recommended this be measured. We highlight the wide variation in specific outcome measures used and suggest greater consistency in measurement tools to allow direct comparison of studies.

Controlling for Specific Role. Several studies in the review acknowledged the diverse professions and settings that MHWs may represent and suggested this be controlled for in future studies- a recommendation we echo.

Summary Recommendations

For ease of reference, we have summarised the above into bullet points intended to guide researchers undertaking further studies into interventions for EBS in MHWs. Future studies should:

- Prioritise applied intervention research over conceptual debate.
- Consider adopting Rauvola et al.'s (2019) EBS model to overcome conceptual discrepancies, including our suggestion to ensure that the 'contextual factors' aspect of the model refers specifically to organisational context.

This is a provisional file, not the final typeset article

- Carry out empirical testing of the EBS framework (if using this) and clarify terminology accordingly
- Regardless of EBS construct being targeted, ensure that the chosen intervention is theoretically and conceptually linked with the proposed cause of the problem.
- Prioritise organisational or primary interventions (e.g. caseloads, service structure, worker autonomy) over those that target individual MHW qualities or skills.
- Design studies that reduce job demands rather than solely attempting to provide job resources.
- Prioritise studies that focus on preventing EBS rather than seeking to cure it once it has been established.
- Find out from MHWs what the sources of EBS are in their particular setting before intervening.
- Collaborate with services and organisations employing MHWs to share knowledge and data between the clinical and academic realms. Be informed by outstanding case examples and examples of positive deviance in clinical practice.
- Measure and control for a range of diversity factors in participants, to ensure interventions are applicable to diverse MHWs. Consider how diversity issues such as institutional racism may interact with EBS and introduce additional measures to evaluate this.
- Design larger, more methodologically robust experimental studies that control for the setting and specific role of MHWs.
- Consider longer periods of intervention and follow up prior to/over the course of outcome measurement.
- Consistency in the EBS measures used to allow better comparison between studies.
- Consider measuring the outcomes of EBS interventions for MHWs in both MHWs and their clients in parallel.

Conclusion

This scoping review outlined the current state of the literature regarding interventions for EBS in MHWs. Overall, the findings showed that there is little consistency across available interventions; aside from the general trend towards intervening at the level of the individual. Given the additional pressures placed on mental health services by the COVID-19 pandemic, we have questioned whether this individual focus is helpful or sufficient in the context of ever-increasing need for the services MHWs provide. Finally, we have presented a research agenda detailing how these and other issues can be addressed by future researchers studying interventions for EBS in MHWs. Overall, this emphasises the need for practicality and encourages applied interventions focusing on organisational causes of EBS.

References

*indicates studies included in the review

Abendroth, M., Figley, C.R. (2013). Vicarious Trauma and the Therapeutic Relationship. In Murphy, D., Joseph, S., Harris, B. (Eds.) T[illegible]ma and the Therapeutic Relationship: Approaches to Process and Practice (pp. 111-125). London: Palgrave Macmillan.

Ahmedani, B.K. (2011). Mental Health Stigma: Society, Individuals, and the Profession. Journal of Social Work and Values Ethics. 8(2), 4-1–4-16.

*Alenezi, A., McAndrew, S., Fallon, P. (2019). Burning out physical and emotional fatigue: evaluating the effects of a programme aimed at reducing burnout among mental health nurses. International Journal of Mental Health Nursing. 28(5): 1042- 1052. https://doi-org.ezproxy1.bath.ac.uk/10.1111/inm.12608

Al-Turki, H. A., Al-Turki, R. A., Al-Dardas, H. A., Al-Gazal, M. R., Al-Maghrabi, G. H., Al-Enizi, N. H., & Ghareeb, B. A. (2010). Burnout syndrome among multinational nurses

This is a provisional file, not the final typeset article

working in Saudi Arabia. Annals of African medicine, 9(4), 226–229. https://doi-org.ezproxy1.bath.ac.uk/10.4103/1596-3519.70960

*Anderson, C. M. (1982). The effects of peer support groups on levels of burnout among mental health professionals. Dissertation Abstracts International Section A: Humanities and Social Sciences, 43(6-A), 1880–1881.

Arksey, H. & O'Malley, L. (2005). Scoping studies: towards a

methodological framework, International Journal of Social Research Methodology, 8(1), 19-32. https://doi.org/10.1080/1364557032000119616

*Askey-Jones R. (2018). Mindfulness-based cognitive therapy: An efficacy study for mental health care staff. Journal of psychiatric and mental health nursing, 25(7), 380–389. https://doi-org.ezproxy1.bath.ac.uk/10.1111/jpm.12472

Aspinall, P.J. (2020) Ethnic/Racial Terminology as a Form of Representation: A Critical Review of the Lexicon of Collective and Specific Terms in Use in Britain. Genealogy, 4, 87. https://doi.org/10.3390/genealogy4030087

Atkins, P.W.B., & Parker, S.K. (2012). Understanding individual compassion in organizations: The role of appraisals and psychological flexibility. Academy of Management Review, 37(4), 524–46. https://doi.org/10.5465/amr.2010.0490

Bakker, A. B., & Demerouti, E. (2007). The Job Demands-Resources model: State of the art. Journal of Managerial Psychology, 22(3), 309–328. https://doi.org/10.1108/02683940710733115

Bakker, A. B., Demerouti, E., & Euwema, M. (2005). Job resources buffer the impact of job demands on burnout. Journal of occupational health psychology, 10(2), 170–180. https://doi-org.ezproxy1.bath.ac.uk/10.1037/1076-8998.10.2.170

* Ballew, J.K. (2020) The effects of resiliency training on self-reported compassion fatigue and compassion satisfaction in mental health professionals and counselors-in-training [Unpublished thesis] University of Montana

Baranowsky, A. (2002). The silencing response in clinical practice: on the road to dialogue. In C.R. Figley (Ed.), Treating Compassion Fatigue, Brunner/Mazel, New York.

*Bartels-Velthuis, A.A., van den Brink, E., Koster, F., Rogier Hoenders, H. J. (2020). The Interpersonal Mindfulness Program for Health Care Professionals: a Feasibility Study. Mindfulness 11, 2629–2638. https://doi.org/10.1007/s12671-020-01477-5

Beaton, R. D., & Murphy, S. A. (1995). Working with people in crisis: Research implications. In C. R. Figley (Ed.), Brunner/Mazel psychological stress series, No. 23. Compassion fatigue: Coping with secondary traumatic stress disorder in those who treat the traumatized (p. 51–81). Brunner/Mazel.

Bensimon, M., Amir, D., & Wolf, Y. (2008). Drumming through trauma: Music therapy with post-traumatic soldiers. The Arts in Psychotherapy, 35, 34–48. https://doi.org/10.1016/j.aip.2007.09.002

Bittman, B. B., Berk, L. S., Felten, D. L., Westerngard, J., Simonton, O. C., Pappas, J., & Ninehouser, M. (2001). Composite effects of group drumming music therapy on modulation of neuroendocrine-immune parameters in normal subjects. Alternative Therapies, 7, 38–47.

*Boone, B.C. (2012). The impact of poetry therapy on symptoms of secondary posttraumatic stress disorder in domestic violence counsellors [Unpublished thesis] Office of Graduate Studies, Texas A&M University.

Borritz, M., Rugulies, R., Bjorner, J. B., Villadsen, E., Mikkelsen, O. A., & Kristensen, T. S. (2006). Burnout among employees in human service work: design and baseline findings of

the PUMA study. Scandinavian journal of public health, 34(1), 49–58. https://doi.org/10.1080/14034940510032275

Bride, B., Radey, M., Figley, C. (2007). Measuring Compassion Fatigue. Clinical Social Work Journal. 35, 155–163.

*Brady, S., O'Connor, N., Burgermeister, D., & Hanson, P. (2012). The impact of mindfulness meditation in promoting a culture of safety on an acute psychiatric unit. Perspectives in psychiatric care, 48(3), 129–137. https://doi-org.ezproxy1.bath.ac.uk/10.1111/j.1744-6163.2011.00315.x

Callender, K. A., Trustey, C. E., Alton, L., & Hao, Y. (2019). Single case evaluation of a mindfulness-based mobile application with a substance abuse counselor. Counseling Outcome Research and Evaluation. Advance online publication. https://doi.org/10.1080/21501378.2019.1686353

Canfield, J. (2005). Secondary Traumatization, Burnout, and Vicarious Traumatization: A Review of the Literature as It Relates to Therapists Who Treat Trauma. Smith College Studies in Social Work, 75, 81–101. https://doi.org/10.1300/J497v75n02_06

Care Quality Commission (2022) State of Care 2021/22. Available from: https://www.cqc.org.uk/publication/state-care-202122 [Accessed: 10.04.2023]

*Carmel, A., Fruzzetti, A. E., & Rose, M. L. (2014). Dialectical behavior therapy training to reduce clinical burnout in a public behavioral health system. Community mental health journal, 50(1), 25–30. https://doi-org.ezproxy1.bath.ac.uk/10.1007/s10597-013-9679-2

Cavanagh, N., Cockett, G., Heinrich, C., Doig, L., Fiest, K., Guichon, J. R., Page, S., Mitchell, I., & Doig, C. J. (2020). Compassion fatigue in healthcare providers: A systematic review and meta-analysis. Nursing Ethics, 27(3), 639–665. https://doi.org/10.1177/0969733019889400

Cocker, F., & Joss, N. (2016). Compassion Fatigue among Healthcare, Emergency and Community Service Workers: A Systematic Review. International journal of environmental research and public health, 13(6), 618. https://doi.org/10.1300/J497v75n02_06

*Chilton, G., Lynskey, K., Ohnstad, E., Manders. E. (2020). A Case of El Duende: Art-Based Supervision in Addiction Treatment, Art Therapy. https://doi.org/10.1080/07421656.2020.1771138

*Chochol, M. D., Pease, E., Swintak, C., & Anand, U. (2021). Addressing Well-being, Burnout, and Professional Fulfillment in Child and Adolescent Psychiatry Trainees During the COVID-19 Pandemic Through Implementation of a Virtual Balint-Like Group: a Pilot Study. Academic psychiatry : the journal of the American Association of Directors of Psychiatric Residency Training and the Association for Academic Psychiatry, 45(5), 581–586. https://doi.org/10.1007/s40596-021-01488-x

Conversano C., Ciacchini R., Orrù G., Di Giuseppe M., Gemignani A., Poli A. (2020). Mindfulness, Compassion, and Self-Compassion Among Health Care Professionals: What's New? A Systematic Review. Frontiers in Psychology. 11.1683. https://doi.org/10.1080/07421656.2020.1771138

Corcoran, K. & Fischer, J. (1994). Measures for clinical practice: A Sourcebook (3rd Ed., Vol. 2) Adults. New York: The Free Press.

*Clarke, S., Taylor, G., Lancaster, J., & Remington, B. (2015). Acceptance and commitment therapy-based self-management versus psychoeducation training for staff caring for clients with a personality disorder: a randomized controlled trial. Journal of personality disorders, 29(2), 163–176. https://doi-org.ezproxy1.bath.ac.uk/10.1521/pedi_2014_28_149

Cuneo, C. L., Curtis Cooper, M. R., Drew, C. S., Naoum-Heffernan, C., Sherman, T., Walz, K., & Weinberg, J. (2011). The effect of Reiki on work-related stress of the registered nurse.

This is a provisional file, not the final typeset article

Journal of holistic nursing : official journal of the American Holistic Nurses' Association, 29(1), 33–43. https://doi-org.ezproxy1.bath.ac.uk/10.1177/0898010110377294

Dean, W., Talbot, S., & Dean, A. (2019). Reframing Clinician Distress: Moral Injury Not Burnout. Federal practitioner: for the health care professionals of the VA, DoD, and PHS, 36(9), 400–402.

Deaver, S. P., & Shiflett, C. (2011). Art-based supervision techniques. The Clinical Supervisor, 30(2), 257–276. https://doi.org/10.1080/07325223.2011.619456

Demerouti, E. & Bakker, A.B. (2008). The Oldenburg Burnout Inventory: A good alternative to measure burnout and engagement, in: J. Halbesleben (Ed.), Handbook of stress and burnout in health care, Nova Science Publishers, Hauppauge, NY, 65-78

Demerouti, E., Bakker, A. B., Vardakou, I., & Kantas, A. (2003). The convergent validity of two burnout instruments: A multitrait-multimethod analysis. European Journal of Psychological Assessment, 19(1), 12–23. https://doi.org/10.1080/07421656.2020.1771138

Demerouti, E., Bakker, A. B., Nachreiner, F., & Schaufeli, W. B. (2000). A model of burnout and life satisfaction amongst nurses. Journal of advanced nursing, 32(2), 454–464. https://doi.org/10.1046/j.1365-2648.2000.01496.x

Demerouti, E. & Nachreiner, F. (1998). Zur Spezifität von Burnout für Dienstleistungsberufe: Fakt oder Artefakt [The specificity of burnout for human services: Fact or artefact] Zeitschrift für Arbeitswissenschaft, 52, 82-89.

Department of Health & Social Care (2012). The NHS Constitution for England. Retrieved from: https://www.gov.uk/government/publications/the-nhs-constitution-for-england#history [Accessed: 14/12/20]

Dimeff, L., & Linehan, M.M. (2001). Dialectical behavior therapy in a nutshell. The California Psychologist, 34, 10-13.

*Doyle, M., Kelly, D., Clarke, S., & Braynion, P. (2007). Burnout: The impact of psychosocial interventions training. Mental Health Practice,10,16–19. http://dx.doi.org/10.7748/mhp2007.04.10.7.16.c4298

*Duckworth McNeil, A. (2012). Identification and Prevention of Secondary Traumatic Stress in Mental Health Professionals Who Work With Child Sexual Abuse Victim [Unpublished thesis] The University of Southern Mississippi

Dyrbye, L. N., Satele, D., Sloan, J., & Shanafelt, T. D. (2013). Utility of a brief screening tool to identify physicians in distress. Journal of general internal medicine, 28(3), 421–427. https://doi.org/10.1007/s11606-012-2252-9

Dyrbye, L. N., Satele, D., Sloan, J., & Shanafelt, T. D. (2014). Ability of the physician well-being index to identify residents in distress. Journal of graduate medical education, 6(1), 78–84. https://doi.org/10.4300/JGME-D-13-00117.1

Edelwich, J. & Brodsky, A. (1980). Burn-out: Stages of Disillusionment in the Helping Professions. Human Sciences Press: New York.

Elliott, R., Bohart, A. C., Watson, J. C., & Murphy, D. (2018). Therapist empathy and client outcome: An updated meta-analysis. Psychotherapy (Chicago, Ill.), 55(4), 399–410. https://doi.org/10.1037/pst0000175

*Eriksson, T., Germundsjö, L., Åström, E., & Rönnlund, M. (2018). Mindful Self-Compassion Training Reduces Stress and Burnout Symptoms Among Practicing Psychologists: A Randomized Controlled Trial of a Brief Web-Based Intervention. Frontiers in psychology, 9, 2340. https://doi-org.ezproxy1.bath.ac.uk/10.3389/fpsyg.2018.02340

*Ewers, P., Bradshaw, T., McGovern, J., & Ewers, B. (2002). Does training in psychosocial interventions reduce burnout rates in forensic nurses?. Journal of advanced nursing, 37(5), 470–476. https://doi-org.ezproxy1.bath.ac.uk/10.1046/j.1365-2648.2002.02115.x

Festinger, L. (1954). A theory of social comparison processes. Human Relations, 7, 117-140.

Figley, C. R. (1995). Compassion fatigue: Toward a new understanding of the costs of caring. In B. H. Stamm (Ed.), Secondary traumatic stress: Self-care issues for clinicians, researchers, and educators (pp. 3–28). Baltimore: The Sidran Press.

Figley, C. R. (2002). Compassion fatigue: Psychotherapists' chronic lack of self-care. Journal of Clinical Psychology, 58(11), 1433–1441. https://doi.org/10.1002/jclp.10090

Figley, C. R., & Stamm, B. H. (1996). Psychometric review of Compassion Fatigue Self-Test. In B. H. Stamm (Ed.), Measurement of stress, trauma, and adaptation (pp. 127–130). Brooklandville, MD: Sidran Press.

*Finnamore, C., Rocca, F., Parker, J., Blazdell, J., Dale, O. (2020). The impact of a co-produced personality disorder training on staff burnout, knowledge and attitudes. Mental Health Review Journal, 25(3):269-280. https://doi.org/10.1108/MHRJ-01-2020-0009

Fish, B. (2017). Arts-based Supervison. Routledge.

*Flarity, K., Nash, K., Jones, W., & Steinbruner, D. (2016). Intervening to Improve Compassion Fatigue Resiliency in Forensic Nurses. Advanced emergency nursing journal, 38(2), 147–156. https://doi-org.ezproxy1.bath.ac.uk/10.1097/TME.0000000000000101

Fukui, S., Salyers, M. P., Morse, G., & Rollins, A. L. (2021). Factors that affect quality of care among mental health providers: Focusing on job stress and resources. Psychiatric rehabilitation journal, 44(3), 204–211. https://doi.org/10.1037/prj0000469

Garner, B. R., Knight, K., & Simpson, D. D. (2007). Burnout among corrections-based drug treatment staff: Impact of individual and organizational factors. International Journal of Offender Therapy and Comparative Criminology, 51(5), 510–522. https://doi-org.ezproxy1.bath.ac.uk/10.1177/0306624X06298708

Gardner K. (1991). A summary of findings of a five-year comparison study of primary and team nursing. Nursing research, 40(2), 113–117.

Gentry, J. E. (1996). Solution-focused trauma recovery scale (TRS). Unpublished. Florida State University: Tallahassee

Gentry, J. E., Baggerly, J., & Baranowsky, A. (2004). Training-as-treatment: effectiveness of the Certified Compassion Fatigue Specialist Training. International journal of emergency mental health, 6(3), 147–155.

Gentry, J. E.; Baranowsky, A. B. (1998). Treatment manual for the accelerated recovery program: Set II. Toronto, Canada: PsychInk.

Gentry, J. E.; Monson, M. (2017). Professional resilience: Helping doesn't have to hurt. Phoenix, AZ: Compassion Unlimited.

Gilbert, P. (Ed). (2005). Compassion: Conceptualizations, research, and use in psychotherapy. New York, NY: Routledge

Gilbert P. (2014). The origins and nature of compassion focused therapy. The British journal of clinical psychology, 53(1), 6–41. https://doi.org/10.1111/bjc.12043

Gilmartin, H., Goyal, A., Hamati, M. C., Mann, J., Saint, S., & Chopra, V. (2017). Brief Mindfulness Practices for Healthcare Providers - A Systematic Literature Review. The American journal of medicine, 130(10), 1219.e1–1219.e17. https://doi.org/10.1016/j.amjmed.2017.05.041

This is a provisional file, not the final typeset article

Granath, J., Ingvarsson, S., von Thiele, U., & Lundberg, U. (2006). Stress management: a randomized study of cognitive behavioural therapy and yoga. Cognitive behaviour therapy, 35(1), 3–10. https://doi-org.ezproxy1.bath.ac.uk/10.1080/16506070500401292

Gregory, A. (2015). Yoga and mindfulness program: The effects on compassion fatigue and compassion satisfaction in social workers. Journal of Religion & Spirituality in Social Work: Social Thought, 34(4), 372–393. https://doi.org/10.1080/15426432.2015.1080604

Grossi, G., Perski, A., Evengård, B., Blomkvist, V., & Orth-Gomér, K. (2003). Physiological correlates of burnout among women. Journal of psychosomatic research, 55(4), 309–316. https://doi.org/10.1016/s0022-3999(02)00633-5

Halbesleben, J. R. B. (2010). The role of exhaustion and workarounds in predicting occupational injuries: A cross-lagged panel study of health care professionals. Journal of Occupational Health Psychology, 15(1), 1–16. https://doi.org/10.1037/a0017634

Halbesleben, J. R. B., & Demerouti, E. (2005). The construct validity of an alternative measure of burnout: Investigating the English translation of the Oldenburg Burnout Inventory. Work & Stress, 19(3), 208–220. https://doi-org.ezproxy1.bath.ac.uk/10.1080/02678370500340728

*Hallberg I. R. (1994). Systematic clinical supervision in a child psychiatric ward: satisfaction with nursing care, tedium, burnout, and the nurses' own report on the effects of it. Archives of psychiatric nursing, 8(1), 44–52. https://doi-org.ezproxy1.bath.ac.uk/10.1016/0883-9417(94)90020-5

Hamaideh S. H. (2011). Burnout, social support, and job satisfaction among Jordanian mental health nurses. Issues in mental health nursing, 32(4), 234–242. https://doi-org.ezproxy1.bath.ac.uk/10.3109/01612840.2010.546494

Harris, C.J. (1995). Sensory-based Therapy for Crisis Counselors. In: C.R. Figley (Ed.), Compassion fatigue: coping with secondary traumatic stress disorder, 110-114. New York, NY: Brunner/Mazel.

Hayes, S. C. (2004). Acceptance and Commitment Therapy, Relational Frame Theory, and the third wave of behavior therapy. Behavior Therapy, 35, 639-665. https://doi.org/10.1016/j.beth.2016.11.006

*Hayes, S. C., Bissett, R., Roget, N., Padilla, M., Kohlenberg, B. S., Fisher, G., Masuda, A., Pistorello, J., Rye, A. K., Berry, K., & Niccolls, R. (2004). The Impact of Acceptance and Commitment Training and Multicultural Training on the Stigmatizing Attitudes and Professional Burnout of Substance Abuse Counselors. Behavior Therapy, 35(4), 821–835. https://doi-org.ezproxy1.bath.ac.uk/10.1016/S0005-7894(04)80022-4

Hayes, S. C., Luoma, J. B., Bond, F. W., Ma-suda, A., & Lillis, J. (2006). Acceptance and Commitment Therapy: Model, processes and outcomes. Behaviour Research and Therapy, 44(1), 1–25. https://doi.org/10.1016/j.brat.2005.06.006

*Haynos, A. F., Fruzzetti, A. E., Anderson, C., Briggs, D., & Walenta, J. (2016). Effects of dialectical behavior therapy skills training on outcomes for mental health staff in a child and adolescent residential setting. Journal of hospital administration, 5(2), 55–61. https://doi-org.ezproxy1.bath.ac.uk/10.5430/jha.v5n2p55

Henderson, C., Noblett, J., Parke, H., Clement, S., Caffrey, A., Gale-Grant, O. et al. (2014) Mental health-related stigma in healthcare and mental health–care settings. Lancet Psychiatry. 1(6), 467–482. https://doi.org/10.1016/S2215-0366(14)00023-6

Henry, B. J. (2014). Nursing burnout interventions: what is being done?. Clinical journal of oncology nursing, 18(2), 211–214. https://doi-org.ezproxy1.bath.ac.uk/10.1188/14.CJON.211-21

This is a provisional file, not the final typeset article

Hilliard, R. (2006). The effect of music therapy sessions on compassion fatigue and team building of professional hospice caregivers. The Arts in Psychotherapy, 33, 395–401. https://doi.org/10.1016/j.aip.2006.06.002

Hofmeyer, A., Kennedy, K., & Taylor, R. (2020). Contesting the term ‘compassion fatigue’: Integrating findings from Social Neuroscience and self-care research. Collegian, 27(2), 232–237. https://doi.org/10.1016/j.colegn.2019.07.001

Holroyd, K. (1979). Stress, coping, and the treatment of stress related illness. In J. McNamara (Ed.), Behavioral approaches to medicine. New York: Plenum Press.

Horowitz, M., Wilner, N., & Alvarez, W. (1979). Impact of Event Scale: a measure of subjective stress. Psychosomatic medicine, 41(3), 209–218. https://doi-org.ezproxy1.bath.ac.uk/10.1097/00006842-197905000-00004

*Hunnicutt, A.W., MacMillan, T.F. (1983). Beating Burnout: Findings from a three-year study. Journal of Mental Health Administration 10, 7–9. https://doi.org/10.1007/BF02833043

*Ifrach, E.R., Miller, A. (2016). Social action art therapy as an intervention for compassion fatigue, The Arts in Psychotherapy, 50, 34-39 https://doi.org/10.1016/j.aip.2016.05.009

Jachens, L., Houdmont, J., Thomas, R. (2018). Work-related stress in a humanitarian context: A qualitative investigation. Disasters, 42, 619–634. https://doi.org/10.1111/disa.12278

*Jones M. (2009). The side effects of evidence-based training. Journal of psychiatric and mental health nursing, 16(7), 593–598. https://doi-org.ezproxy1.bath.ac.uk/10.1111/j.1365-2850.2009.01401.x

Jones, J., & Lowe, T. (2001). The post-registration education and training of mental health nurses working in acute inpatient psychiatric settings. Mental Health Practice. 5 4), 8-11. https://doi.org/10.1016/S0260-6917(03)00101-1

*Kiley, K.A., Sehgal, A.R., Neth, S., Dolata, J., Pike, E., Spilsbury, J.C., Albert, J.M. (2018).The Effectiveness of Guided Imagery in Treating Compassion Fatigue and Anxiety of Mental Health Workers, Social Work Research, 42(1) 33–43, https://doi.org/10.1093/swr/svx026

Killian, K. D. (2008). Helping till it hurts? A multimethod study of compassion fatigue, burnout, and self-care in clinicians working with trauma survivors. Traumatology, 14(2), 32–44. https://doi.org/10.1177/1534765608319083

Kirby, J.N., Tellegen, C.L. and Steindl, S.R. (2017). A meta-analysis of compassion-based interventions: Current state of knowledge and future directions. Behavior Therapy, 48(6), 778-792. https://doi.org/10.1016/j.beth.2017.06.003

Klein, A., Taieb, O., Xavier, S., Baubet, T., Reyre, A. (2020). The benefits of mindfulness-based interventions on burnout among health professionals: A systematic review, EXPLORE, 16(1), 35-43. https://doi.org/10.1016/j.explore.2019.09.002.

Klimecki, O.M., Leiberg, S., Lamm, C. and Singer, T. (2013). Functional neural plasticity and associated changes in positive affect after compassion training. Cerebral cortex, 23(7), 1552-1561. https://doi.org/10.1093/cercor/bhs142

Klimecki, O.M., Leiberg, S., Ricard, M. and Singer, T. (2014). Differential pattern of functional brain plasticity after compassion and empathy training. Social cognitive and affective neuroscience, 9(6), 873-879. https://doi.org/10.1093/scan/nst060

Knight, C. (2013). Indirect trauma: Implications for self-care, supervision, the organization, and the academic institution. The Clinical Supervisor, 32(2), 224-243. https://doi.org/10.1080/07325223.2013.850139

This is a provisional file, not the final typeset article

Knudsen, H. K., Ducharme, L. J., & Roman, P. M. (2006). Counselor emotional exhaustion and turnover intention in therapeutic communities. Journal of substance abuse treatment, 31(2), 173–180. https://doi.org/10.1016/j.jsat.2006.04.003

Knudsen, H. K., Johnson, J. A., & Roman, P. M. (2003). Retaining counseling staff at substance abuse treatment centers: Effects of management practices. Journal of Substance Abuse Treatment, 24(2), 129–135. https://doi.org/10.1016/S0740-5472(02)00357-4

*Kovač, J., Krečič, M.J., Čagran, B., Mulej, M (2017). Effect of Supervision on Stress and Burnout in School Counsellors: A Case of Action Research. Systemic Practice and Action Research 30, 395–406. https://doi.org/10.1007/s11213-016-9400-9

Kristensen, T. S., Borritz, M., Villadsen, E., & Christensen, K. B. (2005). The Copenhagen Burnout Inventory: A new tool for the assessment of burnout. Work & Stress, 19(3), 192–207. https://doi.org/10.1080/02678370500297720

Lamothe, M., Rondeau, É., Malboeuf-Hurtubise, C., Duval, M., Sultan, S. (2016). Outcomes of MBSR or MBSR-based interventions in health care providers: A systematic review with a focus on empathy and emotional competencies, Complementary Therapies in Medicine, 24, 19-28. https://doi.org/10.1016/j.ctim.2015.11.001.

*Landis. E.M. (2010). Sharevision: A Collarative-Reflective, Expressive Arts Intervention To Address Trauma [Unpublished thesis] Graduate School of Arts and Social Sciences, Lesley University

Lasalvia, A., Bonetto, C., Bertani, M., Bissoli, S., Cristofalo, D., Marrella, G., . . . Tansella, M. (2009). Influence of perceived organisational factors on job burnout: Survey of community mental health staff. British Journal of Psychiatry, 195(6), 537-544. https://doi.org/10.1192/bjp.bp.108.060871

Laurenceau, J., Barrett, L. F., & Pietromonaco, P. R. (1998). Intimacy as an interpersonal process: The importance of self-disclosure, partner disclosure, and perceived partner responsiveness in interpersonal exchanges. Journal of Personality & Social Psychology, 74(5),1238–1251. https://doi.org/10.1037/0022-3514.74.5.1238.

Laurenceau, J., Barrett, L. F., & Rovine, M. J. (2005). The interpersonal process model of intimacy in marriage: A daily-diary and multi-level modelling approach. Journal of Family Psychology, 19(2), 314–323. https://doi.org/10.1037/0893-3200.19.2.314.

Leiter, M. P., & Schaufeli, W. B. (1996). Consistency of the burnout construct across occupations. Anxiety, Stress & Coping: An International Journal, 9(3), 229–243. https://doi.org/10.1080/10615809608249404

*Leykin, Y., Cucciare, M. A., & Weingardt, K. R. (2011). Differential effects of online training on job-related burnout among substance abuse counsellors. Journal of Substance Use, 16(2), 127–135. https://doi-org.ezproxy1.bath.ac.uk/10.3109/14659891.2010.526168

Linehan, M. M. (1993). Diagnosis and treatment of mental disorders. Cognitive-behavioral treatment of borderline personality disorder. Guilford Press.

*Little, L. B. (2000). Training in dialectical behavior therapy as a means of reducing therapist burnout. Dissertation Abstracts International: Section B: The Sciences and Engineering, 61(5-B), 2769.

Luken, M., Sammons, A. (2016). Systematic review of mindfulness practice for reducing job burnout. American Journal of Occupational Therapy, 70. https://doi.org/10.5014/ajot.2016.016956

Lundgren-Nilsson, Å., Jonsdottir, I.H., Pallant, J. & Ahlborg Junior, G. (2012). Internal construct validity of the Shirom-Melamed Burnout Questionnaire (SMBQ). BMC Public Health, 12(1) https://doi.org/10.1186/1471-2458-12-1

This is a provisional file, not the final typeset article

*Luoma, J. B., & Vilardaga, J. P. (2013). Improving therapist psychological flexibility while training acceptance and commitment therapy: a pilot study. Cognitive behaviour therapy, 42(1), 1–8. https://doi-org.ezproxy1.bath.ac.uk/10.1080/16506073.2012.701662

MacGuire, J. M., & Botting, D. A. (1990). The use of the Ethnograph program to identify the perceptions of nursing staff following the introduction of primary nursing in an acute medical ward for elderly people. Journal of advanced nursing, 15(10), 1120–1127. https://doi-org.ezproxy1.bath.ac.uk/10.1111/j.1365-2648.1990.tb01702.x

Maslach, C. (1982). Burnout: The cost of caring. Englewood Cliffs, NJ: Prentice Hall

Maslach, C. (1993). Burnout: A multidimensional perspective. In W. B. Schaufeli, C. Maslach, & T. Marek (Eds.). Series in applied psychology: Social issues and questions. Professional burnout: Recent developments in theory and research (p. 19–32). Taylor & Francis.

Maslach, C. (2006). Understanding Job Burnout, In: Rossi, A.M., Pamela L. Perrewé, P. L., & Sauter, S. L. Stress and Quality of Working Life: Current Perspectives in Occupational Health. Information Age Publishing

Maslach, C. & Jackson, S.E. (1981). Maslach Burnout Inventory. 2. Palo Alto: Consulting Psychologists Press

Maslach, C., Schaufeli, W.B. & Leiter, M.P. (2001). Job Burnout. Annual Review of Psychology. 52, 397-422. https://doi.org/10.1146/annurev.psych.52.1.397

Maslow, A. (1968). Toward a psychology of being. New York: Van Nostrand,

Mathieu, F. (2007). Running on empty: Compassion fatigue in health professionals. Rehab Community Care Medicine. 4, 1–7.

Mauritz, M., Goossens, P., Draijer, N., & van Achterberg, T. (2013). Prevalence of interpersonal trauma exposure and trauma-related disorders in severe mental illness. European Journal Of Psychotraumatology, 4(1), 19985. https://doi.org/10.3402/ejpt.v4i0.19985

*Mehr, L. M., Senteney, A., & Creadie, T. M. (1995). Daydreams, stress and burn-out in women mental health workers: A preliminary clinical report. Imagination, Cognition and Personality, 14(2), 105–115. https://doi-org.ezproxy1.bath.ac.uk/10.2190/103X-40HD-A1QN-7KC5

Melamed, S., Ugarten, U., Shirom, A., Kahana, L., Lerman, Y., & Froom, P. (1999). Chronic burnout, somatic arousal and elevated salivary cortisol levels. Journal of psychosomatic research, 46(6), 591–598. https://doi-org.ezproxy1.bath.ac.uk/10.1016/s0022-3999(99)00007-0

*Melchior, M. E., Philipsen, H., Abu-Saad, H. H., Halfens, R. J., van de Berg, A. A., & Gassman, P. (1996). The effectiveness of primary nursing on burnout among psychiatric nurses in long-stay settings. Journal of advanced nursing, 24(4), 694–702. https://doi.org/10.1046/j.1365-2648.1996.02457.x

Mensah, S. B., & Anderson, J. G. (2015). Barriers and facilitators of the use of mind-body therapies by healthcare providers and clinicians to care for themselves. Complementary therapies in clinical practice, 21(2), 124–130. https://doi-org.ezproxy1.bath.ac.uk/10.1016/j.ctcp.2015.01.004

Metcalf, J. & Jacobs, W. J. (1996). A “hot-system/cool-system” view of memory under stress. The National Center for Post-Traumatic Stress Disorder Research Quarterly, 7(2), 1-3. https://doi.org/10.1016/B978-0-12-800951-2.00020-0

Mitchell K. (1994). Reiki: A Torch in Daylight. St. Charles, IL: Mind River Publications.

This is a provisional file, not the final typeset article

Montgomery, A. (2014). The inevitability of physician burnout: Implications for interventions. Burnout Research. 1, 50–6. https://doi.org/10.1016/j.burn.2014.04.002

Moore E., Ball R.A. & Kuipers L. (1992). Expressed emotion in staff working with the long term adult mentally ill. British Journal of Psychiatry, 161, 802-808. https://doi.org/10.1192/bjp.161.6.802

Morewedge, C. K., Huh, Y. E., & Vosgerau, J. (2010). Thought for food: imagined consumption reduces actual consumption. Science (New York, N.Y.), 330(6010), 1530–1533. https://doi-org.ezproxy1.bath.ac.uk/10.1126/science.1195701

Moyer, M. (2011). Effects of non-guidance activities, supervision, and student-to counsellor ratios on school counsellor burnout. Journal of School Counselling, 9(5):1–31

Munn, Z., Peters, M. D. J., Stern, C., Tufanaru, C., McArthur, A., & Aromataris, E. (2018). Systematic review or scoping review? Guidance for authors when choosing between a systematic or scoping review approach. BMC medical research methodology, 18(1). https://doi.org/10.1186/s12874-018-0611-x

Newell, J.M. & MacNeil, Gordon. (2010). Professional burnout, vicarious trauma, secondary traumatic stress, and compassion fatigue: A review of theoretical terms, risk factors, and preventive methods for clinicians and researchers. Best Practice in Mental Health. 6. 57-68.

*Newman, G.F., Maggott, C., Alexander, D.G. (2015). Group drumming as a burnout prevention initiative among staff members at a child and adolescent mental health care facility. South African Journal of Psychology, 45(4), 439-451. https://doi.org/10.1177/0081246315581346

NHS (2020). NHS Staff Survey 2019: National results briefing. Available from: https://www.nhsstaffsurveys.com/Caches/Files/ST19_National%20briefing_FINAL%20V2.pdf [Accessed: 10/01/21]

NHS (2023a) NHS Staff Survey 2022: National results briefing. Available from: https://www.nhsstaffsurveys.com/static/8c6442c8d92624a830e6656baf633c3f/NHS-Staff-Survey-2022-National-briefing.pdf [Accessed 10.04.23]

NHS Employers (2022, April 9) Beating burnout in the NHS. https://www.nhsemployers.org/articles/beating-burnout-nhs

NHS England (2018). Towards commissioning for workplace compassion: a support guide. Retrieved from: https://www.england.nhs.uk/wp-content/uploads/2018/10/towards-commissioning-for-workplace-compassion-a-support-guide-v2.pdf [Accessed: 10/01/21]

NHS England (2023b) Workforce Race Equality Standard Report. Retrieved from: https://www.england.nhs.uk/publication/nhs-workforce-race-equality-standard-2022/[Accessed: 10/04/2023]

O’Connor, K., Muller Neff, D., & Pitman, S. (2018). Burnout in mental health professionals: A systematic review and meta-analysis of prevalence and determinants. European Psychiatry, 53, 74-99. https://doi.org/10.1016/j.eurpsy.2018.06.003

Ortlepp, K., & Friedman, M. (2001). The relationship between sense of coherence and indicators of secondary traumatic stress in non-professional trauma counsellors. South African Journal of Psychology, 31, 38-45. https://doi.org/10.1177/008124630103100205

Pascual-Leone, A., Nguyet, D., Cohen, L. G., Brasil-Neto, J. P., Cammarota, A., & Hallett, M. (1995). Modulation of muscle responses evoked by transcranial magnetic stimulation during the acquisition of new fine motor skills. Journal of neurophysiology, 74(3), 1037–1045. https://doi-org.ezproxy1.bath.ac.uk/10.1152/jn.1995.74.3.1037

*Paulson, L. R., Casile, W. J., & Jones, D. (2015). Tech it out: Implementing an online peer consultation network for rural mental health professionals. Journal of Rural Mental Health, 39(3-4), 125–136. https://doi-org.ezproxy1.bath.ac.uk/10.1037/rmh0000034

Pearlman. L. A., & Saakvitne. K. (1995). Trauma and the therapist: Countertransference and vicarious traumatization in psychotherapy with incest survivors. New York: W. W. Norton.

Penko, T. (1994). Izgorelost pri delu. In: Lamovec T (ed) Psihodiagnostika I. Znanstveni insˇtitut Filozofskefakultete, Ljubljana, 324–343

*Perseius, K. I., Kåver, A., Ekdahl, S., Asberg, M., & Samuelsson, M. (2007). Stress and burnout in psychiatric professionals when starting to use dialectical behavioural therapy in the work with young self-harming women showing borderline personality symptoms. Journal of psychiatric and mental health nursing, 14(7), 635–643. https://doi-org.ezproxy1.bath.ac.uk/10.1111/j.1365-2850.2007.01146.x

Peters, M., Godfrey, C., Khalil, H., McInerney, P., Soares, C. & Parker, D. (2017). 2017 Guidance for the Conduct of JBI Scoping Reviews. In: Aromataris, E. & Munn, J. (eds.) (2017) Joanna Briggs Institute Reviewer's Manual. The Joanna Briggs Institute.

Pines, A., & Aronson, E. (1988). Career Burnout. Causes & Cures. New York, NY: The Free Press.

Pines, A. and Kafry, D. (1978). Occupational tedium in the social services. Social work, 23(6), 499-507.

Poghosyan, L., Aiken, L. H., & Sloane, D. M. (2009). Factor structure of the Maslach burnout inventory: an analysis of data from large scale cross-sectional surveys of nurses from eight countries. International journal of nursing studies, 46(7), 894–902. https://doi-org.ezproxy1.bath.ac.uk/10.1016/j.ijnurstu.2009.03.004

Popa-Velea, O., Trutescu, C. I., & Diaconescu, L. V. (2019). The impact of Balint work on alexithymia, perceived stress, perceived social support and burnout among physicians working in palliative care: a longitudinal study. International journal of occupational medicine and environmental health, 32(1), 53–63. https://doi.org/10.13075/ijomeh.1896.01302

Raab, K. (2014). Mindfulness, self-compassion, and empathy among health care professionals: A review of the literature. Journal of Health Care Chaplaincy,20,95–108. https://doi.org/10.1080/08854726.2014.913876

*Raab, K., Sogge, K., Parker, N., & Flament, M.F. (2015). Mindfulness-based stress reduction and self-compassion among mental healthcare professionals: a pilot study. Mental Health, Religion & Culture, 18(6), 503-512. https://doi.org/10.1080/13674676.2015.1081588

Rauvola, R.S., Vega, D.M. & Lavigne, K.N. (2019). Compassion Fatigue, Secondary Traumatic Stress, and Vicarious Traumatization: a Qualitative Review and Research Agenda. Occupational Health Science, 3, 297–336 https://doi.org/10.1007/s41542-019-00045-1

*Ray, C.A. (1981). Holistic stress management training: a burnout strategy for mental health workers [Unpublished dissertation] North Texas State University

*Razzaque, R., Wood, L. (2016). Exploration of the Effectiveness and Acceptability of a Professional Mindfulness Retreat for Psychiatrists. Mindfulness 7, 340–348. https://doi.org/10.1007/s12671-015-0443-2

Read, J., Hammersley, P., & Rudegeair, T. (2007). Why, when and how to ask about childhood abuse. Advances in Psychiatric Treatment, 13(2), 101-110. https://doi.org/10.1192/apt.bp.106.002840

*Redhead, K., Bradshaw, T., Braynion, P., & Doyle, M. (2011). An evaluation of the outcomes of psychosocial intervention training for qualified and unqualified nursing staff

This is a provisional file, not the final typeset article

working in a low-secure mental health unit. Journal of psychiatric and mental health nursing, 18(1), 59–66. https://doi-org.ezproxy1.bath.ac.uk/10.1111/j.1365-2850.2010.01629.x

Reed S. E. (1988). A comparison of nurse-related behaviour, philosophy of care and job satisfaction in team and primary nursing. Journal of advanced nursing, 13(3), 383–395. https://doi-org.ezproxy1.bath.ac.uk/10.1111/j.1365-2648.1988.tb01433.x

Reis, H. T., & Shaver, P. (1988). Intimacy as an interpersonal process. In Duck, S. (Ed.), Handbook of personal relationships: Theory, research & interventions (pp. 367–389). Chichester: Wiley.

*Reyes Ortega, M.A., Kuczynski, A.M., Kanter, J.W., de Montis, I. A., Santos, M.M. (2019). A Preliminary Test of a Social Connectedness Burnout Intervention for Mexican Mental Health Professionals. The Psychological Record, 69, 267–276. https://doi.org/10.1007/s40732-019-00338-5

*Riley, K. E., Park, C. L., Wilson, A., Sabo, A., Antoni, M. H., Braun, T. D., Harrington, J., Reiss, J., Pasalis, E., Harris, A., & Cope, S. (2017). Improving physical and mental health in frontline mental health care providers: Yoga-based stress management versus cognitive behavioral stress management. Journal of workplace behavioral health, 32(1), 26–48. https://doi-org.ezproxy1.bath.ac.uk/10.1080/15555240.2016.1261254

*Roberts, C. (2018). The Effectiveness of Dialectical Behaviour Therapy Skills Training on Outcomes for Mental Health & Learning Disability Staff [Unpublished doctoral dissertation] Bangor University

Rogers, C.R. (1967). On Becoming a Person: A Therapist's View of Psychotherapy; Constable: London, UK,

*Rollins, A. L., Kukla, M., Morse, G., Davis, L., Leiter, M., Monroe-DeVita, M., Flanagan, M. E., Russ, A., Wasmuth, S., Eliacin, J., Collins, L., & Salyers, M. P. (2016). Comparative

Effectiveness of a Burnout Reduction Intervention for Behavioral Health Providers. Psychiatric services (Washington, D.C.), 67(8), 920–923. https://doi-org.ezproxy1.bath.ac.uk/10.1176/appi.ps.201500220

*Rosada, R. M., Rubik, B., Mainguy, B., Plummer, J., & Mehl-Madrona, L. (2015). Reiki Reduces Burnout Among Community Mental Health Clinicians. Journal of alternative and complementary medicine (New York, N.Y.), 21(8), 489–495. https://doi-org.ezproxy1.bath.ac.uk/10.1089/acm.2014.0403

Ross, S., Randhawa, M., Jabbal, J., Dahir, S., West, R., Murphy, S., Thorley, J., Joubert, H. (2020) 'A long way to go': ethnic minority NHS staff share their stories. The King’s Fund. Retrieved from: https://features.kingsfund.org.uk/2020/07/ethnic-minority-nhs-staff-racism-discrimination/index.html

Rothschild, B., Rand, .L. (2006). Help for the helper: Self-care strategies for managing burnout and stress. New York, NY: W.W. Norton & Company. [Accessed 15/07/2021]

*Scarnera, P., Bosco, A., Soleti, E., & Lancioni, G. E. (2009). Preventing burnout in mental health workers at interpersonal level: an Italian pilot study. Community mental health journal, 45(3), 222–227. https://doi-org.ezproxy1.bath.ac.uk/10.1007/s10597-008-9178-z

Schacter, S. (1967). The interaction of cognitive and physiological determinants of emotional states. In Berkowitz (Ed), Advances in Experimental Social Psychology, Vol. 1, New York : Academic Press.

Schaufeli, W. B. (1990). Opgebrd: Over de achtergronden van werkstress bij contactuele beroepen - her burnout-syndroom (Burnout: About the job stress in the human services professions - The burnout syndrome), Ad. Donker, Rotterdam.

Schaufeli, W.B., Leiter, M.P. and Maslach, C. (2009).Burnout: 35 years of research and practice. Career Development International, 14(3), 204-220. https://doi.org/10.1108/13620430910966406

Schaufeli, W.B., Leiter, M.P., Maslach, C., & Jackson, S.E. (1996). The MBI-General Survey. In C. Maslach, S.E. Jackson, & M.P Leiter (Eds.), Maslach Burnout Inventory. Manual (3rd ed., pp. 19-26). Palo Alto, CA: Consulting Psychologists Press

Schaufeli, W. B., & Taris, T. W. (2014). A critical review of the job demands-resources model: Implications for improving work and health. In G. F. Bauer & O. Hämmig (Eds.), Bridging occupational, organizational and public health: A transdisciplinary approach (p. 43–68). Springer Science + Business Media. https://doi.org/10.1007/978-94-007-5640-3_4

Schaufeli, W. B., & Van Dierendonck, D. (1993). The construct validity of two burnout measures. Journal of Organizational Behavior, 14(7), 631–647. https://doi.org/10.1002/job.4030140703

Schwartz, R., Shanafelt, T. D., Gimmler, C., & Osterberg, L. (2020). Developing institutional infrastructure for physician wellness: qualitative Insights from VA physicians. BMC health services research, 20(1), 7. https://doi.org/10.1186/s12913-019-4783-9

Shay, J. (2014). Moral injury. Psychoanalytic Psychology, 31(2), 182 191. https://doi.org/10.1037/a0036090

Shubin, S. (1978). Burnout, the professional hazard you face in nursing. Nursing, 8(7), 22-27.

Singh, J., Karanika-Murray, M., Baguley, T., & Hudson, J. (2020). A Systematic Review of Job Demands and Resources Associated with Compassion Fatigue in Mental Health Professionals. International Journal of Environmental Research and Public Health, 17(19), 6987. https://doi.org/10.3390/ijerph17196987

Sirigatti S., Stefanile C. (1993). "Adattamento e taratura per l'Italia" in MBI Maslach burnout inventory. eds. Maslach C., Jackson S. (Firenze: Organizzazioni Speciali;), 33–42.

Spandler, H., & Stickley, T. (2011). No Hope without Compassion: The Importance of Compassion in Recovery-focused Mental Health Services. Journal of Mental Health 20(6): 555–566. https://doi.org/10.3109/09638237.2011.583949

Stamm, B.H. (2002). Measuring compassion satisfaction as well as fatigue: developmental history of the compassion fatigue and satisfaction test. In: Figley CR, editor. Treating compassion fatigue. New York: Brunner-Routledge. p. 107–19.

Stamm, B. H. (2009). Professional Quality of Life: Compassion Satisfaction and Fatigue Version 5 (ProQOL). Retrieved from: /www.isu.edu/~bhstamm [Accessed 22.04.21]

Strauss, C., Taylor, B.L., Gu, J., Kuyken, W., Baer, R., Jones, F., Cavanagh, K. (2016). What is compassion and how can we measure it? A review of definitions and measures. Clinical Psychology Review, 47, 15-27. https://doi.org/10.1016/j.cpr.2016.05.004

Sutton, L., Rowe, S., Hammerton, G., & Billings, J. (2022). The contribution of organisational factors to vicarious trauma in mental health professionals: a systematic review and narrative synthesis. European journal of psychotraumatology, 13(1), 2022278. https://doi.org/10.1080/20008198.2021.2022278

*Suyi, Y., Meredith, P., & Khan, A. (2017). Effectiveness of Mindfulness Intervention in Reducing Stress and Burnout for Mental Health Professionals in Singapore. Explore (New York, N.Y.), 13(5), 319–326. https://doi-org.ezproxy1.bath.ac.uk/10.1016/j.explore.2017.06.001

Tetrick L. E., Quick J. C. (2011). Overview of occupational health psychology: public health in occupational settings, in Handbook of Occupational Health Psychology, 2nd Edn, eds Quick J. C., Tetrick L. E. (Washington, DC: American Psychological Association;), 3–20.

This is a provisional file, not the final typeset article

Thompson, E.H., Frick, M.H., Trice-Black, S. (2011). Counselor-in-training perceptions of supervision practices related to self-care and burnout. The Professional Counsellor, 1(3), 152–162, https://doi.org/10.15241/eht.1.3.152

Thompson, J. (1980). Burnout in group home houseparents. American Journal of Psychiatry, 137(6), 710-714. https://doi.org/10.1176/ajp.137.6.710

Timms, C., Brough, P., & Graham, D. (2012). Burnt-out but engaged: the co-existence of psychological burnout and engagement. Journal of Educational Administration, 50(3), 327-345. https://doi.org/10.1108/09578231211223338

Ting, L., Jacobson, J.M., Sanders, S. Bride, B.E. & Harrington, D. (2005). The Secondary Traumatic Stress Scale (STSS), Journal of Human Behavior in the Social Environment, 11 (3-4), 177-194. https://doi.org/0.1300/J137v11n03_09

Tricco, A, C., Lillie, E., Zarin, W., O'Brien, K,K., Colquhoun, H., Levac, D., Moher, D., Peters, M,D., Horsley, T., Weeks, L., Hempel, S., et al. (2018). PRISMA extension for scoping reviews (PRISMA-ScR): checklist and explanation. Annals of Internal Medicine, 169(7), 467-473. https://doi.org/10.7326/M18-0850

Trockel, M., Bohman, B., Lesure, E., Hamidi, M. S., Welle, D., Roberts, L., & Shanafelt, T. (2018). A Brief Instrument to Assess Both Burnout and Professional Fulfillment in Physicians: Reliability and Validity, Including Correlation with Self-Reported Medical Errors, in a Sample of Resident and Practicing Physicians. Academic psychiatry : the journal of the American Association of Directors of Psychiatric Residency Training and the Association for Academic Psychiatry, 42(1), 11–24. https://doi.org/10.1007/s40596-017-0849-3

Turgoose, D., & Maddox, L. (2017). Predictors of compassion fatigue in mental health professionals: A narrative review. Traumatology, 23(2), 172–185. https://doi.org/10.1037/trm0000116

*Van Kirk M. L. (2021). Employee Wellness Pilot Program. Workplace health & safety, 69(5), 192–197. https://doi-org.ezproxy1.bath.ac.uk/10.1177/2165079920976522

Van Roy, K., Vanheule, S., & Inslegers, R. (2015). Research on Balint groups: A literature review. Patient education and counseling, 98(6), 685–694. https://doi.org/10.1016/j.pec.2015.01.014

*Walker, D. (2018). Examining the Effects of a Brief Mindfulness-Based Stress Reduction Program on Burnout in Mental Health Professionals [Unpublished thesis] Grand Canyon University

*Weingardt, K. R., Cucciare, M. A., Bellotti, C., & Lai, W. P. (2009). A randomized trial comparing two models of web-based training in cognitive-behavioral therapy for substance abuse counselors. Journal of substance abuse treatment, 37(3), 219–227. https://doi-org.ezproxy1.bath.ac.uk/10.1016/j.jsat.2009.01.002

Wessells, D.T., Jr., Kutscher, A.H., Seeland, LB., Selder, F.E., Cherico, D.J., & Clark, E.J. (1989). Professional Burnout in Medicine and the Helping Professions. New York, NY: The Haworth Press.

West, M. A. & Chowla, R. (2017). Compassionate leadership for compassionate health care. In Gilbert, P. (ed.). Compassion: Concepts, Research and Applications. (pp. 237-257). London: Routledge.

Whitfield, N., & Kanter, D. (2014). Helpers in distress: Preventing secondary trauma. Reclaiming Children and Youth, 22(4), 59-61.

This is a provisional file, not the final typeset article

Wilkie, T., Tajirian, T., & Stergiopoulos, V. (2022). Advancing physician wellness, engagement and excellence in a mental health setting: a Canadian perspective. Health promotion international, 37(1), daab061. https://doi.org/10.1093/heapro/daab061

*Wymer, B. (2019). An Investigation of the Impact of a Supervision Intervention on Secondary Traumatic Stress Responses Among Counselors Treating Child Survivors of Sexual Abuse [Unpublished thesis] University of South Carolina.

Appendices

Appendix A: Preferred Reporting Items for Systematic reviews and Meta-Analyses extension for Scoping Reviews (PRISMA-ScR) Checklist

SECTION	ITEM	PRISMA-ScR CHECKLIST ITEM	REPORTED ON PAGE #
**TITLE**			
Title	1	Identify the report as a scoping review.	1
**ABSTRACT**			
Structured summary	2	Provide a structured summary that includes (as applicable): background, objectives, eligibility criteria, sources of evidence, charting methods, results, and conclusions that relate to the review questions and objectives.	2
**INTRODUCTION**			
Rationale	3	Describe the rationale for the review in the context of what is already known. Explain why the review questions/objectives lend themselves to a scoping review approach.	2-5
Objectives	4	Provide an explicit statement of the questions and objectives being addressed with reference to their key elements (e.g., population or participants, concepts, and context) or other relevant key elements used to conceptualize the review questions and/or objectives.	5-6

SECTION	ITEM	PRISMA-ScR CHECKLIST ITEM	REPORTED ON PAGE #
**METHODS**			
Protocol and registration	5	Indicate whether a review protocol exists; state if and where it can be accessed (e.g., a Web address); and if available, provide registration information, including the registration number.	8
Eligibility criteria	6	Specify characteristics of the sources of evidence used as eligibility criteria (e.g., years considered, language, and publication status), and provide a rationale.	9-10
Information sources*	7	Describe all information sources in the search (e.g., databases with dates of coverage and contact with authors to identify additional sources), as well as the date the most recent search was executed.	8, 83-35
Search	8	Present the full electronic search strategy for at least 1 database, including any limits used, such that it could be repeated.	83-85
Selection of sources of evidence†	9	State the process for selecting sources of evidence (i.e., screening and eligibility) included in the scoping review.	9-10
Data charting process‡	10	Describe the methods of charting data from the included sources of evidence (e.g., calibrated forms or forms that have been tested by the team before their use, and whether data charting was done independently or in duplicate) and any processes for obtaining and confirming data from investigators.	11
Data items	11	List and define all variables for which data were sought and any assumptions and simplifications made.	9-10
Critical appraisal of individual sources of evidence§	12	If done, provide a rationale for conducting a critical appraisal of included sources of evidence; describe the methods used and how this information was used in any data synthesis (if appropriate).	N/A
Synthesis of results	13	Describe the methods of handling and summarizing the data that were charted.	12-13
**RESULTS**			
Selection of sources of evidence	14	Give numbers of sources of evidence screened, assessed for eligibility, and included in the review, with reasons for exclusions at each stage, ideally using a flow diagram.	11-12

This is a provisional file, not the final typeset article

SECTION	ITEM	PRISMA-ScR CHECKLIST ITEM	REPORTED ON PAGE #
Characteristics of sources of evidence	15	For each source of evidence, present characteristics for which data were charted and provide the citations.	13-25

Appendix B: Full Search Strategies

- Initial searches run on 15.01.2021, 1970-2021 date filters used
  Updated searches run on 12.09.2022, 2021-2022 date filters used

PsychINFO (all databases):

(((**Any Field**: ("compassion fatigue")) *OR* (**Any Field**: ("secondary traumatic stress")) *OR* (**Any Field**: (burnout)) *OR* (**Any Field**: (burn-out)) *OR* (**Any Field**: ("vicarious trauma")) *OR* (**Any Field**: ("occupational stress")) *OR* (**IndexTermsFilt**: ("Occupational Stress") *OR* **IndexTermsFilt**: ("Compassion Fatigue")) *OR* (**Any Field**: ("compassion stress")) *OR* **Any Field**: "secondary trauma" *OR* **Any Field**: "secondary traumatisation" *OR* **Any Field**: "secondary traumatization") *AND* ((**Year**: [1970 TO 2021]))) *AND* (((AnyField:(psychotherapist*)) OR (AnyField:("forensic nurse")) OR (AnyField:("forensic psychologist")) OR (AnyField:("forensic psychiatrist")) OR (AnyField:(therapist*)) OR (AnyField:("support time and recovery worker")) OR (AnyField:("mental health employee")) OR (AnyField:(counsellor*)) OR (AnyField:("counselling psychologist")) OR (AnyField:("family therapist")) OR (AnyField:(counselor)) OR (AnyField:("mental health support worker")) OR (AnyField:("mental health social worker")) OR (IndexTermsFilt:("Mental Health Personnel")) OR (IndexTermsFilt:("Counselors") OR OR (AnyField:("mental health care provider")) OR (AnyField:("mental health personnel")) OR (AnyField:("mental health nurse")) OR (AnyField:("mental health service")) OR (AnyField:("mental health service provider")) OR (AnyField:("mental health professional")) OR (AnyField:(psychologist*)) OR (AnyField:("mental health worker"))) AND ((PublicationYear:[1970 TO 2021] OR TestYear:[1970 TO 2021])))

<u>Pubmed</u>

<u>Mesh search:</u>

("social work, psychiatric"[MeSH Major Topic] OR "social work, psychiatric"[MeSH Major Topic] OR "emergency services, psychiatric"[MeSH Major Topic] OR "psychiatric nursing"[MeSH Major Topic] OR "health personnel/psychology"[MeSH Major Topic]) AND "burnout, professional/prevention and control"[MeSH Major Topic] OR "compassion fatigue/prevention and control"[MeSH Major Topic])

<u>Keyword search:</u>

("psychotherapist*"[Title/Abstract] OR "forensic nurs*"[Title/Abstract] OR "forensic psychologist*"[Title/Abstract] OR "forensic psychiatrist*"[Title/Abstract] OR "therapist*"[Title/Abstract] OR "mental health employee*"[Title/Abstract] OR

"counsellor*"[Title/Abstract] OR "counselling psychologist*"[Title/Abstract] OR "family therapist*"[Title/Abstract] OR "counselor*"[Title/Abstract] OR "mental health support worker*"[Title/Abstract] OR "mental health social worker*"[Title/Abstract] OR "mental health care provider*"[Title/Abstract] OR "mental health personnel"[Title/Abstract] OR "mental health nurs*"[Title/Abstract] OR "mental health service*"[Title/Abstract] OR "mental health service provider*"[Title/Abstract] OR "mental health professional*"[Title/Abstract] OR "psychologist*"[Title/Abstract] OR "mental health worker"[Title/Abstract])) AND ("compassion fatigue"[Title/Abstract] OR "vicarious trauma"[Title/Abstract] OR "secondary traumatic stress"[Title/Abstract] OR "burnout"[Title/Abstract] OR "burn out"[Title/Abstract] OR "compassion stress"[Title/Abstract] OR "secondary trauma"[Title/Abstract] OR "secondary traumatisation"[Title/Abstract] OR "secondary traumatization"[Title/Abstract])

Embase

- #1 ('compassion fatigue'/exp OR 'compassion fatigue' OR 'compassion stress' OR 'vicarious trauma'/exp OR 'vicarious trauma' OR 'burnout'/exp OR burnout OR 'burn out'/exp OR 'burn out' OR 'secondary traumatic stress'/exp OR 'secondary traumatic stress' OR 'secondary trauma' OR 'secondary traumatisation' OR 'secondary traumatization') AND ([embase]/lim OR [embase classic]/lim) AND [1970-2021]/pyEBS & MHWs 08.01.212021-01-082021-01-0814187

- #2 ('vicarious trauma'/exp OR 'burnout'/exp OR 'professional burnout'/exp OR 'compassion fatigue'/exp OR 'secondary traumatic stress'/exp) AND ([embase]/lim OR [embase classic]/lim) AND [1970-2021]/pyEBS & MHWs 08.01.212021-01-082021-01-0811764

- #3 (psychotherapist* OR 'forensic nurse' OR 'forensic psychologist' OR 'forensic psychiatrist'/exp OR 'forensic psychiatrist' OR therapist* OR 'support time and recovery worker' OR 'mental health employee' OR counsellor* OR 'counselling psychologist' OR 'family therapist' OR 'counselor'/exp OR counselor OR 'mental health support worker' OR 'mental health social worker' OR 'mental health care provider' OR 'mental health personnel'/exp OR 'mental health personnel' OR 'mental health nurse' OR 'mental health service'/exp OR 'mental health service' OR 'mental health service provider' OR 'mental health professional'/exp OR 'mental health professional' OR psychologist* OR 'mental health worker'/exp OR 'mental health worker') AND ([embase]/lim OR [embase classic]/lim) AND [1970-2021]/pyEBS & MHWs 08.01.212021-01-082021-01-08121632

- #4 ('mental health care personnel'/exp OR 'psychotherapist'/exp OR 'counselor'/exp OR 'mental health service'/exp OR 'psychologist'/exp OR 'psychiatric nurse'/exp OR 'forensic psychology'/exp) AND ([embase]/lim OR [embase classic]/lim) AND [1970-2021]/pyEBS & MHWs 08.01.212021-01-082021-01-0856216
- #5 #1 OR #2
- #6 #3 OR #4

This is a provisional file, not the final typeset article

- #7 #5 AND #6

PTSDPubs:

MAINSUBJECT.EXACT("Vicarious Traumatization") OR MAINSUBJECT.EXACT("Burnout") OR MAINSUBJECT.EXACT("Trauma Contagion") OR "compassion fatigue" OR "compassion stress" OR "secondary traumatic stress" OR "vicarious trauma" OR burnout OR burn-out
AND
MAINSUBJECT.EXACT("Mental Health Personnel") OR (psychotherapist* OR "forensic nurse" OR "forensic psychologist" OR "forensic psychiatrist" OR therapist* OR "support time and recovery worker" OR "mental health employee" OR counsellor* OR "counselling psychologist" OR "family therapist" OR counselor OR "mental health support worker" OR "mental health social worker" OR "mental health care provider" OR "mental health personnel" OR "mental health nurse" OR "mental health service" OR "mental health service provider" OR "mental health professional" OR psychologist*)

Central:

"compassion fatigue" OR "compassion stress" OR "secondary traumatic stress" OR "vicarious trauma" OR burnout OR burn-out OR "Compassion Fatigue" OR "burnout, professional" OR "burnout, psychological" OR "burnout, professional/prevention and control" OR "compassion fatigue/prevention and control" in Title Abstract Keyword AND 'social work, psychiatric' OR 'emergency services, psychiatric' OR 'psychiatric nursing' OR 'health personnel, psychology' OR "psychotherapist*" OR "forensic nurs*" OR "forensic psychologist*" OR "forensic psychiatrist*" OR "therapist*" OR "mental health employee*" OR "counsellor*" OR "counselling psychologist*" OR "family therapist*" OR "counselor*" OR "mental health support worker*" OR "mental health social worker*" OR "mental health care provider*" OR "mental health personnel" OR "mental health nurs*" OR "mental health service*" OR "mental health service provider*" OR "mental health professional*" OR "psychologist*" OR "mental health worker" in Title Abstract Keyword - with Cochrane Library publication date Between Jan 1970 and Jan 2021

Appendix C

Table showing quantitative measures of EBS used

Measure	Description	No. of Studies Used In
The Maslach Burnout Inventory (MBI) (Maslach & Jackson, 1981).	The MBI is a 22-item self-report measure of burnout in human services professionals. It produces 3 dimensions of burnout: emotional exhaustion, depersonalization, and personal accomplishment. It is the most commonly used measure of burnout with high reliability (α = .71 or higher) across 7 countries (Poghosyan et al., 2009).	24
The Dutch version of the MBI (DMBI) (Schaufeli, 1990).	The questionnaire consists of 22 items on a 7-point Likert scale. The Dutch MBI has been shown to be valid and reliable in the assessment of burnout in nurses (Schaufeli & van Dierendonck, 1993).	1
The Arabic version of the MBI (Al-Turki et al., 2010; Hamaideh, 2011).	The Arabic MBI has been used in a number of Arabic-speaking countries, in studies assessing burnout levels among nurses (Al-Turki et al., 2010; Hamaideh, 2011). It has been shown to have good internal consistency across subscales: α = .80 for emotional exhaustion; α =.73 for depersonalisation; α =.77 for personal accomplishment (Sabbah et al., 2012).	1
The Italian version of the MBI (Sirigatti & Stefanile, 1993).	This Italian version of the MBI has been shown to have good internal consistency across subscales: α = .88 for emotional exhaustion; α =.70 for depersonalisation; α =.83 for personal realisation (Maslach & Jackson, 1981; Sirigatti & Stefanile, 1993).	1
MBI-General Survey (MBI-GS) (Schaufeli et al., 1996).	The MBI-GS is a 16-item self-report measure of burnout in any occupation (not just health/care). The generic items measure burnout on 3 subscales that parallel the MBI: exhaustion, cynicism, and	1

This is a provisional file, not the final typeset article

Measure	Description	No. of Studies Used In
	professional efficacy. The MBI-GS has been shown to have satisfactory internal consistency (α = .84 to .90 for exhaustion, .74 to .84 for cynicism, and from .70 to .78 for professional efficacy) (Leiter & Schaufeli, 1996).	
The Copenhagen Burnout Inventory (CBI) (Kristensen et al., 2005).	The CBI is a 19-item self-report tool that produces 3 scales measuring personal burnout, work-related burnout, and client-related burnout. It is designed for use with employees in any sector. A prospective study of burnout in human service workers sector found that all 3 scales had high internal reliability α =.87 for both personal and work-related burnout; α = 0.85 for client-related burnout) (Borritz et al., 2006).	3
The Oldenburg Burnout Inventory (OLBI) (Demerouti & Nachreiner, 1998).	The OLBI is a self-report measure consisting of 16 positively and negatively formulated items that are used to evaluate 2 dimensions of burnout: the identification continuum (ranging from disengagement to dedication) and the energy continuum (ranging from exhaustion to vigour). Studies have demonstrated the convergent validity of the OLBI and the MBI-General Survey (Demerouti et al., 2003; Halbesleben & Demerouti, 2005). The reliability of the exhaustion subscale ranges from α = .74 to α = .85, and the reliability of the disengagement subscale from .73 to .85 (Demerouti & Bakker, 2008; Demerouti et al., 2003; Halbesleben & Demerouti, 2005, Halbesleben, 2010; Sonnentag, Binnewies & Mojza, 2010; Timms et al., 2012).	2
Silencing Response Scale (SRS) (Baranowsky, 2002).	The SRS is a 15-item scale that helps care-givers identify specific communication difficulties in their trauma work with clients i.e. selective listening and active avoidance. It has been shown to have good reliability, α = .85 (Ortlepp & Friedman, 2001).	1
The Trauma Recovery Scale (Gentry, 1996).	The TRS is a 10-item self-report scale. Parts I & II provide a traumatic experiences inventory. Part III measures relative recovery from traumatic experiences. It is found to have good internal reliability (*a* = .86) and good convergent validity with the impact of events scale (.71). (Gentry, 1996).	1
Secondary Traumatic Stress Scale (STSS) Bride et al., 2004).	The STSS is a 17-item self-report measure of secondary trauma in practitioners who have experienced traumatic stress through their work with clients. It comprises 3 subscales; Intrusion, Avoidance, and Arousal. It was found to have good internal consistency, ranging from α = .93- 94 for the total STSS	1

Measure	Description	No. of Studies Used In
	scale; α = .79-80 for the Intrusion subscale, α = .85- 87 for the Avoidance subscale, and α = .83-87 for the Arousal subscale (Bride et al., 2004; Ting et al., 2005).	
The Impact of Events Scale (IES) (Horowitz et al., 1979).	The IES is a 15-item self-report measure of current subjective distress for any specific life event. It measures distress along two subscales: Intrusive and Avoidance symptoms. The IES has shown very good internal consistency (*r* = .79 to .92, with an average of .86 for the intrusive subscale and .90 for the avoidance subscale) (Corcoran and Fischer, 1994). The split-half reliability of the IES scale was high (*r* =.86) and internal consistency of the subscales was also high: α =.78 for intrusion and α =.82 for avoidance. Test-retest reliability was high: *r*= .87 for the total stress scores; .89 for the intrusion subscale; and .79 for the avoidance subscale (Horowitz et al., 1979).	1
The questionnaire on the perception of occupational burnout (Penko, 1994).	According to the information in Kovač et al (2016), this questionnaire consists of 3 dimensions of occupational burnout: less fulfilment, emotional exhaustion and depersonalization. No further information was accessible in English regarding this scale.	
Professional Quality of Life Scale version 5 (ProQOL-5) (Stamm, 2009).	The ProQOL-5 is a 30 item self-report measure of the positive and negative effects of working with people who have experienced extremely stressful events. It comprises of 3 subscales measuring Compassion Fatigue, Burnout and Compassion Satisfaction. CS alpha reliabilities = .87 BO alpha reliabilities = .72 CF alpha reliabilities = .80. Early information on test-retest data show good reliability.	6
Swedish version of the Shirom Melamed Burnout Questionnaire (SMBQ) (Grossi et al., 2003)	The SMBQ (originally by Melamed et al., 1999) is a 22-item self-report burnout measure consisting of 4 subscales: Physical Fatigue; Cognitive weariness; Tension and Listlessness. The Swedish version was shown to have consistencies (α>0.70) across the subscales, but a Rasch-analysis suggested omission of the Tension factor (Lundgren-Nilsson et al., 2012).	1

This is a provisional file, not the final typeset article

Measure	Description	No. of Studies Used In
Compassion Satisfaction/Fatigue Self-Test (CSFST) (Figley & Stamm, 1996).	The CSFT is a 66-item self-report measure comprised of three subscales: compassion fatigue, compassion satisfaction, and burnout. According to Stamm (2002), all 3 subscales have demonstrated good alpha reliability: α =.87 on the compassion fatigue subscale; α =.90 on the burnout subscale; and α =.87 for the compassion satisfaction subscale.	3
Well-being Index (WBI) (Dyrbye et al., 2014)	The WBI is a seven item self-report index, measuring domains of burnout, depression, stress, fatigue, and mental and physical quality of life. It consists of yes/no items and respondents receive a score from 0 to 7 based on responses. At a threshold score of ≥ 4, the PWBI's sensitivity for identifying physicians with low mental quality of life is 73.3 % with a specificity of 81.0 % (Dyrbye et al. 2013).	1
Stanford Professional Fulfilment Index (PFI) (Trockel et al., 2018)	The PFI is a 16 item self-report measure assessing the degree of intrinsic positive reward the individual derives from his or her work, including happiness, meaningfulness, contribution, self-worth, satisfaction, and feeling in control when dealing with difficult problems at work. It consists of 3 scales- professional fulfillment; work exhaustion; and interpersonal disengagement and an overall burnout scale. Response options are on a five-point Likert scale ("not at all true" to "completely true" for professional fulfillment items and "not at all" to "extremely" for work exhaustion and interpersonal disengagement items). Each PFI item is scored from 0 to 4, using the associated five-point Likert scale. All scales have demonstrated good alpha reliability: α =.86 on the work exhaustion scale; α =.92 on the interpersonal disengagement scale; α =.91 on the professional fulfillment scale; α =.92 on the overall burnout scale (Trockel et al., 2018).	1
Stress Rating Form & Burnout Adjectives (described in Ray, 1981).	The stress rating form was devised for the study to collect pre- and post-ratings on participants by one close friend/relative and co-workers or supervisor. The observer was asked to rate the amount of stress that he/she believed that the subject was currently experiencing in five areas (home, job interpersonal relationships, health, and school), and to rate the amount of burnout the subject was feeling, on a 1 to 10 scale, with 1 being little stress and 10 being highly stressed. Additionally, the observer rated the subject on 16 adjectives on a 1 to 10-point scale, in order to compute an overall burnout score (the method for this is reportedly described in Thompson (1980) but this could not be accessed by reviewers).	1

Appendix D

Table showing recommendations for future research

Intervention	Recommendations/ Questions for future research
DBT skills training	• Larger and more diverse samples; control groups<br>• Direct patient-related outcomes<br>• Compare effectiveness of person-directed interventions, versus organizational interventions only, versus a combination.<br>• Would longer training and supervision groups increase effectiveness?<br>• Include clinician burnout as a variable within DBT effectiveness and efficacy studies<br>• Account/control for different settings differing initial levels of clinician burnout<br>• Account for variations in learning and utilization of training<br>• Administer questionnaires to assess subjects' knowledge of and utilization of DBT.
PSI training	• Focus on PSI competencies<br>• Longer-term evaluation of implementation in practice<br>• Outcome-based intervention trials<br>• Larger samples, different settings, more rigorous methodologies
ACT training	• Compare the impact of brief interventions based on ACT, psychoeducation, or an amalgam of both.<br>• Improved rigour
Supervision	• Larger samples, inclusion of different settings/specific roles of participants.<br>• What structure of systematic clinical supervision is most effective?<br>• Longer duration of study/supervision may be required to see changes in burnout<br>• Incorporate qualitative and quantitative data to measure STS responses.<br>• Can supervision serve as a preventative strategy for MHWs who do not yet have STS responses?

This is a provisional file, not the final typeset article

Intervention	Recommendations/ Questions for future research
EBS training/ prevention	• Larger samples, collect demographic data<br>• Separate the various components of the intervention for comparison<br>• Longer and more intensive STS education program<br>• Follow up 2-3 months later, giving MHWs time to implement/ incorporate STS prevention plans<br>• Exploring the effects of intermittent booster sessions of the intervention<br>• More rigorous designs e.g. multiple baseline/ wait-list control groups, randomization<br>• Measure primary traumatic stress and burnout<br>• Participant interviews to distill the active elements of interventions
Resiliency training	• More CF exploratory longitudinal studies with treatment and control groups<br>• Additional CF research exploring CF over the course of clinicians' careers<br>• Comparing CF symptoms between CITs and practicing mental health professionals<br>• Qualitative approaches exploring lived experiences with CF symptoms as well as during and after CF treatments to determine active ingredients for meaningful CF symptom reduction.
Mindfulness/ compassion	• Are MBIs alone sufficient to generate self-compassion?<br>• Increased rigour: large, controlled studies; random sampling across settings; long term follow up<br>• Explore possible applications to other disciplines/ health workers<br>• Explore mechanisms through which the Interpersonal Mindfulness Program works<br>• Explore the adverse reactions reported in one study (Bartels-Velthuis et al., 2020).
'BREATHE' burnout reduction workshop	• Separate BREATHE content into smaller modules delivered over time<br>• Trial additional electronic resources and reminders outside the workshop setting.<br>• Revise BREATHE materials to specifically address how to cope with job demands that promote burnout.<br>• Target interventions to MHWs experiencing higher levels of burnout.<br>• Randomised control groups and longer follow-up periods<br>• Ensure acceptability and feasibility with racial and ethnic minority groups
Expressive arts	• Recruit participants from a variety of settings to examine individual differences

Intervention	Recommendations/ Questions for future research
	• Use a wider variety of research methods to explore impact<br>• Investigate the precise effects of group drumming on factors associated with burnout
Peer support/ relationships	• Longitudinal designs with follow-up to explore longevity of effects.<br>• Compare: online and face-to-face peer groups; hybrid models that include both online and face-to-face components.<br>• Focus on both process and outcome measures<br>• Control groups and larger samples.<br>• Recruit participants with higher levels of emotional exhaustion to explore impact on this aspect of burnout more specifically
Balint-like group<br>Training in other therapeutic interventions (CBT; psychosis/BPD interventions).	• Larger sample sizes with more statistical power<br>• More rigorous experimental designs<br>• Does training result in improved patient outcomes?<br>• Will results generalise to other mental health providers?<br>• Do reductions in burnout for MHWs attending training sustain over time?
Wellness/stress reduction	• Recruit homogeneous samples or else stratify more varied samples by job classification<br>• Larger samples, randomization to groups<br>• Explore differences between single and partnered MHWs, including experience and/or frequency of touch in their lives.
	•

Note. ACT = acceptance and commitment therapy; BREATHE = Burnout Reduction: Enhanced Awareness, Tools, Hand-outs, and Education; CF = compassion fatigue; CITs = counsellors in training; DBT= dialectical behaviour therapy; MBIs= mindfulness-based interventions; MHWs= mental health workers; PSI = psychosocial interventions; STS = secondary traumatic stress

This is a provisional file, not the final typeset article

Figure 3

Click here to access/download;Figure;Figure 3 TIFF.tif

		Primary	Secondary	Tertiary
**Individual**	Personal practice		• Wellness/stress reduction<br>• Mindfulness<br>• ACT<br>• Compassion enhancement<br>• Expressive arts<br>• Aspects of the BREATHE intervention and resiliency workshops	
	Awareness raising		• EBS prevention<br>• Aspects of the BREATHE intervention and resiliency workshops	
	Role training		• Training in a therapeutic modality (DBT; PSI; CBT)	
**Team**			• Supervision<br>• Peer groups/ relationships<br>• Balint-like group	
**Organisational**		• Primary nursing		

Figure 2: PRSIMA FlowChart

Click here to access/download;Figure;Figure 2 TIFF.tif

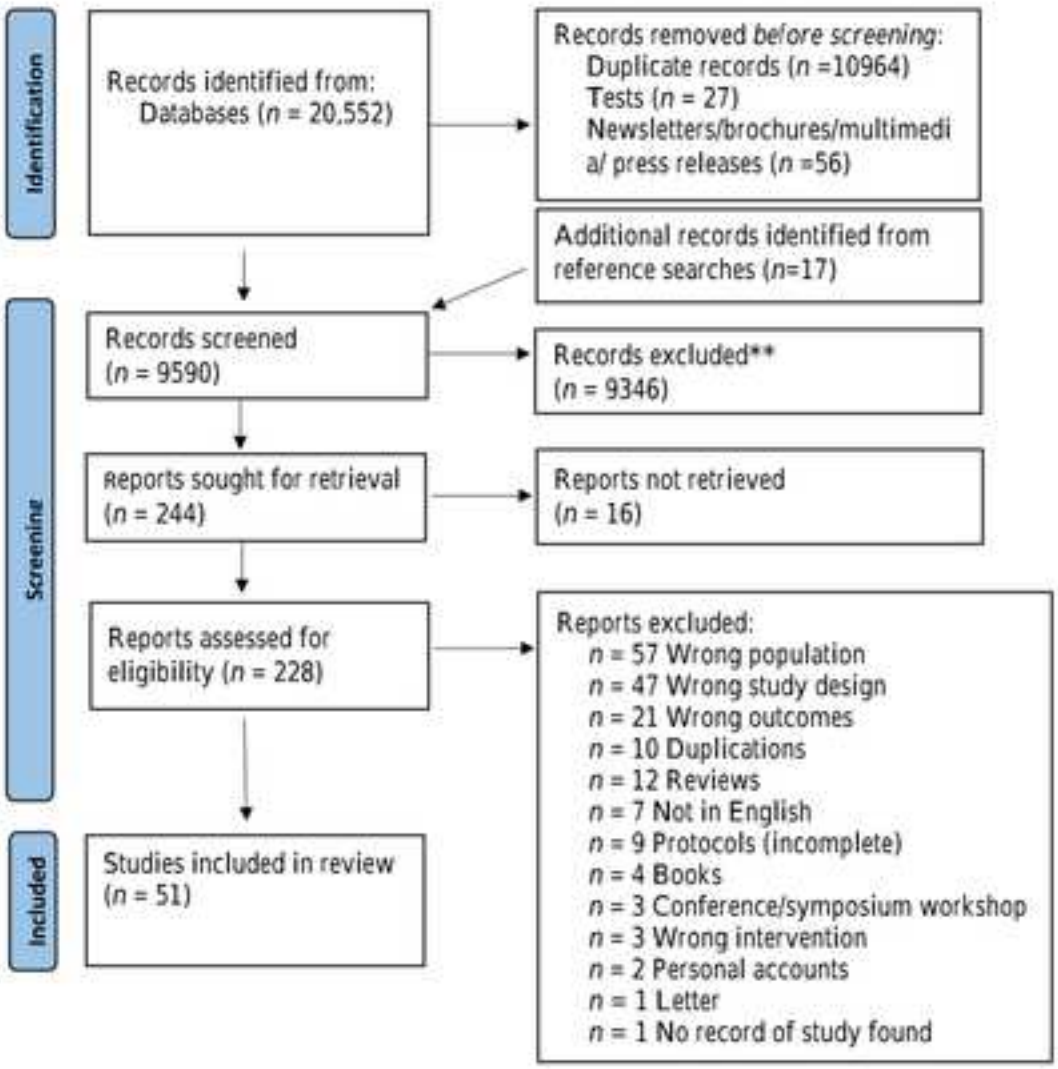

Figure 1

Click here to access/download;Figure;Figure 1 TIFF.tif

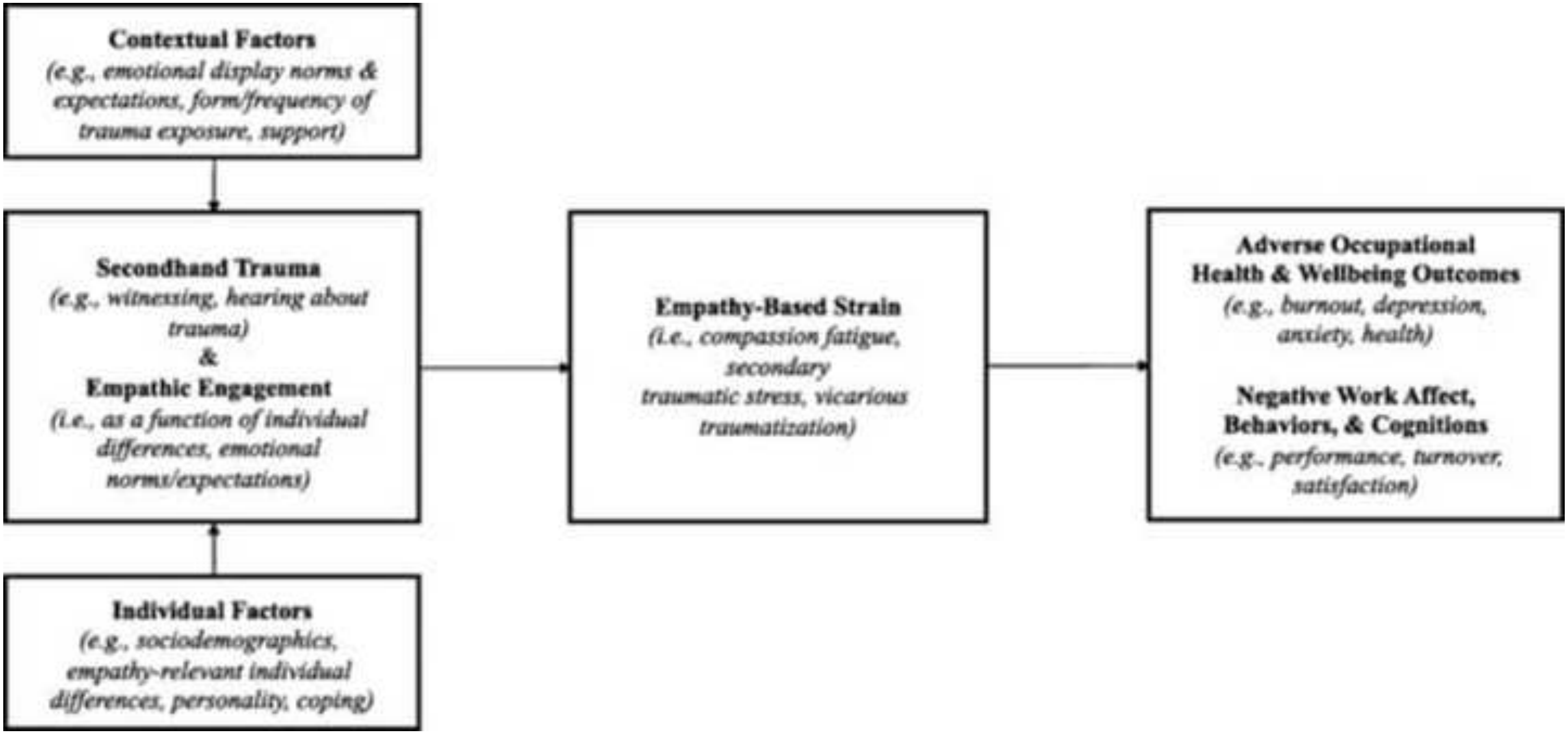

Click here to access/download
Supporting Information
PRISMA Chart.docx
