# Supplementary material for: Interventions to address empathy-based stress in mental health workers: A scoping review and research agenda
Source: PLoS One. 2024 Dec 5;19(12):e0306757. doi: 10.1371/journal.pone.0306757 (PMC11620613; doi:10.1371/journal.pone.0306757)
Supplement: Attachment — Submitted filename: Report of review the paper (PONE-D-23-33814).docx [file pone.0306757.s003.docx]

**Report of review the paper (PONE-D-23-33814)**

**Dear editor/PLOS ONE**

**Thank you for giving me this chance to revise this valuable paper with valuable variable that can affect healthcare worker specially nurses and physician and can lead to increase turnover**

**The title of the research** is not clear , it is not intervention it is scoping reviews

**The abstract** :too long , the four construct must be showed in introduction of abstract

**Introduction**

- what already known and what is added by your research
- very long you can start from the line no( 54) and summarize the paragraph start with line no:77-89
- figure no 1 not included in the paper.
- the other construct of the conceptual model need to be related to MHW as CF(line no 102:112)
- the significant of the study need to be clear and concise it was scattered in the introduction.
- the gab of knowledge /this research need to be identified.
- The conceptual frame work need to be more clear and identify each construct with adequate references

**Methods**

- Scoping reviews tend to focus on the nature, volume, or characteristics of studies rather than on the synthesis of published data. In health care it would prefere to uses systemic to decrease bias and chose best in class research.
- The research question need to be added
- The protocol of scoping reviews need to be addeding
- The steps of scope reviews must be covered from identifying research question till Collating, Summarizing, and Reporting the Results with clear explanation of each step

- **Results :**
- In table 4 the scope review not include each of VC also more than 80% of research concentrate on **burnout** although the research conclude in the introduction it is results more than construct.
- Characteristics of Included Studies need to be visualized in no & percent
- Trend analysis used to present the changing frequency of research over the past 20 years, based on the aforementioned classification criteria need to be added

**Intervention**

- This part are very long need to be shorten and focus on the most affected intervention that can decrease EBS or burnout.
- The intervention must be declared to what extent it support or against or answer the authors research questions and cover the proposed protocol
- The balance between different interventions was lost in this section . it focuses only on three interventions while the other intervention reference was very little

**Discussion**

- It isy long and need to be concise
- Although I am not native speakers but the paper as all need to be review by English editors

**References :**

- More than 50 refrence rang rom 1953 to2009
