# Supplementary material for: Interventions to address empathy-based stress in mental health workers: A scoping review and research agenda
Source: PLoS One. 2024 Dec 5;19(12):e0306757. doi: 10.1371/journal.pone.0306757 (PMC11620613; doi:10.1371/journal.pone.0306757)
Supplement: Attachment — Submitted filename: REVIEW REPORT.docx [file pone.0306757.s004.docx]

Thank you for the opportunity to read this interesting and detailed article. I provide my comments/suggestions which the authors may find useful for improving the overall quality of the paper.

1. I think the use of questions and tables to introduce this article does not make the Introduction stronger. Authors may reorganize this section. In its current form is more like giving out information to readers, rather than presenting a problem, telling what has been done about it, and what needs to be done which you are going to do.
2. In terms of the study justification, I think the authors may go deeper. For instance, one of the cited studies ( Bercier and Maynard, 2014) to justify the current study is not even in the reference list for confirming what has been stated in the article. Also, the authors state that they included studies with at least 50% of the participants being mental health workers. How is their study different from earlier studies (such as Conversano et al., 2020) that combined mental health workers and other participants?
3. Authors may present the study objectives more concisely.
4. Same with the methods, especially from line 146 to 167. This could be reorganized to make it more concise.
5. The in-text citations are not conforming to the journal’s referencing style. Also, some of the in-text citations cannot be found in the reference list. An important example is Bercier and Maynard (2014) cited in line 117 to justify the need for the present study.
6. Authors may be consistent with the use of "Whilst" and "While"
