# Supplementary material for: Interventions to address empathy-based stress in mental health workers: A scoping review and research agenda
Source: PLoS One. 2024 Dec 5;19(12):e0306757. doi: 10.1371/journal.pone.0306757 (PMC11620613; doi:10.1371/journal.pone.0306757)
Supplement: Attachment — Submitted filename: Response to Reviewers.docx [file pone.0306757.s006.docx]

**Dr Hannah May**

**Department of Psychology**

**University of Bath, Claverton Down,**

**Bath, UK, BA2 4BA**

**Email: hannahrebeccamay@hotmail.co.uk**

**Prof. Ebtsam Abou Hashish**

**Academic Editor**

**PLOSONE**

15.03.2024

Dear Professor Abou Hashish

Thank you for the opportunity to revise our manuscript: ‘Interventions to Address Empathy-Based Stress in Mental Health Workers: A Scoping Review and Research Agenda’ for publication in PLOSONE.

We would like to thank you and the panel of reviewers for your time and comprehensive feedback and suggestions that have enabled up to enhance the quality and impact of our manuscript. I can confirm that each of the reviewers’ comments have been responded to as per below. All of the revisions as outlined below have also been made to the manuscript and are highlighted in yellow within the manuscript. Where we wished to draw a reviewer’s attention to a piece of text relevant to their comment, we have highlighted in yellow.

We note that there was one suggestion (first comment from reviewer 3) to make the research agenda an appendix to the review. We are happy for this to be done if you choose, however have not made this change in the corrected manuscript as yet as this seemed to be an editorial decision.

Thank you for your consideration of this manuscript. We look forward to hearing from you.

Sincerely,

Dr Hannah May, on behalf of myself and co-authors

ACADEMIC EDITOR Feedback

Overall Paper Structure and Content:
**1. Title and Abstract:** Clarify that the paper is a scoping review rather than focusing on interventions directly. Ensure the abstract introduces the four main constructs and succinctly highlights the research's unique contributions.

*Response: Thank you for this comment. We have amended the abstract (pg 2) to more clearly identify the paper as a scoping review and to emphasise its unique contribution. The paper is also identified as a scoping review within its title. We have also added reference to the four main constructs of EBS to the abstract.*

**2. Introduction:** Condense the introduction, starting from line 54, and emphasize the gap in knowledge the research addresses. Create a clearer link between the conceptual model and mental health workers (MHW).

*Response: Thank you for this comment. We have amended the introduction to more clearly emphasise the relevance of the EBS model to mental health workers (pg 4). We have also condensed the introduction as requested (pgs 2-8).*

**3. Methods**: Enhance clarity on scoping review protocols, from identifying the research question to reporting results. Explicitly state the research question and protocol used.

*Response: Thank you for this comment. The research question and associated aims are given in the ‘Current Review and Aims’ section (pg 7), and copied below for ease of reference:*

*““What interventions exist to prevent or treat EBS in MHWs?”. Using systematic scoping methodology, we aimed to answer this question via the following objectives:*

*1)	To assess and summarise the available interventions for preventing or treating EBS in MHWs*

*2)	To ascertain the theoretical underpinnings and assumptions of these interventions*

*3)	To assess how EBS has been measured in these studies*

*4)	To make recommendations for future research into the treatment and prevention of EBS in	MHWs”*

*As described in the methods section, the review was conducted in accordance with the Joanna Briggs Scoping Review Framework (Peters et al., 2017) and written up in line with the Prisma Extension for Scoping Reviews (Tricco et al., 2018). As described in line 168, An a priori protocol was developed and registered on Open Science Framework and is available at the following link: https://osf.io/b7kcr/*

**4.Results:** Revise Table 4 to cover all variables of interest. Visualize characteristics of included studies in percentages and incorporate trend analysis over the past 20 years.

*Response: Thank you for this suggestion. We have reported the characteristics of the included studies in accordance with the Joanna Briggs Scoping Review Framework (Peters et al., 2017) and the Prisma Extension for Scoping Reviews (Tricco et al., 2018) In line with these, Table 4 of the scoping review is not intended to provide further analysis but to present the characteristics of included studies which are relevant to the research question. Table 4 in the manuscript accordingly presents this data. The distribution of included studies by year of publication is not relevant to our research questions. For example, the Joanna Briggs Scoping Review Framework (Peters et al., 2017) instructs:*

*“In a scoping review, the results may be presented as a ‘map’ of the data in a logical, diagrammatic, or tabular form, and/or in a descriptive format that aligns to the objective/s and scope of the review. The tables and charts may show results as: distribution of studies by year or period of publication (depends on each case), country of origin, area of intervention (clinical, policy, educational, etc.), and research methods. It is up to the reviewers to decide which would most rationally and clearly illustrate the nature of the results in terms of the objective/s and question/s of the review. A summary of the results should logically describe the aims or purposes of the included articles, the concepts or approaches adopted in each, and the results that relate to the review question/s.”*

**5. Intervention**: Streamline this section, focusing on the most impactful interventions. Provide clarity on how these interventions align with the research questions and proposed protocol. Balance coverage of different interventions.

*Response: Thank you for this comment. The section reporting on interventions to reduce/prevent EBS (starting on pg 28) is part of the results section, and is therefore aligned with the first aim of the research question: “ To assess and* *summarise the available interventions for preventing or treating EBS in MHWs”.*

*We have retitled this subsection of the results section “Summary of Available Interventions for Preventing or Treating EBS in MHWs” to more clearly orient the reader to the purpose of this section and link it back to the relevant research aim.*

*We have made edits (pg 28-39) in order to enhance clarity and readability of this section. However, identifying the most impactful interventions was not one of our aims, nor is it compatible with the purpose of a scoping review. As pointed out on pg 46:*

*“It is beyond the remit of a scoping review to assess and compare effectiveness of interventions. However, it is worth noting that even superficial observations about the relative outcomes of different approaches were difficult to make due to: the number of different interventions used; the multi-component nature of many of the interventions and the tendency for studies to report changes across multiple subscales of EBS measures.”*

**6. Discussion:** Condense the discussion for conciseness. Also, ensure the paper undergoes thorough English editing.

*Response: thank you for this comment. We have condensed the discussion for conciseness (pgs 39-46) and reviewed English editing for the whole manuscript.*

**7. References**: Update and expand the reference list to cover a wider time span and ensure conformity to the journal's referencing style.

*Response: Thank you for this comment. As described on pg 9, the time span of the review was based on the history of the relevant constructs. As identified by Schaufeli et al (2009), the term burnout (the oldest of the constructs) was first used in 1970 and therefore this was the starting range for searches. We have reviewed the referencing and ensured conformity to the journals referencing style.*

**Addressing Specific Reviewer Comments: in addition to detailed comments of reviewer 1. consider the following**

1. Reviewer 2: Clarify the study's unique aspects compared to previous research. Ensure consistency in referencing style.

*Response: thank you for this comment. We have reviewed the introduction in line with the comments regarding the narrative as suggested here, and also have edited for clarity and conciseness throughout as suggested by multiple reviewers. The references have been reviewed for completeness and for adherence to the journal’s style. See responses to Reviewer 2 below for more detail.*

2. Reviewer 3: Enhance the readability of the research agenda section.

*Response: thank you for this comment. The research agenda has been edited for readability*

3. Reviewer 4: Address the conflict between healthcare organizations and providers, emphasizing structural interventions. Consider the U.S. healthcare perspective and its impact on the paper's content.

*Response: thank you for this comment. It is an interesting observation, however, the available evidence suggests that improving staff engagement and satisfaction leads to precisely the outcomes healthcare organisations seek. Data from the largest annual staff survey in healthcare show that improving engagement and satisfaction leads to better care quality, patient satisfaction, financial performance and staff retention. We have added this information, with references, to page 6.*

4. Reviewer 5: Organize the content for better flow, specifically around outlining intervention levels early, providing clear linkages between findings and sources, and expanding on prevention-focused research efforts.

*Response: Thank you for this comment. We have edited for better flow, outlined intervention levels by Tetrick & Quick where they first appear and expanded on prevention-focused research ideas as suggested. Please see individual responses below for further details.*

**General Recommendations:**

Clarity and Conciseness: Ensure each section is concise, coherent, and directly addresses the objectives and contributions of the paper.

*Response: Thank you for this comment. We have edited the paper for conciseness and clarity based on feedback from multiple reviewers.*

Consistency and Detail: Maintain consistency in referencing style and detail the unique contributions of the study compared to existing literature.

*Response: Thank you for this comment. As noted above, we have amended lines the abstract (pg 2) to more clearly identify the paper as a scoping review and to emphasise its unique contribution. We have also corrected referencing style to be in line with journal style.*

Addressing Gaps: Highlight the gap in knowledge that the paper addresses and ensure the conceptual model directly relates to mental health workers.

Response: Thank you for this comment. As noted above, we have amended the abstract (pg 2) to more clearly identify the paper as a scoping review and to emphasise its unique contribution. We have also amended the introduction more clearly emphasise the relevance of the EBS model to mental health workers (pg 4).

Visual Representation: Use tables and figures effectively to illustrate key points, characteristics of studies, and trends in the field.

*Response: Thank you for this comment. As noted above, we have not produced a visual trend analysis as we have reported the characteristics of the included studies in accordance with guidance on conducting and reporting systematic scoping reviews. In line with these, the results section of the scoping review is not intended to provide further analysis but to present the characteristics of included studies which are relevant to the research question. Table 4 in the manuscript accordingly presents this data. The distribution of included studies by year of publication is not relevant to our research questions. For example, the Joanna Briggs Scoping Review Framework (Peters et al., 2017) instructs:*

*“In a scoping review, the results may be presented as a ‘map’ of the data in a logical, diagrammatic, or tabular form, and/or in a descriptive format that aligns to the objective/s and scope of the review. The tables and charts may show results as: distribution of studies by year or period of publication (depends on each case), country of origin, area of intervention (clinical, policy, educational, etc.), and research methods. It is up to the reviewers to decide which would most rationally and clearly illustrate the nature of the results in terms of the objective/s and question/s of the review. A summary of the results should logically describe the aims or purposes of the included articles, the concepts or approaches adopted in each, and the results that relate to the review question/s.”*

Addressing these areas should significantly improve the paper's quality and address the concerns raised by the reviewers. It may require restructuring, condensing, and enhancing clarity throughout the document. Additionally, consider seeking professional assistance for language editing to ensure grammatical correctness and overall readability.

*Response: Thank you for this comment. The manuscript has been reviewed for grammatical accuracy.*

**Reviewer 1**

**The title of the research** is not clear , it is not intervention it is scoping reviews

Response: Thank you for this comment. We have amended lines the abstract (pg 2) to more clearly identify the paper as a scoping review and to emphasise its unique contribution. The paper is also identified as a scoping review within its title.

**The abstract** :too long , the four construct must be showed in introduction of abstract

*Response: Thank you for this comment. We have included reference to the four constructs of EBS within the abstract (page 2). PLOSONE journal submission guidance states that abstracts should not exceed 300 words. Our abstract is 228 words, including both this addition and minor additions requested by reviewers in relation to separate comments.*

**Introduction**

- what already known and what is added by your research

*Response: Thank you for this comment. Thie aim of this reviews were, as summarized in the ‘current review and aims section’ (pg 7):*

1) *“To assess and summarise the available interventions for preventing or treating EBS in MHWs*
2) *To ascertain the theoretical underpinnings and assumptions of these interventions*
3) *To assess how EBS has been measured in these studies*
4) *To make recommendations for future research into the treatment and prevention of EBS in	MHWs”*

*Therefore, the knowledge that is added is in synthesizing the findings of the scoping review . The outcome of which is reported on in the results section and elaborated on in the discussion.*

- very long you can start from the line no( 54) and summarize the paragraph start with line no:77-89

*Response: Thank you for this comment. We have edited the paper for conciseness and clarity throughout based on feedback from multiple reviewers, with particular focus on the introduction*.

- figure no 1 not included in the paper.

*Response: thank you for this comment. Figure 1 as uploaded in the submission is a diagram showing the EBS model, reproduced with permission by the authors of the Rauvola et al (2019) paper where this first appeared. The traditional ‘figure 1’ i.e. the PRISMA flow chart is included as Figure 2.*

- the other construct of the conceptual model need to be related to MHW as CF(line no 102:112)

*Response: Thank you for this comment. We are unsure what exactly is being requested here. The review (Turgoose & Maddox, 2017) referenced in the section indicated by the reviewer does specifically reference compassion fatigue as an example, however the subsequent references refer to EBS more broadly. In addition, whilst we have made reference to individual constructs (compassion fatigue, burnout etc) where appropriate to the specific paper being cited, the purpose of using the umbrella term EBS is to highlight the high degree of conceptual overlap and inter-relatedness of these concepts. The rationale for doing this and the EBS model is described in the introduction.*

- the significant of the study need to be clear and concise it was scattered in the introduction.
- the gab of knowledge /this research need to be identified.

*Response: Thank you for these two comments, which we are responding to as one as they seem to make overlapping points. As above, we have edited the paper for conciseness and clarity with particular focus on the introduction. We would also highlight that the “Current Review and Aims” (pg 7) summarises the unique contribution of the review and the gap of knowledge it is addressing.*

- The conceptual frame work need to be more clear and identify each construct with adequate references

*Response: Thank you for this comment, details of each construct along with references are outlined in Table 1.*

**Methods**

- Scoping reviews tend to focus on the nature, volume, or characteristics of studies rather than on the synthesis of published data. In health care it would prefere to uses systemic to decrease bias and chose best in class research.

*Response: Thank you for this comment. Our review question (pg 7) was “What interventions exist to prevent or treat EBS in MHWs?”. Using systematic scoping methodology, we aimed to answer this question via the following objectives:*

1. *To assess and summarise the available interventions for preventing or treating EBS in MHWs*
2. *To ascertain the theoretical underpinnings and assumptions of these interventions*
3. *To assess how EBS has been measured in these studies*
4. *To make recommendations for future research into the treatment and prevention of EBS in	MHWs”*

*In accordance with these aims, a scoping methodology was the most appropriate as our intention was not to assess relative effectiveness of interventions but to understand what interventions were being used, the theoretical reationale for them and how EBS was measured. Furthermore, whilst a scoping review does not compare effectiveness it is still thoroughly systematic in its approach and involves an a priori protocol and a methodical, exhaustive, and replicable search strategy. The rationale for conducting a scoping review is described in some detail in the Methods section.*

- The research question need to be added

*Response: Thank you for this comment. As in the response to the comment above, our review question was “What interventions exist to prevent or treat EBS in MHWs?” and this plus the associated aims are described in lines in the ‘current review and aims sectiion’ (pg 7).*

- The protocol of scoping reviews need to be addeding

*Response: thank you for this comment. As reported in the method section (pg 9), an a priori protocol was developed and registered on Open Science Framework: https://osf.io/b7kcr/*

- The steps of scope reviews must be covered from identifying research question till Collating, Summarizing, and Reporting the Results with clear explanation of each step

*Response: thank you for this comment. We respectfully suggest that the steps of the review are already outlined in the methods section (pg 9-13), which describe a priori protocol development; electronic searches and search term selection, study selection and charting of data (all clearly identified with subheadings)*

- **Results :**
- In table 4 the scope review not include each of VC also more than 80% of research concentrate on **burnout** although the research conclude in the introduction it is results more than construct.

*Response: Thank you for this comment. This is an interesting observation, though we are not sure we fully understand what is being suggested here. Table 4 reports on characteristics of included studies as per our systematic search strategy. It is correct that a large proportion of studies did target burnout and none of them targeted vicarious trauma. The preponderance of burnout studies commented on in the results section (pg 15) and in the discussion (pg 40) with reference to the EBS model (i.e., in EBS model burnout is the adverse outcome of empathy-based strain)*

- Characteristics of Included Studies need to be visualized in no & percent

*Response: thank you for this comment. We appreciate visual presentation of data can be useful, however given the large amount of included studies, the heterogeneity of those studies and the breadth of data included in Table 4 we do not feel this would be a useful addition to the paper. Furthermore it would again increase the bulk of the paper amidst multiple requests of reviewers for it to be streamlined.*

- Trend analysis used to present the changing frequency of research over the past 20 years, based on the aforementioned classification criteria need to be added

*Response: Thank you for this comment. However, we have reported the characteristics of the included studies in accordance with the Joanna Briggs Scoping Review Framework (Peters et al., 2017) and the Prisma Extension for Scoping Reviews (Tricco et al., 2018). In line with these, the results section of the scoping review is not intended to provide further analysis but to present the characteristics of included studies which are relevant to the research question. Table 4 in the manuscript accordingly presents this data. The distribution of included studies by year of publication is not relevant to our research questions. For example, the Joanna Briggs Scoping Review Framework (Peters et al., 2017) instructs:*

*“In a scoping review, the results may be presented as a ‘map’ of the data in a logical, diagrammatic, or tabular form, and/or in a descriptive format that aligns to the objective/s and scope of the review. The tables and charts may show results as: distribution of studies by year or period of publication (depends on each case), country of origin, area of intervention (clinical, policy, educational, etc.), and research methods. It is up to the reviewers to decide which would most rationally and clearly illustrate the nature of the results in terms of the objective/s and question/s of the review. A summary of the results should logically describe the aims or purposes of the included articles, the concepts or approaches adopted in each, and the results that relate to the review question/s.”*

**Intervention**

- This part are very long need to be shorten and focus on the most affected intervention that can decrease EBS or burnout.

*Response: thank you for this comment. As above, we have changed the subheading of this section to “Summary of Available Interventions for Preventing or Treating EBS in MHWs****”*** *to more clearly demonstrate that it links to the first aim of our review question- “1.	To assess and summarise the available interventions for preventing or treating EBS in MHWs”. In line with our aims and with the purpose of a systematic scoping review, we were not intending to compare efficacy of different interventions. We have edited the whole manuscript for conciseness and clarity based on feedback from multiple reviewers, including in this section.*

- The intervention must be declared to what extent it support or against or answer the authors research questions and cover the proposed protocol

*Response: Thank you for this comment. We are not sure we fully understand what is being requested here, as we were not making predictions with our review questions/aims, but setting intentions to describe the state of the literature with reference to: available interventions for treating/preventing EBS in MHWS, the theoretical underpinnings of interventions, and the measurement of EBS within these studies. This we have done in the results and discussion sections.*

- The balance between different interventions was lost in this section . it focuses only on three interventions while the other intervention reference was very little

*Response: Thank you for this comment. We appreciate it is challenging to summarise such a varied and heterogeneous group of interventions in a balanced way but have done our best to cover all interventions whilst grouping them in a logical manner, as acknowledged on page 30 : “Given the diverse range of interventions described, for clarity, they have been grouped according to the main intervention components as described in the studies themselves (Table 6). It is acknowledged some of the more integrative interventions contained various elements and could potentially be categorised multiple ways.” Table 6 describes 12 types of intervention.*

**Discussion**

- It isy long and need to be concise

*Response: thank you for this comment. We have edited the whole manuscript for conciseness and clarity based on feedback from multiple reviewers, including in this section.*

- Although I am not native speakers but the paper as all need to be review by English editors

*Thank you for this comment. We cannot comment on the nationality of other reviewers however have edited and reviewed the manuscript for clarity and readability.*

**References :**

- More than 50 refrence rang rom 1953 to2009

*Response: thank you for this comment. We are unsure if this is an observation or a request to make changes. As described in lines 173-174, the time span of the review was based on the history of the relevant constructs. As identified by Schaufeli et al (2009), the term burnout (the oldest of the constructs) was first used in 1970 and therefore this was the starting range for searches (described on pg 9)*

**Reviewer 2**

Thank you for the opportunity to read this interesting and detailed article. I provide my comments/suggestions which the authors may find useful for improving the overall quality of the paper.

*Response: thank you for the positive feedback. See below for responses to individual suggestions.*

1. I think the use of questions and tables to introduce this article does not make the Introduction stronger. Authors may reorganize this section. In its current form is more like giving out information to readers, rather than presenting a problem, telling what has been done about it, and what needs to be done which you are going to do.

*Response: Thank you for this comment. Due to the need to introduce a large amount of conceptual information to set the scene for this review, we appreciate the introduction is longer than some. We hoped the use of tables and question subheadings would assist the reader in digesting this, however we are happy to remove these and signpost the reader to supplementary material if the reviewers feel this would enhance readability. We have reviewed the introduction in line with the comments regarding the narrative as suggested here, and also have edited for clarity and conciseness throughout as suggested by multiple reviewers*.

2. In terms of the study justification, I think the authors may go deeper. For instance, one of the cited studies (Bercier and Maynard, 2014) to justify the current study is not even in the reference list for confirming what has been stated in the article. Also, the authors state that they included studies with at least 50% of the participants being mental health workers. How is their study different from earlier studies (such as Conversano et al., 2020) that combined mental health workers and other participants?

*Response: thank you for this comment. We apologise for the typographical error whereby Bercier and Maynard (2014) was absent for the reference list and have corrected this. In terms of the difference between this review and the review by Conversano et al (2020), the aims of the authors in Conversano et al were to give an “overview on recent literature about mindfulness and compassion characteristics of HCPs, while exploring the effectiveness of techniques, involving the two aspects, such as MBSR or mindfulness intervention and compassion fatigue-related programs." Therefore not only were they focusing on a broader category of participants than the current review, they were also specifically looking at characteristics of those participants (compassion and mindfulness) in addition to exploring compassion fatigue interventions solely using these aspects. We have added a brief note explaining this difference on pg 7.*

3. Authors may present the study objectives more concisely.

Response: thank you for this comment. We have duly reviewed the objectives (copied below) however have struggled to see how to edit these further from their current form without losing meaning and specificity. We have however edited the section in which the objectives appear (Current Review and Aims, pg 7) for conciseness and clarity.

“What interventions exist to prevent or treat EBS in MHWs?”. Using systematic scoping methodology, we aimed to answer this question via the following objectives:

1)	To assess and summarise the available interventions for preventing or treating EBS in MHWs

2)	To ascertain the theoretical underpinnings and assumptions of these interventions

3)	To assess how EBS has been measured in these studies

4)	To make recommendations for future research into the treatment and prevention of EBS in	MHWs”

4. Same with the methods, especially from line 146 to 167. This could be reorganized to make it more concise.

*Response: thank you for this comment. This section, along with the whole manuscript, has been edited for conciseness and clarity.*

5. The in-text citations are not conforming to the journal’s referencing style. Also, some of the in-text citations cannot be found in the reference list. An important example is Bercier and Maynard (2014) cited in line 117 to justify the need for the present study.

*Response: thank you for this comment and our apologies for the omission of this reference which has now been added. The references have been reviewed for completeness and for adherence to the journal’s style.*

6. Authors may be consistent with the use of "Whilst" and "While"

*Response: thank you for this comment. We have removed “while” and been consistent with use of “whilst”.*

**Reviewer 3**

Thanks for an interesting and informative review. Would be helpful to detail the data synthesis done and if possible make the research agenda appendix to the review to reduce length and aid readability. Other comments attached in the reviewed document

*Response: thank you for this comment and for the positive feedback. We have added a brief reference to the use of narrative synthesis at the start of the results section in line 228. We would be happy to remove the research agenda to the appendix if reviewers feel this would enhance readability.*

**Reviewer 4**

Thank you very much for the opportunity to review this manuscript on a scoping review and research agenda regarding interventions that target empathy-based stress (EBS) in mental health workers (MHWs). I found the review steps and presented information to be very comprehensive and clear, and I particularly appreciated the authors’ (i) acknowledgement of categorizations of findings not necessarily being mutually exclusive (e.g., Lines 340-342) and (ii) descriptions of specific inclusion/exclusion decisions that required additional thought (e.g., 328-339).

*Response: thank you very much for these comments and the positive feedback, it is much appreciated.*

My suggestions below are mostly to enhance (i) flow and clarity of the manuscript’s covered concepts and (ii) linkages between the reported findings and the articles from which they originate.

1. Please consider moving the information in Lines 136-144 (which do not seem to be MHW-specific) to be earlier in the Introduction section, perhaps around Lines 54-55 that describe why EBS deserves attention (i.e., before Line 90, which starts to describe the rationale for studying EBS in MHWs specifically).

*Response: thank you for this comment. We have edited the introduction in response to feedback from multiple reviewers. Part of the section in question has been removed for conciseness, and part of it (describing negative outcomes for healthcare staff arising from burnout) has been repurposed to illustrate why organisations (not just individual MHWs) should care about EBS (pg6-7).*

2. Please consider outlining the three intervention levels of Tetrick and Quick (2011) when they are first mentioned in the manuscript (Lines 231-232), rather than later on in Lines 384-392.

*Response: thank you for this comment. We appreciate the logic of the request, however the full descriptions of the 3 intervention levels from Tetrick and Quick (2011) are most relevant at the point where they currently appear in the manuscript i.e. in the ‘Theoretical Underpinnings and Assumptions’ section where the route of intervention of included studies is being considered. As noted by reviewer 4 this is approximately 150 lines later than where they are first mentioned and therefore our concern is that moving them earlier would reduce readability for the section where they are most relevant. In compromise, we have added the names of the 3 intervention levels in brackets at the first mention and left the detailed descriptions in their original place.*

3. Please consider previewing around Lines 647-650 that, following the descriptions of current issues and suggested solutions in the Research Agenda section, a list of summary recommendations will be provided.

*Response: thank you for this comment. We have added a line previewing this as requested (pg 47)*

4. Please consider making clearer which exact reviewed articles are associated with each different type of (i) broad categories of interventions, (ii) routes of intervention, (iii) measures, and (iv) recommendations for future research, which the manuscript describes very usefully in Table 6, the “Route of Intervention” section (starting on Line 365 and Figure 3), Appendix C, and Appendix D, respectively.

*Response: thank you for this comment. In line with the review aims our intention was to describe broad patterns in the evidence and answer specific questions about the knowledge base available rather than comparing individual studies.*

5. Please consider expanding, around Lines 754-758, on some specific examples of potential ways in which future research efforts can reflect a focus on prevention.

*Response: thank you for this comment. We have added some specific suggestions for research to investigate prevention of EBS to the research agenda (pg 51-52).*

6. Please consider clarifying, throughout the manuscript where the search dates are mentioned, that the updated search included articles up until September 2022 (since Line 183 mentions that the updated search was conducted in September 2022), rather than just noting “2022,” which indicates that articles from all months of 2022 were included in the review.

*Response: thank you for this comment. We have clarified this.*

**Reviewer 5**

This paper reflects an enormous amount of work that summarizes interventions to address empathy-based stress (EBS) in mental health care workers. It includes the definitions of EBS and the details of the interventions that have been studied to reduce it. This summary will be of value to future researchers.

*Response: many thanks for this comment and the recognition of the work and effort taken to produce the review.*
I believe that the paper's main shortcoming is its failure to address the inherent conflict between health care organizations and the health care providers they employ. For example, in the U.S. there are numerous newspaper articles about health care organizations seeking to hire as few staff as they can get away with and health care workers seeking to unionize and/or going on strike to assert their rights to reasonable work conditions.

It's naive to assume health care organizations are motivated to make such structural interventions such as reducing workload, even if in the long run it would benefit them to do so. This paper needs to address conflicts between the interests of health care workers and the economic drivers that cause health care organizations to deliver care as inexpensively as possible.

*Response: thank you for this comment. It is an interesting observation, however, the available* *evidence suggests that improving staff engagement and satisfaction leads to precisely the outcomes healthcare organisations seek. Data from the largest annual staff survey in healthcare show that improving engagement and satisfaction leads to better care quality, patient satisfaction, financial performance and staff retention. We have added this information, with references, to pgs 6-7.*

Additional Journal requirements:

When submitting your revision, we need you to address these additional requirements.

1. Please ensure that your manuscript meets PLOS ONE's style requirements, including those for file naming. The PLOS ONE style templates can be found at

<https://ddec1-0-en-ctp.trendmicro.com:443/wis/clicktime/v1/query?url=https%3a%2f%2fjournals.plos.org%2fplosone%2fs%2ffile%3fid%3dwjVg%2fPLOSOne%5fformatting%5fsample%5fmain%5fbody.pdf&umid=1225bc8e-2e69-491e-bcb5-366859b053fc&auth=5a48ddabf21f7246250a6ac00727f7875e94cad3-5c2f7c7b32ddee506d986a11922988a79d13c7da>

and

<https://ddec1-0-en-ctp.trendmicro.com:443/wis/clicktime/v1/query?url=https%3a%2f%2fjournals.plos.org%2fplosone%2fs%2ffile%3fid%3dba62%2fPLOSOne%5fformatting%5fsample%5ftitle%5fauthors%5faffiliations.pdf&umid=1225bc8e-2e69-491e-bcb5-366859b053fc&auth=5a48ddabf21f7246250a6ac00727f7875e94cad3-b6dab4e6258073e5f49d229eadf703b328592f14>

*Response: thank you for this comment. Headings, figure titles and supporting information have been amended accordingly.*

2. Note from Emily Chenette, Editor in Chief of PLOS ONE, and Iain Hrynaszkiewicz, Director of Open Research Solutions at PLOS: Did you know that depositing data in a repository is associated with up to a 25% citation advantage (<https://ddec1-0-en-ctp.trendmicro.com:443/wis/clicktime/v1/query?url=https%3a%2f%2fdoi.org%2f10.1371%2fjournal.pone.0230416%29%3f&umid=1225bc8e-2e69-491e-bcb5-366859b053fc&auth=5a48ddabf21f7246250a6ac00727f7875e94cad3-02c697a384fce9221f875f6755cf2d93d0a8be16> If you’ve not already done so, consider depositing your raw data in a repository to ensure your work is read, appreciated and cited by the largest possible audience. You’ll also earn an Accessible Data icon on your published paper if you deposit your data in any participating repository (<https://ddec1-0-en-ctp.trendmicro.com:443/wis/clicktime/v1/query?url=https%3a%2f%2fplos.org%2fopen%2dscience%2fopen%2ddata%2f%23accessible%2ddata&umid=1225bc8e-2e69-491e-bcb5-366859b053fc&auth=5a48ddabf21f7246250a6ac00727f7875e94cad3-125f04cf3697bbd2c30cc09bf465869e40888a35>).

*Response: thank you for this comment. We do not have any data to deposit.*

3. PLOS requires an ORCID iD for the corresponding author in Editorial Manager on papers submitted after December 6th, 2016. Please ensure that you have an ORCID iD and that it is validated in Editorial Manager. To do this, go to ‘Update my Information’ (in the upper left-hand corner of the main menu), and click on the Fetch/Validate link next to the ORCID field. This will take you to the ORCID site and allow you to create a new iD or authenticate a pre-existing iD in Editorial Manager. Please see the following video for instructions on linking an ORCID iD to your Editorial Manager account: <https://ddec1-0-en-ctp.trendmicro.com:443/wis/clicktime/v1/query?url=https%3a%2f%2fwww.youtube.com%2fwatch%3fv%3d%5fxcclfuvtxQ&umid=1225bc8e-2e69-491e-bcb5-366859b053fc&auth=5a48ddabf21f7246250a6ac00727f7875e94cad3-e811bacb5cd148941b630a28806312c776441b8c>.

*Response: thank you for this comment. ORCID iD has been linked as requested.*

4. We note that you have referenced (Ballew, J.K. (2020) The effects of resiliency training on self-reported compassion fatigue and compassion satisfaction in mental health professionals and counselors-in-training), (Boone, B.C. (2012). The impact of poetry therapy on symptoms of secondary posttraumatic stress disorder in domestic violence counsellors), (Boone, B.C. (2012). The impact of poetry therapy on symptoms of secondary posttraumatic stress disorder in domestic violence counsellors), (Gentry, J. E. (1996). Solution-focused trauma recovery scale (TRS), (Landis. E.M. (2010). Sharevision: A Collarative-Reflective, Expressive Arts Intervention To Address Trauma), ( Ray, C.A. (1981). Holistic stress management training: a burnout strategy for mental health workers), (Ray, C.A. (1981). Holistic stress management training: a burnout strategy for mental health workers), (Walker, D. (2018). Examining the Effects of a Brief Mindfulness-Based Stress Reduction Program on Burnout in Mental Health Professionals), and (Wymer, B. (2019). An Investigation of the Impact of a Supervision Intervention on Secondary Traumatic Stress Responses Among Counselors Treating Child Survivors of Sexual Abuse) which have currently not yet been accepted for publication. Please remove this from your References and amend this to state in the body of your manuscript: (ie “Bewick et al. [Unpublished]”) as detailed online in our guide for authors

*Response: thank you for this comment. These references have been removed from the reference list and cited as unpublished in the body of the text.*

<https://ddec1-0-en-ctp.trendmicro.com:443/wis/clicktime/v1/query?url=http%3a%2f%2fjournals.plos.org%2fplosone%2fs%2fsubmission%2dguidelines%23loc%2dreference%2dstyle&umid=1225bc8e-2e69-491e-bcb5-366859b053fc&auth=5a48ddabf21f7246250a6ac00727f7875e94cad3-ee58d0a4fae79edfbdff72dd03ae511aed1c1ac5>

5. Please remove your figure 2 from within your manuscript file, leaving only the individual TIFF/EPS image files, uploaded separately. These will be automatically included in the reviewers’ PDF.

*Response: Thank you for this comment. Figure 2 has been removed from the manuscript.*

6. Please include a caption for figures 1 and 3.

*Response: thank you for this comment. Figure captions have been added for Figures 1 and 3.*

7. We notice that your supplementary files are included in the manuscript file. Please remove them and upload them with the file type 'Supporting Information'. Please ensure that each Supporting Information file has a legend listed in the manuscript after the references list.

*Thank you for this comment. We have removed supplementary information from the manuscript, added legends and uploaded separately as requested.*
