# Supplementary material for: Interventions to address empathy-based stress in mental health workers: A scoping review and research agenda
Source: PLoS One. 2024 Dec 5;19(12):e0306757. doi: 10.1371/journal.pone.0306757 (PMC11620613; doi:10.1371/journal.pone.0306757)
Supplement: Attachment — Submitted filename: Response to Reviewers1.docx [file pone.0306757.s007.docx]

**Response to Reviewers**

Journal Requirements:

1. Please review your reference list to ensure that it is complete and correct. If you have cited papers that have been retracted, please include the rationale for doing so in the manuscript text, or remove these references and replace them with relevant current references. Any changes to the reference list should be mentioned in the rebuttal letter that accompanies your revised manuscript. If you need to cite a retracted article, indicate the article’s retracted status in the References list and also include a citation and full reference for the retraction notice.

***Response:*** *thank you for raising this important consideration. We can confirm that the original source for each reference cited (unless otherwise specifically stated in the manuscript) was sighted and reviewed by the authors during the process of undertaking the scoping review. Thus, we can confirm that at the point in time that each reference was cited/ added to the manuscript it was complete and accurate, and there was no issue relating to the publication status of any references. To the best of the authors knowledge this continues to be the case and thus we confirm that we have not knowingly cited any papers that have been retracted. Following this comment we have used an AI tool and the reference check function on Scite (*[*https://scite.ai/home*](https://scite.ai/home)*) to check the reference list for any retractions or known issues with cited papers, neither of which flagged any references of concern.*

*However, If the reviewer raising this comment, is aware of a cited paper that has been retracted, we would be grateful if they could please alert us of the specific reference. We will then of course take the steps as they have described to address and amend this.*

Additional Reviewer Comments:

2. The title must include scoping review, as it indicates the type of your papers.

***Response****: thank you for this comment. Please note the words ‘scoping review’ are included in the title, Title: “****Interventions to address empathy-based stress in mental health workers: A scoping review and research agenda****”.*

3. The abstract: The introductory paragraph needs to be more concise and focused;
The conclusion of the study must be written, as must the implications for healthcare organizations. Please find the reference number on page 4, lines 69–72.

***Response:*** *thank you for this comment. We have now made edits to the beginning of the abstract to make it more concise. We have also included a conclusion and implications for healthcare organisations. Regarding the lines quoted, the positioning of reference has been edited for clarity.*

4. Introduction :
The background of the topic needs to be clarified.

***Response:*** *thank you for drawing out attention to this. In order to clarify the background to the topic, we have outlined the context for the review which provides the context for why this is an important issue, what has been done and the existing gaps. We have been mindful to balance this in line with requested amendment no 5 (as per below) and thus have edited the introduction on the pages suggested to ensure it is more concise.*

5. Introduction from pages 2–5 You need to be more concise, as it is very long, to address the key concept of your research.

***Response:*** *thank you for this feedback. We appreciate the introduction is lengthy and we have grappled with the challenge of summarising the large amount of information required to adequately contextualise the review. However, we have now made further edits to pages 2-5 to reduce length and increase clarity.*

6. The words clinician on pages 5 and 6 need to be more categorized into nurses, physicians, and so on.

***Response:*** *thank you for this comment. We have changed the word clinician to be more specific as requested.*

7. The review and aim: The current related literature needs to be supported by other studies in different settings, countries, and sectors (private and governmental).

***Response:*** *thank you for this comment. We have utilised a range of literature from different settings, countries, and sectors. This includes the National Health Service (government run) and academic literature from studies conducted in a range of countries ( across Europe, North America, Asia and Africa) and settings both government and private sector.*

*In line with the aims of the review we have drawn on data from across healthcare fields that have focussed on mental health care settings. Please note that included studies (though all were written in English due to this being a scoping review) were drawn from 10 separate countries and from child and adult MH settings including inpatient, outpatient/community, education, forensics, veterans services, addictions service and domestic violence services.*

8. The aim is Objective No. 5 is very general and cannot be considered an aim as it is an essential part of the research paper.

***Response:*** *thank you for this comment, we have removed this from the manuscript.*

9. The letter t on page 7, line 131, needs to be deleted, as well as repeated (.) in line 135.

***Response:*** *thank you for highlighting this, this has been remedied.*

10. The result needs to be more concise, as it is very long.

***Response:*** *thank you drawing our attention to this. We have further edited the results section to make it more concise, including moving Table 5 to supplementary data. Please note, that the length of the results section reflects both the large number of included records (51) and the significant heterogeneity of the interventions described.*

*In line with methodology of a scoping review the intention is to outline the state of the evidence rather than to identify the most effective form of intervention. Thus, we have endeavoured to succinctly summarise and characterise the widely varying evidence in narrative and table form. We hope that this section is now to your satisfaction, as we believe that this section as it now stands is a significant part of what our review contributes to the understanding of this topic and that if we were to edit it any further it would diminish its usefulness.*

11. Discussion: It needs to be organized according to the most important intervention, as this paper aims to clarify all interventions and identify the most applicable in healthcare.
Recommendations need to be concise, and only applicable recommendations
The paper is very long and needs to be more concise. The authors should choose the most applicable intervention to prevent confusion with the readers.

***Response:*** *thank you for this comment. Our paper is a scoping review, and therefore aims to describe the* ***state of the literature*** *rather than to identify the most important or effective intervention. This is reflected in the 4 aims of the scoping review:*

*“1)	To assess and summarise the available interventions for preventing or treating EBS in MHWs*

*2)	To ascertain the theoretical underpinnings and assumptions of these interventions*

*3)	To assess how EBS has been measured in these studies*

*4)	To make recommendations for future research into the treatment and prevention of EBS in	MHWs”*

*Please note, that these aims are consistent with the purpose and function of a scoping review (as opposed to other forms of systematic reviews/meta-analyses). This is described in the Joanna Briggs Scoping Review Framework (Peters et al., 2017). For ease of reference, we have included a relevant excerpt below:*

*“Unlike systematic reviews, the aim of the scoping reviews is a way of mapping the key concepts that underpin a research area.3 Scoping reviews can be particularly useful for bringing together literature in disciplines with emerging evidence, as they are suited to addressing questions beyond those related to the effectiveness or experience of an intervention. Scoping reviews can be conducted to map a body of literature with relevance to time, location (e.g. country or context), source (e.g. peer-reviewed or grey literature), and origin (e.g. healthcare discipline or academic field).4 The value of scoping reviews to evidence-based practice is the examination of a broader area to identify gaps in the research knowledge base,9 clarify key concepts,10 and report on the types of evidence that address and inform practice in the field… Another distinction between scoping reviews and systematic reviews is that unlike a systematic review, scoping reviews are designed to provide an overview of the existing evidence base regardless of quality.”*

*With reference to the applicability of included information, please note that as outlined in out methods section, an-priori approach was utilised. In our methods section, we have outlined the steps taken via our search strategy to ensure applicability to MHWs and mental health settings.*

12. Consider the previous reviewer comment about the potential conflicts between the needs of health care workers and the needs of the leadership of health care organizations. The authors have stated, "Most studies intervened at the level of the individual, despite the proposed causes of EBS being predominantly organizational." The authors don't address why this is happening. The authors point out that excessive workload is a major contributor to EBS, yet there were no interventions to reduce workload by hiring more healthcare workers. In most circumstances, there are in fact more healthcare workers available to be hired, but doing so is costly. That's the obvious conflict the authors need to address. Organizations usually try to get by with as few workers as possible to save costs. Workers then pay the price by having excessive workloads. If the authors have a different explanation for why organizations don't correct this obvious situation, they should offer it.

***Response****: thank you for drawing our attention to this point. We agree that attempts at cost saving are no doubt a significant factor in why organisations employing MHWs do not use organisational means to reduce EBS e.g. hiring more staff. We have edited the discussion accordingly. Please see pg40-41- where the below section has been included just after the points about organisational vs individual interventions:*

*“It is possible that organisational reluctance to prioritise interventions of this nature is driven at least in part by cost-saving concerns, as meaningfully reducing workloads ultimately means hiring more staff. However, evidence suggests that improving staff engagement and satisfaction leads to better care quality, patient satisfaction, financial performance and staff retention (31 ,32), suggesting that, in the long term, this investment is worth the price.”*

Review Comments to the Author

**Reviewer #1**:

**13.** I congratulate you for this work you have carried out to fill the gap in the literature. I think that this article will be a precursor for future studies. In addition, the results obtained can contribute to institutional managers taking measures to solve problems, supporting employees and creating awareness of empathy based stress among mental health workers , which is a very important issue.

***Response:*** *thank you very much for this feedback, we are delighted by reviewer 1’s comments on the value and important contributions that our paper makes.*
